# Risk factors for thrombolysis-related intracranial hemorrhage: a systematic review and meta-analysis

**DOI:** 10.1186/s12959-023-00467-6

**Published:** 2023-03-14

**Authors:** Jiana Chen, Zhiwei Zeng, Zongwei Fang, Fuxin Ma, Meina Lv, Jinhua Zhang

**Affiliations:** Department of Pharmacy, Fujian Maternity and Child Health Hospital College of Clinical Medicine for Obstetrics & Gynecology and Pediatrics, Fujian Medical University, #18 Daoshan Road, Fuzhou, China

**Keywords:** Thrombolysis, Intracranial hemorrhage, Risk factor, Predict, Meta-analysis

## Abstract

**Background:**

Thrombolysis-related intracranial hemorrhage has a high mortality rate, and many factors can cause intracranial hemorrhage. Until now, systematic reviews and assessments of the certainty of the evidence have not been updated.

**Aim:**

We conducted a systematic review to identify risk factors for thrombolysis-related intracranial hemorrhage.

**Method:**

The protocol for this systematic review was prospectively registered with PROSPERO (CRD42022316160). All English studies that met the inclusion criteria published before January 2022 were obtained from PubMed, EMBASE, Web of Science, and Cochrane Library. Two researchers independently screened articles, extracted data, and evaluated the quality and evidence of the included studies. Risk factors for intracranial hemorrhage were used as the outcome index of this review. Random or fixed-effect models were used in statistical methods.

**Results:**

Of 6083 citations, we included 105 studies in our analysis. For intracranial hemorrhage, moderate-certainty evidence showed a probable association with age, National Institutes of Health stroke scale, leukoaraiosis, hypertension, atrial fibrillation, diabetes, total cholesterol, proteinuria, fibrinogen levels, creatinine, homocysteine, early infarct signs, antiplatelet therapy and anticoagulant therapy; In addition, we found low-certainty evidence that there may be little to no association between risk of intracranial hemorrhage and weight, sex, platelet count, uric acid, albumin and white matter hyperintensity. Leukoaraiosis, cardiovascular disease, total cholesterol, white blood cell count, proteinuria, fibrinogen levels, creatinine, homocysteine and early CT hypodensities are not included in most intracranial hemorrhage risk assessment models.

**Conclusion:**

This study informs risk prediction for thrombolysis-related intracranial hemorrhage, it also informs guidelines for intracranial hemorrhage prevention and future research.

**Supplementary Information:**

The online version contains supplementary material available at 10.1186/s12959-023-00467-6.

## Introduction

Thrombolytic drugs, especially rt-PA (e.g., alteplase), are the most effective pharmacological therapy for acute ischemic stroke (AIS), to increase survival and reduce morbidity [1, 2]. However, the risk of severe hemorrhagic transformation in patients treated with rt-PA also increased [3]. Intracranial hemorrhage (ICH), also called cerebral hemorrhage, is the most serious complication of stroke thrombolysis and an important obstacle to generalized thrombolytic therapy [4]. ICH has been reported in 1.7% to 8.8% of patients with acute ischemic stroke treated with iv thrombolysis, mortality and morbidity rates increase in patients with symptomatic ICH [5–7]. Therefore, it is necessary to accurately predict the bleeding risk of patients, which will help physicians weigh the benefits and risks of thrombolytic therapy and reduce the occurrence of intracranial hemorrhage.

The risk assessment model (RAM) for thrombolysis-related intracranial hemorrhage consists of a combination of multiple predictors. Risk for specified endpoints can be obtained based on the relevant predictors to inform recommendations for strata of patients [8]. In clinical treatment or medication decisions, we can apply relevant models for risk prediction to reduce the occurrence of intracranial hemorrhage. Therefore, establishing and using a thrombolysis-related intracranial hemorrhage model is crucial.

RAMs are currently available for patients on thrombolytic therapy, which can be scored and stratified according to risk factors. Although these models can prevent intracranial hemorrhage to some extent, most of them were developed using existing data that were not based on a systematic review of all potential risk factors [9]. However, model development requires a systematic review to determine the importance of risk factors [9]. Predictors included in existing models were not comprehensive, and effect sizes of the risk factors were not subjected to meta-analysis, which may reduce the predictive power of the model.

Therefore, this review included studies of thrombolysis-related intracerebral hemorrhage models and risk factors to conduct a systematic review and meta-analysis of risk factors for intracerebral bleeding that may inform treatment, future guideline recommendations, and the development of RAMs.

## Method

### Search strategy

The protocol for this systematic review was prospectively registered with PROSPERO (CRD42022316160). Data were reviewed from four databases: PubMed, EMBASE, Web of Science, and the Cochrane Library. Studies in English published before January 2022 were included. The groups of search keywords included were: (1) thrombolysis OR thrombolytic drug OR thrombolytic agent OR fibrinolytic agent OR fibrinolytic drug OR thrombolytic therapy; (2) intracerebral hemorrhage OR ICH OR cerebral hemorrhage OR hemorrhagic infarction OR subarachnoid hemorrhage OR subdural hemorrhage OR epidural hemorrhage; and (3) prediction model OR predict* OR risk prediction OR risk factor. A detailed search strategy is presented in Supplemental Material 1.

### Study selection

Studies were selected independently by two researchers and checked to prevent potential errors. A third independent researcher resolved disputes arising in the process of study selection. Studies that met the following criteria were included: (1) use of thrombolytic drugs [e.g., Tissue plasminogen activator (tPA), urokinase]; (2) comparison between the ICH group and Non-ICH group; and (3) the outcome index was risk factors or predictors. Studies that met the following criteria were excluded: (1) patients with ICH treated with non-thrombolytic drugs; (2) no access to data (including no data related to the risk factors in the study, the study was in the design or recruitment stage, no permission to use the data had been granted, contacted the corresponding author but no reply had been received).

### Data extraction

Data were extracted independently by two researchers and checked to prevent potential errors. A third independent researcher resolved disputes arising in the process of data extraction. The data extracted included the name of the first author, year of publication, time frame, population and their demographics (e.g., sample size, number of centers, age, and sex), study design (e.g., cohort or case–control), type of prediction model study (development, validation, and impact), outcomes and measures of association [e.g., odds ratio (OR) or risk ratio (RR) or hazard ratio (HR), 95% confidence interval (CI) and *P*-value].

### Quality assessment

#### Risk of bias assessment

We assessed the risk of bias in the included studies by using the Prediction Study Risk of Bias Assessment Tool (PROBAST) for RAM studies [10] and the Quality in Prognosis Studies tool (QUIPS) for prognostic factor studies [11–13].

#### Certainty of evidence assessment

We assessed the certainty of the evidence for each of the risk factors per outcome, based on the GRADE approach [14]. The approach considers the following domains: risk of bias, indirectness, inconsistency, imprecision, and publication bias. We developed evidence profiles and rated the overall certainty of evidence as high, moderate, and low or very low, depending on the grading of the individual domains [14]. We narratively described the strength of the association using the terms “there is,” “there probably is,” or “there may be,” depending on whether the quality of the evidence was “high,” “moderate,” or “low/very low,” respectively.

### Statistical analysis

We standardized each risk factor by log-transformation and unifying the direction of the predictors [15]. In studies that reported the measure of association as hazard ratio or risk ratio, we converted them to OR using the baseline risk reported in the studies [16, 17]. We conducted a meta-analysis of associations using the generic inverse variance-based method to produce an overall measure of association. We used the Review Manager 5.3 software for meta-analysis. The statistical indicators were odds ratio (OR) and 95% confidence interval (CI). The Chi-Square test (χ2) test was used to test the heterogeneity of results. If *P* ≥ 0.1 and I^2^ ≤ 50%, the fixed-effect model was used for meta-analysis. The random-effect model was used when *P* < 0.1 and I^2^ > 50%. To explore the stability of the results, we conducted a sensitivity analysis by eliminating studies one by one.

## Results

### The characteristics of included studies

**Fig. 1:**
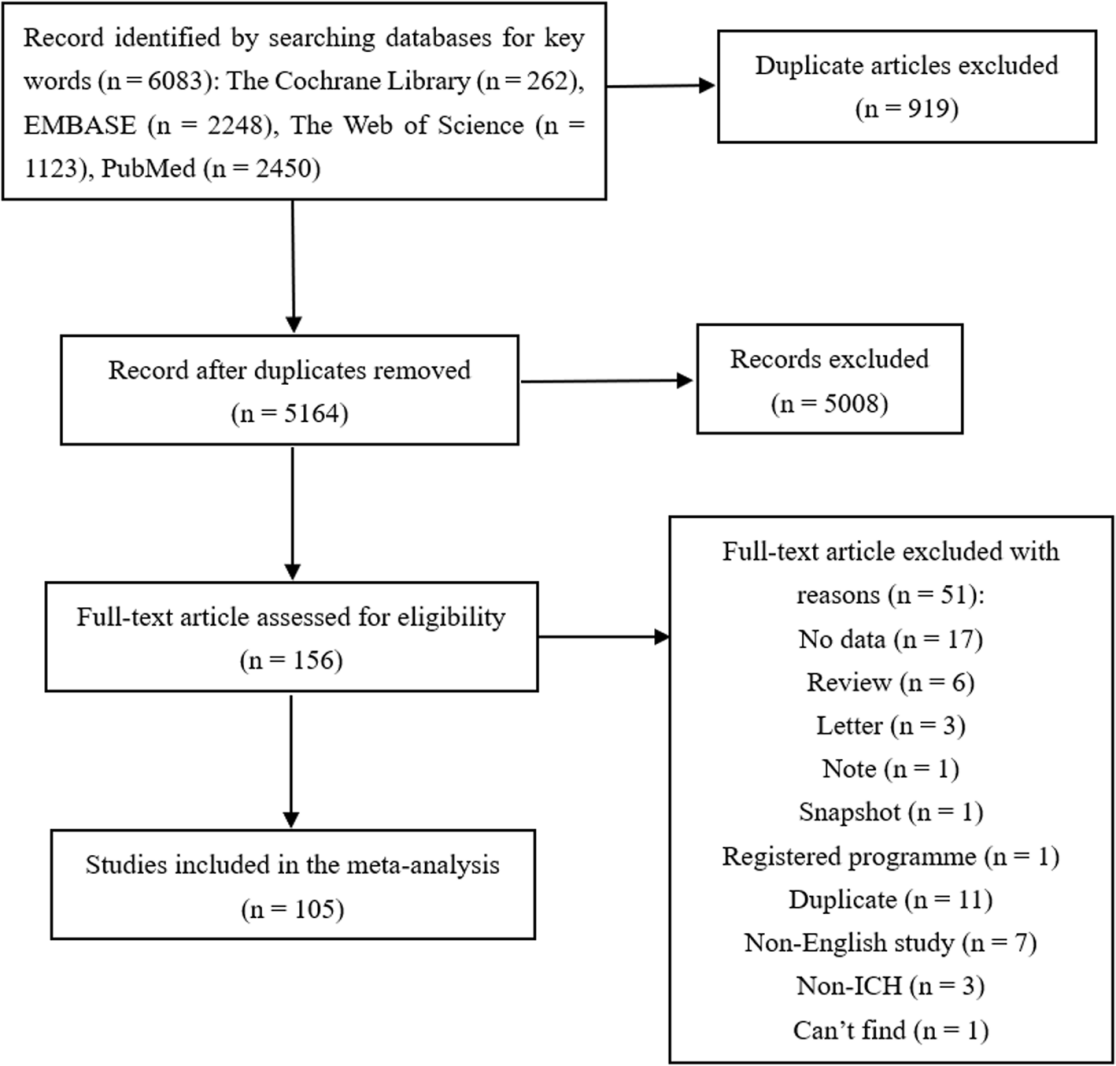
Flow chart and results of literature screening

A total of 6083 articles were retrieved based on the search criteria. After screening, 105 articles met the inclusion criteria and were analyzed [S1-105]. The flow chart and results of the screening are shown in Fig. 1. Supplemental table 1 describes the characteristics of the included studies reporting on the outcomes of ICH. 22 studies were prediction model development studies [S1-22], and 83 were risk factor studies [S23-105]. 95 studies were cohorts [S1, S4-5, S7-49, S51-65, S67-69, S72-78, S80-84, S86-88, S90-105], 43 of which were multicenter [S1, S4-5, S7-15, S19, S21-23, S25, S31, S34-36, S40, S49, S55-58, S61-64, S67, S74-75, S91-94, S100, S102-105]; 10 were case–control studies [S2, S3, S6, S50, S66, S70-71, S79, S85, S89], 6 of which were multicenter [S3, S6, S70, S79, S85, S89]. Most of the patients were between 50 and 80 years old, and most of them were male. Among the 105 studies, the populations of 96 studies were stroke patients [S1-6, S8-20, S22-41, S43-55, S57-66, S68-74, S76-78, S80-84, S86-104], 6 were in patients with myocardial infarction [S21, S56, S67, S75, S79, S85], one was in patients with pulmonary embolism [S7], one was in patients with major artery occlusion [S42], and one was in patients with deep venous thrombosis [S105].

### Risk of bias assessment

The risk of bias was serious across all identified studies, each presenting risk of bias in at least 1 domain or item (Supplemental table 4). Among the 105 included studies, 72 were retrospective, which may have introduced classification bias [S2-3, S5-8, S11-14, S16, S18-20, S22, S24-28, S32-34, S36-38, S40-42, S44-46, S48, S50-51, S53, S55, S58, S60-68, S70-75, S78, S80-83, S86-93, S96, S99, S101-103, S105]. Certainty in evidence was downgraded for imprecision, given that the confidence interval suggests that there may be no association. 9 of the 22 prediction model studies and 4 of the 83 risk factors studies did not have a clear description of appropriate outcome measurement [S5-7, S9-10, S14-16, S21, S34, S41, S75, S105]. Supplemental tables 2 and 3 provide the detailed judgements for each of the risk of bias domain criteria.

### Analysis of risk factors of thrombolysis-related ICH

Investigated were 110 candidate risk factors for ICH from 105 studies. Supplemental table 2 provides the evidence profile for risk factors of thrombolysis-related ICH. Supplemental figure (sFigure) 1–110 provides the forest plots of the meta-analysis of each of the risk factors.

#### Demographic factors

We found moderate-certainty evidence that there is probably an association between risk of ICH and age (OR, 1.77; 95%CI, 1.52–2.07) [S5, S7, S9-10, S12-14, S17, S19, S21, S23, S56, S65, S67, S73-75, S77, S79, S83, S91, S105] and race (OR, 1.86; 95%CI; 1.40–2.45) [S5, S38, S75]. We found low-certainty evidence that there may be little to no association between risk of ICH and body weight < 70 kg (OR, 1.26; 95%CI, 0.96–1.67) [S9-10, S21, S72, S75, S79, S85] and sex (OR, 0.96; 95%CI, 0.63–1.47) [S5, S56, S65, S75, S105]. See sFigure 1–4 for details.

#### Functional factors

We found moderate-certainty evidence that there is probably an association between risk of ICH and the Alberta Stroke Programme Early CT Score (ASPECTS) ≤ 7 (OR, 1.97; 95%CI, 1.25–3.12) [S1-3, S47, S64], National Institutes of Health stroke scale (NIHSS) (OR, 1.27; 95%CI, 1.22–1.33) [S4-6, S8-10, S12-20, S22, S24, S27-30, S36-38, S47, S50, S64-65, S69, S73-74, S77-78, S81, S83-84, S89, S91, S96-97, S99], modified Rankin scale (mRS) > 2 (OR, 1.65; 95%CI, 1.19–2.27) [S15, S53], Thrombolysis in Cerebral Infarction (TICI) score (3, 2, 1, 0; Each one decrease) (OR, 1.82; 95%CI, 1.04–3.18) [S34], CHADS2 score > 2 (OR, 14.00; 95%CI, 1.59–123.28) [S47], low ejection fraction (EF) (OR, 16.22; 95%CI, 2.89–91.03) [S47], higher SEDAN score (OR, 9.25; 95%CI, 2.37–36.10) [S47], arterial stiffness index (ASI) (OR, 1.90; 95%CI, 1.09–3.31) [S54] and K ^trans^ (The contrast volume transfer coefficient) (OR, 5.04; 95%CI, 2.01–12.64) [S102]. We found low-certainty evidence that there may be little to no association between risk of ICH and apparent diffusion coefficient (ADC) (OR, 2.72; 95%CI, 0.43–17.14) [S40, S42]. See sFigure 5–14 for details.

#### Medical illness and patient history factors

We found moderate-certainty evidence that there is probably an association between risk of ICH and peripheral vascular disease (PVD) (OR, 1.59; 95%CI, 1.12–2.26) [S7], cerebral small vascular diseases (CSVD) (OR, 2.69; 95%CI, 1.98–3.66) [S20, S38, S70-71, S100], cerebral microbleeds (OR, 2.72; 95%CI, 1.45–5.10) [S38], leukoaraiosis (OR, 2.61; 95%CI, 1.74–3.91) [S70-71, S100], poor collaterals (OR, 4.36; 95%CI, 1.82–10.41) [S46, S90], recent facial or head trauma (2 weeks) (OR, 13.00; 95%CI, 3.40–49.70) [S85], cerebral artery occlusion (OR, 8.52; 95%CI, 3.20–22.64) [S37] and decreased levels of consciousness (OR, 2.36; 95%CI, 1.51–3.68) [S33, S37, S101]. We found low-certainty evidence that there may be little to no association between risk of ICH and stroke (OR, 4.68; 95%CI, 1.49–14.70) [S7, S10, S38, S47, S56, S58, S61, S75, S100, S105]. See sFigure 15–23 for details.

We found moderate-certainty evidence that there is probably an association between risk of ICH and cardiovascular disease (OR, 2.09; 95%CI, 1.75–2.49) [S4, S7-9, S13-14, S16, S19-23, S30, S37, S47, S58, S61, S77, S83, S91], prior myocardial infarction (OR, 1.80; 95%CI, 1.33–2.44) [S7], valvular heart diseases (OR, 2.09; 95%CI, 1.07–4.08) [S8], hypertension (OR, 1.42; 95%CI, 1.21–1.67) [S9, S13-14, S19-21, S58], atrial fibrillation (AF) (OR, 2.62; 95%CI, 1.92–3.59) [S4, S13-14, S16, S19-20, S22, S30, S37, S47, S61, S77, S83], congestive heart failure (OR, 2.57; 95%CI, 1.16–5.69) [S23] and diabetes (OR, 1.84; 95%CI, 1.34–2.51) [S13-14, S20, S90-91]. We found low-certainty evidence that there may be little to no association between risk of ICH and dyslipidemia (OR, 1.18; 95%CI, 0.57–2.47) [S19, S74, S87] and visual field deficits (OR, 1.07; 95%CI, 0.29–3.91) [S33]. In addition, we found very low-certainty evidence that there may be little to no association between risk of ICH and smoke (OR, 0.47; 95%CI, 0.02–14.61) [S77, S86]. See sFigure 24–33 for details.

#### Laboratory and physical examination factors

We found high-certainty evidence that there is an association between risk of ICH and thrombin-activated fibrinolysis inhibitor (TAFI) (OR, 12.90; 95%CI, 1.41–118.01) [S39] and plasminogen activator inhibitor (PAI)-1 (OR, 12.75; 95%CI, 1.17–138.95) [S39]. We found moderate-certainty evidence that there is probably an association between risk of ICH and blood sugar (OR, 1.14; 95%CI, 1.10–1.20) [S1, S5-6, S9-10, S12, S16-19, S22, S30, S32, S34, S47, S57, S65, S83-84, S91, S97], Platelet derived growth factor-CC (PDGF-CC) (OR, 1.03; 95%CI, 1.00–1.06) [S98], blood pressure (OR, 2.59; 95%CI, 1.07–6.27) [S99], Systolic blood pressure (SBP) (OR, 1.15; 95%CI, 1.10–1.20) [S5, S8-10, S12, S17, S19, S23, S28, S36, S56, S75-77, S97, S99], pulse pressure (OR, 2.37; 95%CI, 1.01–5.57) [S19, S67], international normalized ratio (INR) (OR, 2.47; 95%CI, 1.34–4.55) [S30, S75] and activated partial thromboplastin time (APTT) (OR, 2.13; 95%CI, 1.02–4.45) [S86]. We found low-certainty evidence that there may be little to no association between risk of ICH and mean platelet volume (MPV) (OR, 1.02; 95%CI, 1.00–1.04) [S50] and prothrombin time activity percentage (PTA) (OR, 1.02; 95%CI, 1.00–1.03) [S78]. See sFigure 34–44 for details.

We found moderate-certainty evidence that there is probably an association between risk of ICH and total cholesterol (TC) (OR, 0.91; 95%CI, 0.83–0.99) [S31], low density lipoprotein cholesterol (LDL-C) (OR, 0.87; 95%CI, 0.82–0.92) [S31, S50], high density lipoprotein cholesterol (HDL-C) (OR, 1.09; 95%CI, 1.01–1.18) [S31], triglyceride (TG) (OR, 0.84; 95%CI, 0.72–0.97) [S31], TC/HDL-C (OR, 1.73; 95%CI, 1.01–2.96) [S51], TG/HDL-C (OR, 2.06; 95%CI, 1.24–3.43) [S51], LDL-C/HDL-C (OR, 1.93; 95%CI, 1.07–3.50) [S51], white blood cell count (OR, 1.10; 95%CI, 1.01–1.19) [S78], absolute eosinophil count (AEC) (OR, 0.22; 95%CI, 0.07–0.72) [S81] and low serum-free triiodothyronine (fT3) (OR, 0.24; 95%CI, 0.11–0.51) [S26, S53]. We found low-certainty evidence that there may be an association between risk of ICH and neutrophil to lymphocyte ratio (NLR) (OR, 1.09; 95%CI, 1.00–1.18) [S22, S104]. See sFigure 45–55 for details.

We found high-certainty evidence that there is an association between risk of ICH and activated protein C (APC) (OR, 25.19; 95%CI, 4.76–133.3) [S59]. We found moderate-certainty evidence that there is probably an association between risk of ICH and albuminuria (OR, 1.66; 95%CI, 1.20–2.28) [S44, S49], fibrinogen (FIB) (OR, 6.64; 95%CI, 3.40–12.97) [S47, S73, S86, S88-89], fibrinogen degradation products (FDP) (OR, 7.50; 95%CI, 1.26–44.64) [S88], globulin (OR, 1.18; 95%CI, 1.09–1.29) [S78], caveolin (OR, 2.35; 95%CI, 1.71–3.24) [S52, S77], matrix metalloproteinase-9 (MMP9) / tissue inhibitor of metalloproteinases (TIMP) (OR, 1.72; 95%CI, 1.31–2.28) [S63], S100B (OR, 2.80; 95%CI, 1.40–5.60) [S92], cellular fibronectin (c-Fn) (OR, 2.10; 95%CI, 1.30–3.39) [S94], glomerular filtration rate (GFR) (OR, 1.83; 95%CI, 1.38–2.43) [S80, S105], creatinine (OR, 5.50; 95%CI, 1.08–28.01) [S93], homocysteine (OR, 13.65; 95%CI, 3.58–52.05) [S96], apelin (OR, 0.24; 95%CI, 0.09–0.68) [S103], Interleukin-1β (IL-1β) (OR, 1.06; 95%CI, 1.03–1.09) [S103], Interleukin-6 (IL-6) (OR, 1.53; 95%CI, 1.12–2.11) [S103], malondialdehyde (MDA) (OR, 2.49; 95%CI, 1.32–4.70) [S103] and superoxide dismutase (SOD) (OR, 0.20; 95%CI, 0.05–0.75) [S103]. See sFigure 56–72 for details.

We found low-certainty evidence that there may be little to no association between risk of ICH and platelet count (OR, 1.00; 95%CI, 0.98–1.01) [S6, S8, S15, S18, S28-29, S36, S86, S90], uric acid (UA) (OR, 1.00; 95%CI, 0.99–1.00) [S50], diastolic blood pressure (DBP) (OR, 1.43; 95%CI, 0.89–2.31) [S4, S85] and albumin (OR, 2.30; 95%CI, 0.89–6.00) [S50, S62, S95]. We found very low-certainty evidence that there may be little to no association between risk of ICH and glycated hemoglobin A1c (HbA1c) (OR, 3.36; 95%CI, 0.50–22.56) [S27, S62] and mean artery pressure (MAP) (OR, 3.68; 95%CI, 0.61–22.13) [S32, S47, S73]. See sFigure 73–78 for details.

We found moderate-certainty evidence that there is probably an association between risk of ICH and early computed tomography (CT) hypodensities (OR, 2.47; 95%CI, 1.54–3.95) [S23, S33], hyperdense middle cerebral artery (HDMCA) sign (OR, 1.57; 95%CI, 1.09–2.25) [S12, S33, S48, S76, S83], early infarct signs (OR, 2.86; 95%CI, 1.30–6.33) [S12, S32, S83], fluid-attenuated inversion recovery (FLAIR) hyperintensity (OR, 13.58; 95%CI, 3.72–49.60) [S25, S60], early CT signs of cerebral ischaemia (OR, 3.40; 95%CI, 2.19–5.27) [S6, S46, S55], brain infarction volume (OR, 1.81; 95%CI, 1.25–2.62) [S24, S35, S69], high-permeability region size on PCT (HPrs-PCT) (OR, 1.00; 95%CI, 1.00–1.00) [S29], supratentorial territory of the posterior cerebral artery (PCA) (OR, 4.31; 95%CI, 1.20–15.49) [S37], cerebral blood volume (CBV) (OR, 100.00; 95%CI, 3.20–3125.31) [S72] and calcification volume on the lesion side (CV-L) (OR, 1.50; 95%CI, 1.14–1.98) [S66]. We found low-certainty evidence that there may be little to no association between risk of ICH and white matter hyperintensity (WMH) (OR, 2.45; 95%CI, 0.95–6.32) [S45, S68]. See sFigure 79–89 for details.

We found moderate-certainty evidence that there is probably an association between risk of ICH and MMP-9-1562C/T polymorphism genotypes (OR, 13.08; 95%CI, 1.04–164.51) [S41], PAI-1 5G/5G genotype (OR, 4.75; 95%CI, 1.18–19.12) [S87], rs1801020, C allele (OR, 2.04; 95%CI, 1.38–3.01) [S4] and rs669, A allele (OR, 2.19; 95%CI, 1.57–3.06) [S4]. See sFigure 90–93 for details.

#### Medication factors

We found moderate-certainty evidence that there is probably an association between risk of ICH and antithrombotic therapy (OR, 2.28; 95%CI, 1.81–2.87) [S2, S8-10, S12, S19, S21, S23, S32, S42, S46-47, S64, S75, S79, S84]. We found moderate-certainty evidence that there is probably an association between risk of ICH and thrombolytic therapy (OR, 2.00; 95%CI, 1.36–2.94) [S2, S21, S23, S42, S46, S75, S84]. Subgroup analysis showed that t-PA (OR, 2.33; 95%CI, 1.54–3.50) [S2, S21, S23, S42, S75, S84] and urokinase (OR, 1.06; 95%CI, 1.01–1.11) [S46] were statistically significant. We found moderate-certainty evidence that there is probably an association between risk of ICH and antiplatelet therapy (OR, 2.15; 95%CI, 1.70–2.72) [S8-10, S12, S32, S47, S64]. Subgroup analysis showed that single antiplatelet therapy (SAPT) (OR, 1.72; 95%CI, 1.52–1.93) [S8-10, S12, S32, S64] and dual antiplatelet therapy (DAPT) (OR, 3.54; 95%CI, 1.86–6.76) [S9, S12, S64] were statistically significant. We found moderate-certainty evidence that there is probably an association between risk of ICH and anticoagulant therapy (OR, 4.40; 95%CI, 1.38–14.01) [S12, S47, S79]. See sFigure 94–97 for details.

We found moderate-certainty evidence that there is probably an association between risk of ICH and antihypertensive drugs (OR, 1.63; 95%CI, 1.24–2.16) [S8, S36], lipid-lowering drugs (OR, 3.23; 95%CI, 2.33–4.48) [S17, S82, S99], microcatheter injection (MCI) (OR, 3.60; 95%CI, 1.12–11.57) [S34], additional endovascular therapy (OR, 8.71; 95%CI, 2.54–29.89) [S37], deviation from the protocol (OR, 11.10; 95%CI, 2.40–51.34) [S55], periventricular transit time to the peak (TTP) (OR, 4.74; 95%CI, 1.62–13.83) [S69] and vaspin (OR, 0.26; 95%CI, 0.12–0.58) [S103]. We found low-certainty evidence that there may be little to no association between risk of ICH and time from onset to treatment (OTT) (OR, 1.06; 95%CI, 0.99–1.15) [S4, S9, S12, S20, S90]. In addition, we found very low-certainty evidence that there may be little to no association between risk of ICH and time to recanalization (OR, 3.19; 95%CI, 0.18–55.75) [S18, S43]. See sFigure 98–106 for details.

#### Other factors

We found moderate-certainty evidence that there is probably an association between risk of ICH and age & NIHSS (OR, 4.08; 95%CI, 2.69–6.18) [S11, S19], age & hypertension (OR, 2.10; 95%CI, 1.02–4.31) [S19] and age & DBP (OR, 6.10; 95%CI, 2.30–16.18) [S19]. We found low-certainty evidence that there may be little to no association between risk of ICH and age & weight (OR, 2.40; 95%CI, 0.90–6.40) [S19]. See sFigure 107–110 for details.

### Sensitivity analysis

Sensitivity analysis was conducted by eliminating studies one by one. There were no significant changes in the outcome except for body weight, ADC, dyslipidemia, smoke, HbA1c, DBP, MAP, albumin, early CT hypodensities, HDMCA sign, early infarct signs, brain infarction volume, WMH, anticoagulant therapy and time to recanalization, indicating that most of the results were stable.

## Discussion

### Summary of findings

We evaluated 110 risk factors for thrombolysis-related ICH. We found high-certainty evidence that there is an association between the risk of ICH and TAFI, PAI-1 and APC. We also identified several statistically significant predictors, such as age, age & NIHSS, ASPECT, NIHSS, cerebral small vascular diseases (CSVD), leukoaraiosis, cardiovascular disease, hypertension, AF, diabetes, blood sugar, SBP, INR, TC, LDL-C, HDL-C, TG, NLR, white blood cell count, AEC, low fT3, albuminuria, FIB, GFR, creatinine, homocysteine, early CT hypodensities, HDMCA sign, early infarct signs, FLAIR hyperintensity, early CT signs of cerebral ischemia, thrombolytic therapy, antiplatelet therapy, anticoagulant therapy, antihypertensive drugs and lipid-lowering drugs, which supported by moderate certainty of the evidence. And low-certainty evidence suggests that body weight, sex, sex & body weight, dyslipidemia, visual field deficits, platelet count, UA, DBP, albumin, WMH and time from onset to treatment (OTT) were not statistically significant. We found very low-certainty evidence that there may be little to no association between risk of ICH and ADC, smoke, HbA1c, MAP and time to recanalization. Therefore, in addition to thrombolytic therapy can affect ICH, other risk factors such as blood sugar, SBP, INR, TC, LDL-C, HDL-C, TG, fT3, albuminuria, GFR, creatinine, homocysteine, antiplatelet therapy, anticoagulant therapy, antihypertensive drugs and lipid-lowering drugs should also be paid attention to during treatment as a way to reduce the occurrence of ICH. We summarize and group (treatable vs. non-treatable) the different certainty-risk factors into a new table to permit easy reading. Please see Supplementary Table 5 for details.

### Implications for practice

Our study identified candidate risk factors for ICH, such as age, body weight, NIHSS, age & NIHSS, diabetes, hypertension, AF, blood sugar, platelet count, SBP, time from onset to treatment (OTT) and antiplatelet therapy that have been considered in the analysis of some developed and widely used RAMs in daily practice, such as the GRASPS, SICH, SITs, SITs-MOST, SPAN-100, STARTING-SICH, THRIVE-C and RICH models [S5, S8-11, S12, S14, S17]. However, some factors that we identified as having a probable association with ICH, based on our meta-analysis results, were not included or considered in the development of most of the RAMs, such as stroke, decreased levels of consciousness, cerebral artery occlusion, poor collaterals, leukoaraiosis, early CT hypodensities, HDMCA sign, FLAIR hyperintensity, LDL-C, INR, brain infarction volume, low fT3, albuminuria, FIB, GFR and anticoagulant therapy. Researchers can add the above risk factors to the data collection process to create a complete clinical prediction model.

We found that congestive heart failure was associated with an increased risk of ICH. However, it should be noted that congestive heart failure has not been previously considered a risk factor for ICH. Patients with congestive heart failure are at high risk of stroke [S106]. A likely mechanism of stroke in these patients is cerebral embolism from a ventricular thrombus, which leads to ICH [S107]. In addition, the relationship between abnormal lipid metabolism and ICH remains controversial. Some studies [S108-109] reported no association with a higher risk of ICH, while others [S110] observed lower LDL-C levels to portend higher rates of ICH. This may be because low levels of lipids will adversely affect the integrity of small vessels in the brain, leading to blood extravasation due to the compromised integrity of the microvascular endothelial cells [S111-112].

We found that smoking may not be related to ICH. This is mainly because Wang et al. found that smoking benefits ICH [S86]. This contrasts with another article that considers smoking harmful to ICH [S77]. Wang et al.'s study about smoking has limitations. The smoking rate in the study population was low, and the distribution was uneven among the patients, which may result in smoking as a protective factor. Given the harmful effects of smoking on health, smoking cessation should still be strongly recommended to prevent stroke. First, long-term smoking can make the adrenal gland release adrenaline, with increases blood pressure and lead to ICH.

Additionally, nicotine in tobacco could directly destroy vascular endothelial function and arterial elasticity, promoting the destruction of the blood–brain barrier and increasing the possibility of ICH. The correlative study demonstrated a strong dose–response between the number of cigarettes smoked daily and ischemic stroke among young men [S113]. Likewise, there is evidence for a dose–response between cigarette smoking and the risk of stroke in middle-aged and older adults [S114].

Although this study did not find a correlation between UA and ICH, Song et al. reported a dose–response relationship between UA and ICH in a cohort of 1230 patients. Higher serum UA was independently related to a lower risk of ICH [S115]. This is an interesting discovery. Only a few studies have investigated the relationship between UA and ICH. It may be related to UA's ability to scavenging of free radicals, inhibit inflammatory cascade reactions, prevent mitochondrial damage, suppress lipid peroxidation, and reduce BBB permeability [S116-117].

In addition, no significant gender difference in ICH was found in this study. In some studies, male sex is a risk factor for developing ICH [S118], but the exact mechanism is unknown. However, other studies have reported that women have a higher risk of ICH [S119]. It may be caused by an inherent biological vulnerability of women for ICH. What’s more, weight and gender had similar results. Some studies suggest that higher body weight may be associated with ICH. Kim et al. found that lower body weight was associated with ICH and explained it as a "paradoxical effect of obesity" [S120]. Some scholars also found a potential explanation: when calculating the rt-PA dose, doctors overestimated their weight in the hyperacute phase, and patients' "overdose" led to ICH. I think we should focus on body mass index (BMI) rather than weight. BMI can avoid the diagnostic error caused by abnormal height (too high or too short) and objectively evaluate the treatment effect.

The mechanism of thrombolytic therapy-induced hemorrhage for cerebral infarction may differ from that of thrombolytic therapy-induced hemorrhage for other disorders. Compared with the mechanism of thrombolysis for bleeding in other diseases, the causes of bleeding in thrombolytic therapy-induced hemorrhage may also include destruction of the blood–brain barrier and reperfusion of injured brain tissue [18]. Therefore, because the bleeding mechanism is not identical, the risk of intracranial hemorrhage may change and is worth continuing to explore in future studies.

### Strengths

Our study followed rigorous methods, conducted extensive searches, duplicate and independent screening and data extraction, and assessed the certainty of evidence based on a structured framework. Also, we conducted sensitivity analyses to determine the stability of the results. The greatest advantage is the comprehensiveness of the study results, which may have some clinical significance in preventing the occurrence of thrombolysis-related ICH.

### Limitations and challenges

Since most of the studies included in this review were retrospective studies, classification and recall bias may lead to potential limitations. And this is not individual patient data meta-analysis and that any associations identified are univariate, with their inherent limitations. In addition, potential limitations of the included studies related to the inconsistency and variability across eligibility criteria in the original studies and variability in study design, study type, sample size, and definitions of the risk factors. Therefore, more rigorous and large-scale studies are needed to confirm our findings, and further analysis is necessary to provide a more reliable basis for clinical work.

## Conclusion

In this systematic review, we identified all reported risk factors for ICH associated with thrombolysis therapy. Some of these factors are not included in current ICH risk prediction models. Our findings will help inform experts in developing population-based guidelines and accurate, user-friendly RAMs to better guide individual patient prophylactic management.

## Supplementary Information

**Additional file 1: Supplemental material 1**. Search strategy

## Data Availability

The datasets generated during and/or analysed during the current study are available from the corresponding author on reasonable request.
