# Supplementary material for: Risk factors for thrombolysis-related intracranial hemorrhage: a systematic review and meta-analysis
Source: Thromb J. 2023 Mar 14;21:27. doi: 10.1186/s12959-023-00467-6 (PMC10012586; doi:10.1186/s12959-023-00467-6)
Supplement: Supplementary file 1 — Additional file 1: Supplemental material 1. Search strategy [file 12959_2023_467_MOESM1_ESM.docx]

**Supplemental material 1--Search strategy**

1. **PubMed**

((((((((((Fibrinolytic Agents[MeSH Terms])) OR (Thrombolytic Therapy[MeSH Terms])) OR (Fibrinolytic Agent[Title/Abstract])) OR (Fibrinolytic Drug[Title/Abstract])) OR (Thrombolytic Drug[Title/Abstract])) OR (Thrombolytic Agent[Title/Abstract])) OR (Antithrombotic Agent[Title/Abstract])) OR (Antithrombic Drug[Title/Abstract])) OR (Thrombolytic Therapy[Title/Abstract])) OR (Therapeutic Thrombolysis[Title/Abstract])) OR (Fibrinolytic Therapy[Title/Abstract])

**56513**

((((((((((((((((((((((((((((((((((Hemorrhagic cerebrovascular disease[Title/Abstract]) OR (Cerebral Hemorrhage[MeSH Terms])) OR (cerebral hemorrhage[Title/Abstract])) OR (Cerebrum Hemorrhage[Title/Abstract])) OR (Intracerebral Hemorrhage[Title/Abstract])) OR (ICH[Title/Abstract])) OR (Cerebral Brain Hemorrhage[Title/Abstract])) OR (Cerebral Parenchymal Hemorrhage[Title/Abstract])) OR (Spontaneous intracerebral hemorrhage[Title/Abstract])) OR (Hemorrhagic infarction[Title/Abstract])) OR (HI[Title/Abstract])) OR (Hemorrhagic cerebral infarction[Title/Abstract])) OR (Cerebral infarction with hemorrhagic transformation[Title/Abstract])) OR (hemorrhagic transformation[Title/Abstract])) OR (Subarachnoid Hemorrhage[MeSH Terms])) OR (Subarachnoid Hemorrhage[Title/Abstract])) OR (SAH[Title/Abstract])) OR (Hematoma, Subdural[MeSH Terms])) OR (hematoma, subdural[Title/Abstract])) OR (Subdural Hematoma[Title/Abstract])) OR (Subdural Hemorrhage[Title/Abstract])) OR (Hematoma, Epidural, Cranial[MeSH Terms])) OR (Hematoma, Epidural, Cranial[Title/Abstract])) OR (Epidural hematoma[Title/Abstract])) OR (Epidural hemorrhage[Title/Abstract])) OR (EDH[Title/Abstract])) OR (Cranial Epidural Hemorrhage[Title/Abstract])) OR (Cranial Epidural Hematoma[Title/Abstract])) OR (Cranial Extradural Hematoma[Title/Abstract])) OR (Cranial Extradural Hemorrhage[Title/Abstract])) OR (Intracranial Epidural Hematoma[Title/Abstract])) OR (Intracranial Hemorrhages[MeSH Terms])) OR (Intracranial Hemorrhage[Title/Abstract])) OR (Cerebral Microbleeds[Title/Abstract])) OR (CMBs[Title/Abstract])

**134613**

(((((((((((Prediction model[Title/Abstract]) OR (Predicting[Title/Abstract])) OR (Model[Title/Abstract])) OR (Risk Factors[MeSH Terms])) OR (Risk[MeSH Terms])) OR (Predicting risk[Title/Abstract])) OR (Risk prediction[Title/Abstract])) OR (Risk Factor[Title/Abstract])) OR (Risk Score[Title/Abstract])) OR (Risk Factor Score[Title/Abstract])) OR (Risk[Title/Abstract])) OR (Relative Risk[Title/Abstract])

**5140838**

**2450**

1. **Cochrane Library**

[Title and abstract]

“Fibrinolytic Agent” OR “Fibrinolytic Drug” OR “Thrombolytic Drug” OR “Thrombolytic Agent” OR “Antithrombotic Agent” OR “Antithrombic Drug” OR “Thrombolytic Therapy” OR “Therapeutic Thrombolysis” OR “Fibrinolytic Therapy”

**3854**

[MeSH]

Fibrinolytic Agents  **2291**

Thrombolytic Therapy  **1746**

**5384**

[Title and abstract]

“Hemorrhagic cerebrovascular disease” OR “Cerebral Hemorrhage” OR “Cerebrum Hemorrhage” OR “Intracerebral Hemorrhage” OR “ICH” OR “Cerebral Brain Hemorrhage” OR “Cerebral Parenchymal Hemorrhage” OR “Spontaneous intracerebral hemorrhage” OR “Hemorrhagic infarction” OR HI OR “Hemorrhagic cerebral infarction” OR “Cerebral infarction with hemorrhagic transformation” OR “hemorrhagic transformation” OR “Subarachnoid Hemorrhage” OR SAH OR “Hematoma, Subdural” OR “Subdural Hematoma” OR “Subdural Hemorrhage” OR “Hematoma, Epidural, Cranial” OR “Epidural hematoma” OR “Epidural hemorrhage” OR EDH OR “Cranial Epidural Hemorrhage” OR “Cranial Epidural Hematoma” OR “Cranial Extradural Hematoma” OR “Cranial Extradural Hemorrhage” OR “Intracranial Epidural Hematoma” OR “Intracranial Hemorrhage” OR “Cerebral Microbleeds” OR CMBs

**12402**

[MeSH]

Cerebral Hemorrhage  **1067**

Subarachnoid Hemorrhage  **616**

Hematoma, Subdural  **138**

Hematoma, Epidural, Cranial  **13**

Intracranial Hemorrhages  **2098**

**12539**

[Title and abstract]

“Prediction model” OR Predicting OR Model OR “Predicting risk” OR “Risk prediction” OR “Risk Factor” OR “Risk Score” OR “Risk Factor Score” OR Risk OR “Relative Risk”

**332709**

[MeSH]

Risk Factors  **25873**

Risk  **39036**

**335238**

**Finally, there are 276 articles, of which 262 are trials**

1. **Embase**

[Title and abstract]

Fibrinolytic Agent OR Fibrinolytic Drug OR Thrombolytic Drug OR Thrombolytic Agent OR Antithrombotic Agent OR Antithrombic Drug OR Thrombolytic Therapy OR Therapeutic Thrombolysis OR Fibrinolytic Therapy

**22253**

[MeSH]

Fibrinolytic Agent  **157972**

fibrinolytic therapy  **27095**

**171782**

[Title and abstract]

Hemorrhagic cerebrovascular disease OR Cerebral Hemorrhage OR Cerebrum Hemorrhage OR Intracerebral Hemorrhage OR ICH OR brain hemorrhage OR Cerebral Brain Hemorrhage OR Cerebral Parenchymal Hemorrhage OR Spontaneous intracerebral hemorrhage OR Hemorrhagic infarction OR HI OR Hemorrhagic cerebral infarction OR Cerebral infarction with hemorrhagic transformation OR hemorrhagic transformation OR Subarachnoid Hemorrhage OR SAH OR Hematoma, Subdural OR Subdural Hematoma OR Subdural Hemorrhage OR Hematoma, Epidural, Cranial OR Epidural hematoma OR Epidural hemorrhage OR EDH OR Cranial Epidural Hemorrhage OR Cranial Epidural Hematoma OR Cranial Extradural Hematoma OR Cranial Extradural Hemorrhage OR Intracranial Epidural Hematoma OR Intracranial Hemorrhage OR Cerebral Microbleeds OR CMBs

**148869**

[MeSH]

brain hemorrhage  **163442**

Subarachnoid Hemorrhage  **49203**

subdural hematoma  **20300**

epidural hematoma  **8537**

**250645**

[Title and abstract]

Prediction model OR Predicting OR Predicting risk OR Risk prediction OR Risk prediction model OR Risk Factor OR Risk Score OR Risk Factor Score OR Relative Risk

**811704**

[MeSH]

Risk prediction model  **56**

Risk prediction  **28**

Prediction model  **120**

Risk Factor  **1180169**

Risk Score  **49**

**1702700**

**2248**

1. **Web of science**

“Fibrinolytic Agent” OR “Fibrinolytic Drug” OR “Thrombolytic Drug” OR “Thrombolytic Agent” OR “Antithrombotic Agent” OR “Antithrombic Drug” OR “Thrombolytic Therapy” OR “Therapeutic Thrombolysis” OR “Fibrinolytic Therapy”

“Hemorrhagic cerebrovascular disease” OR “Cerebral Hemorrhage” OR “Cerebrum Hemorrhage” OR “Intracerebral Hemorrhage” OR “ICH” OR “Cerebral Brain Hemorrhage” OR “Cerebral Parenchymal Hemorrhage” OR “Spontaneous intracerebral hemorrhage” OR “Hemorrhagic infarction” OR HI OR “Hemorrhagic cerebral infarction” OR “Cerebral infarction with hemorrhagic transformation” OR “hemorrhagic transformation” OR “Subarachnoid Hemorrhage” OR SAH OR “Hematoma, Subdural” OR “Subdural Hematoma” OR “Subdural Hemorrhage” OR “Hematoma, Epidural, Cranial” OR “Epidural hematoma” OR “Epidural hemorrhage” OR EDH OR “Cranial Epidural Hemorrhage” OR “Cranial Epidural Hematoma” OR “Cranial Extradural Hematoma” OR “Cranial Extradural Hemorrhage” OR “Intracranial Epidural Hematoma” OR “Intracranial Hemorrhage” OR “Cerebral Microbleeds” OR CMBs

“Prediction model” OR Predicting OR Model OR “Predicting risk” OR “Risk prediction” OR “Risk Factor” OR “Risk Score” OR “Risk Factor Score” OR Risk OR “Relative Risk”

**1123**

**PRISMA 2020 Checklist**

| **Section and Topic** | **Item #** | **Checklist item** | **Location where item is reported** |
| --- | --- | --- | --- |
| **TITLE** | | |  |
| Title | 1 | Identify the report as a systematic review. | Page 1 |
| **ABSTRACT** | | |  |
| Abstract | 2 | See the PRISMA 2020 for Abstracts checklist. | Page 1-2 |
| **INTRODUCTION** | | |  |
| Rationale | 3 | Describe the rationale for the review in the context of existing knowledge. | Page 2 |
| Objectives | 4 | Provide an explicit statement of the objective(s) or question(s) the review addresses. | Page 2 |
| **METHODS** | | |  |
| Eligibility criteria | 5 | Specify the inclusion and exclusion criteria for the review and how studies were grouped for the syntheses. | Page 3 |
| Information sources | 6 | Specify all databases, registers, websites, organisations, reference lists and other sources searched or consulted to identify studies. Specify the date when each source was last searched or consulted. | Page 3 |
| Search strategy | 7 | Present the full search strategies for all databases, registers and websites, including any filters and limits used. | Supplementary materials |
| Selection process | 8 | Specify the methods used to decide whether a study met the inclusion criteria of the review, including how many reviewers screened each record and each report retrieved, whether they worked independently, and if applicable, details of automation tools used in the process. | Page 3 |
| Data collection process | 9 | Specify the methods used to collect data from reports, including how many reviewers collected data from each report, whether they worked independently, any processes for obtaining or confirming data from study investigators, and if applicable, details of automation tools used in the process. | Page 3 |
| Data items | 10a | List and define all outcomes for which data were sought. Specify whether all results that were compatible with each outcome domain in each study were sought (e.g. for all measures, time points, analyses), and if not, the methods used to decide which results to collect. | Page 3 |
|  | 10b | List and define all other variables for which data were sought (e.g. participant and intervention characteristics, funding sources). Describe any assumptions made about any missing or unclear information. | Page 3 |
| Study risk of bias assessment | 11 | Specify the methods used to assess risk of bias in the included studies, including details of the tool(s) used, how many reviewers assessed each study and whether they worked independently, and if applicable, details of automation tools used in the process. | Page 3 |
| Effect measures | 12 | Specify for each outcome the effect measure(s) (e.g. risk ratio, mean difference) used in the synthesis or presentation of results. | Page 3 |
| Synthesis methods | 13a | Describe the processes used to decide which studies were eligible for each synthesis (e.g. tabulating the study intervention characteristics and comparing against the planned groups for each synthesis (item #5)). | Page 3 |
|  | 13b | Describe any methods required to prepare the data for presentation or synthesis, such as handling of missing summary statistics, or data conversions. | Page 3-4 |
|  | 13c | Describe any methods used to tabulate or visually display results of individual studies and syntheses. | Page 4 |
|  | 13d | Describe any methods used to synthesize results and provide a rationale for the choice(s). If meta-analysis was performed, describe the model(s), method(s) to identify the presence and extent of statistical heterogeneity, and software package(s) used. | Page 4 |
|  | 13e | Describe any methods used to explore possible causes of heterogeneity among study results (e.g. subgroup analysis, meta-regression). | None |
|  | 13f | Describe any sensitivity analyses conducted to assess robustness of the synthesized results. | Page 4 |
| Reporting bias assessment | 14 | Describe any methods used to assess risk of bias due to missing results in a synthesis (arising from reporting biases). | Page 3 |
| Certainty assessment | 15 | Describe any methods used to assess certainty (or confidence) in the body of evidence for an outcome. | Page 3 |
| **RESULTS** | | |  |
| Study selection | 16a | Describe the results of the search and selection process, from the number of records identified in the search to the number of studies included in the review, ideally using a flow diagram. | Page 4 and 11 |
|  | 16b | Cite studies that might appear to meet the inclusion criteria, but which were excluded, and explain why they were excluded. | None |
| Study characteristics | 17 | Cite each included study and present its characteristics. | Page 4 |
| Risk of bias in studies | 18 | Present assessments of risk of bias for each included study. | Page 4 |
| Results of individual studies | 19 | For all outcomes, present, for each study: (a) summary statistics for each group (where appropriate) and (b) an effect estimate and its precision (e.g. confidence/credible interval), ideally using structured tables or plots. | Supplemental table 1 |
| Results of syntheses | 20a | For each synthesis, briefly summarise the characteristics and risk of bias among contributing studies. | Page 4-8 |
|  | 20b | Present results of all statistical syntheses conducted. If meta-analysis was done, present for each the summary estimate and its precision (e.g. confidence/credible interval) and measures of statistical heterogeneity. If comparing groups, describe the direction of the effect. | Page 4-8 |
|  | 20c | Present results of all investigations of possible causes of heterogeneity among study results. | None |
|  | 20d | Present results of all sensitivity analyses conducted to assess the robustness of the synthesized results. | Page 8 |
| Reporting biases | 21 | Present assessments of risk of bias due to missing results (arising from reporting biases) for each synthesis assessed. | Supplemental table 2 and 3 |
| Certainty of evidence | 22 | Present assessments of certainty (or confidence) in the body of evidence for each outcome assessed. | Supplemental table 4 |
| **DISCUSSION** | | |  |
| Discussion | 23a | Provide a general interpretation of the results in the context of other evidence. | Page 8 |
|  | 23b | Discuss any limitations of the evidence included in the review. | Page 9 |
|  | 23c | Discuss any limitations of the review processes used. | Page 9 |
|  | 23d | Discuss implications of the results for practice, policy, and future research. | Page 8-9 |
| **OTHER INFORMATION** | | |  |
| Registration and protocol | 24a | Provide registration information for the review, including register name and registration number, or state that the review was not registered. | Page 3 |
|  | 24b | Indicate where the review protocol can be accessed, or state that a protocol was not prepared. | None |
|  | 24c | Describe and explain any amendments to information provided at registration or in the protocol. | None |
| Support | 25 | Describe sources of financial or non-financial support for the review, and the role of the funders or sponsors in the review. | Page 9 |
| Competing interests | 26 | Declare any competing interests of review authors. | Page 9 |
| Availability of data, code and other materials | 27 | Report which of the following are publicly available and where they can be found: template data collection forms; data extracted from included studies; data used for all analyses; analytic code; any other materials used in the review. | None |

**Table 1. Baseline characteristics of the included studies**

| **First author, year** | **Population** | **Time frame** | **Number of**  **centers** | **Study design** | **Type of prediction model study** | **Mean age, y (SD / Range)** | **Sex, man, n (%)** | **Number**  **of intracranial bleed events / sample size** | **Sensitivity (eg. C statistic, AUC)** |
| --- | --- | --- | --- | --- | --- | --- | --- | --- | --- |
|  |  |  |  |  |  |  |  |  |  |
| Barber, 2000 [S1] | Patients with hyperacute ischaemic stroke | 1996.03-1999.05 | Multicen-ter | Prosp-ective cohort study | External validation | 68 (14) | 84 (53.85) | 31 / 156 | Sensitivity of ASPECTS for symptomatic intracerebral haemorrhage was 0.90 and specificity 0.62 |
| Kawano, 2012 [S2] | Patients with anterior circulation ischemic stroke | 2005.10-2008.06 | Single-Center | Retros-pective case-control study | Development | With ICH: 80.1 (9.2); Without ICH: 74.8 (11.8) | With ICH: 11 (58); Without ICH: 77 (55) | 19 / 159 | Sensitivity: 79%, specificity: 60%, AUC: 0.732 |
| Singer, 2009 [S3] | Patients with anterior circulation  stroke | / | Multicen-ter | Retros-pective case-control study | External validation | 69 (59-77) | 119 (55) | 23 / 217 | Sensitivity 0.91; Specificity 0.39; PPV 0.15; NPV 0.97 |
| Espı´nola, 2012 [S4] | Patients with acute ischemic stroke | 2000.11-2008.06 | Multicen-ter | Prosp-ective cohort study | Development | HT: 71.7 (10.9); No HT: 70.5 (12.2) | 188 (21.24) | 189 / 885 | / |
| Menon, 2012 [S5] | Patients with acute ischemic stroke | 2009.01.01-2010.06.30 | Multicen-ter | Retros-pective cohort study | Development | 69.9 (14.7) | 5203 (50.80) | 496 / 10242 | C-statistic in  the derivation sample was 0.71 (95% CI, 0.68- 0.73) and the  Hosmer-Lemeshow test probability value was 0.78 |
| Tanne, 2002 [S6] | Patients with acute ischemic stroke | / | Multicen-ter | Retros-pective case-control study | Development | Symptomatic  ICH: 71 (11); Asymptomatic  ICH: 69 (12); No ICH: 66 (14) | Symptomatic  ICH: 45 (63); Asymptomatic  ICH: 53 (62); No ICH: 577 (55) | 158 / 1205 | The goodness-of-fit statistic (P=0.35) indicated a good fit of the model, and the C statistic was 0.71 |
| Chatterjee, 2017 [S7] | Patients with pulmonary embolism | 2003-2012 | Multicen-ter | Retros-pective cohort study | Development | ICH: 61.0 (51.0-72.0); No ICH: 58.0 (45.0-70.0) | ICH: 39 (45.3); No ICH: 2369 (49.7) | 86 / 4853 | C-statistic in  the derivation sample was 0.65 (0.61-0.70) |
| Lokeskraw-ee, 2017 (JSCD) [S8] | Patients with acute ischemic stroke | 2008.01-2016.09 | Multicen-ter | Retros-pective cohort study | Development | sICH: 66.7 (11.2); asICH: 65.8 (12.4); no ICH: 64.3 (12.2) | sICH: 57 (60.0); asICH: 77 (50.0); no ICH: 497 (53.9) | 249 / 1146 | The AuROC of the SICH score was 0.75 (95% CI，0.71-0.80). By the Hosmer-Lemeshow goodness-of-fit test, the score-predicted percent was calibrated against the observed percent. A P value of 0.480 implied no lack of fit |
| Mazya, 2012 [S9] | Patients with acute ischemic stroke | 2002.12.25-2010.03.01 | Multicen-ter | Prosp-ective cohort study | Development | SICH: 74 (66–78); No SICH: 69 (60-76) | 17904 (57.92) | 568 / 31627 | C-statistic in  the derivation sample was 0.71 |
| Wahlgren, 2008 [S10] | Patients with ischemic stroke | 2002.12-2006.04 | Multicen-ter | Prosp-ective cohort study | Development | ICH: 73; No ICH: 68 | 3877 (60.16) | 107 / 6444 | The mean (95% CI) for  c statistic was 0.674 (0.635 to 0.712) after cross validation |
| Saposnik, 2013 [S11] | Stroke patients | 1991.01-1994.10 | Multicen-ter | Retros-pective cohort study | External validation | SPAN-100 negative: 65.4 (11.1); SPAN-100 positive: 81.0 (5.0) | SPAN-100 negative: 336 (59.8); SPAN-100 positive: 26 (41.9) | SPAN-100 negative: 48 / 562; SPAN-100 positive: 20 / 62 | C-statistic was 0.682 |
| Cappellari, 2018 [S12] | Stroke patients | 2001.05-2016.03 | Multicen-ter | Retros-pective cohort study | Development | 72 (62-78) | 6742 (56.0) | ECASS-II definition: 440 / 12030; SITS-MOST definition: 207 / 12030 | The AUC-ROC of the STARTING-SICH nomogram for predicting the probability of ECASS-II sICH was 0.699 (95% CI, 0.676–0.723) in the training cohort; The Hosmer-Lemeshow goodness-of-fit test comparing predicted and observed rates of sICH showed good calibration of the STARTING-SICH total score (8.058; P=0.327) |
| Flint, 2013 [S13] | Patients with ischemic stroke | / | Multicen-ter | Retros-pective cohort study | External validation | 69.0 (12.6) | 3119 (54.5) | 1784 / 5724 | The THRIVE ROC AUC was 0.64 |
| Flint, 2015 [S14] | Patients with ischemic stroke | / | Multicen-ter | Retros-pective cohort study | Development | 70 (60-76) | 3574 (57.7) | 2911 / 5855 | C-statistic in  the derivation sample was 0.786, 95% CI 0.774-0.798 |
| Asuzu, 2015 (Neurocrit Care) [S15] | Patients with ischemic stroke | 2009.01-2013.07 | Multicen-ter | Prosp-ective cohort study | Development | 70.3 | 102 (48.60) | 40 / 210 | For TURNP, we calculated a Hosmer-Lemeshow χ2 of 7.64 using 10 quantiles, P = 0.47 demonstrating good agreement. TURNP yielded an AUROC of 0.78, 95 % CI (0.64, 0.92); For TURN, we found a Hosmer- Lemeshow χ2 of 5.28 using 10 quantiles, P = 0.73, also demonstrating good agreement. TURN yielded an AUROC of 0.74, 95% CI (0.58, 0.90) |
| Zhou, 2020 [S16] | Patients with acute ischemic stroke | 2014.04-2019.07 | Single-Center | Retros-pective cohort study | Development | ICH: 70 (59-75); Non-ICH: 64 (55-72) | ICH: 23 (69.7); Non-ICH: 134 (67.0) | 33 / 233 | The AUC of the nomogram  for the training sets was 0.828 (0.753-0.903); The Hosmer-Lemesshow text revealed good calibration in the training sets (P=0.509) |
| Erdur, 2018 [S17] | Patients with acute ischemic stroke | 2006-2016 | Single-Center | Prosp-ective cohort study | Development | Non-ICH: 75 (67-83); ICH: 80 (72-87) | Non-ICH: 660 (51.4); ICH: 26 (49.1) | 53 / 1336 | C-statistic was 0.72 (95% CI 0.66-0.79) |
| Kidwell, 2002 [S18] | Patients with acute focal cerebral ischemia within the anterior or posterior circulation | 1992.07-2000.05 | Single-Center | Retros-pective cohort study | Development | 69 (15) | 42 (47) | 35 / 89 | C-statistic was 0.79. The model demonstrated sensitivity 74%, specificity 80%, negative predictive value 83%, positive predictive value PPV 70%, and overall accuracy 78% |
| Soni, 2021 [S19] | Patients with acute ischemic stroke | / | Multicen-ter | Retros-pective cohort study | Development | sICH: 77.6 (9.6); No sICH: 71.4 (14.1) | 484 (54.38) | 56 / 890 | C-statistic was 0.75, 95% CI: 0.65-0.84 |
| Wu, 2020 [S20] | Patients with ischemic stroke | 2016.08-2018.07 | Single-Center | Retros-pective cohort study | Development | 67 (59-74) | 97 (74) | 16 / 131 | C-statistic was 0.9562; The AUC of the nomogram was 0.9562 (95% CI, 0.9221-0.9904, P<0.01) |
| Simoons, 1993 [S21] | Patients with myocardial infarction | 1988-1990 | Multicen-ter | Prosp-ective cohort study | Development | ICH: 65 (8); Control: 58 (11) | ICH: 98 (65); Control: 232 (79) | 150 / 444 | / |
| Guo, 2021 [S22] | Patients with acute ischemic stroke | 2014.04-2020.11 | Multicen-ter | Retros-pective cohort study | Development | All: 66.9; With sICH: 72.2 (11.8);  Without sICH: 66.6 (12.5) | All: 756(63); With sICH: 37 (56.1); Without sICH: 719 (63.4) | 66 / 1200 | The AUCROC of the nomogram was 0.788 (95% CI, 0.737-0.840) |
| Larrue, 2001 [S23] | Patients with ischemic stroke | / | Multicen-ter | Prosp-ective cohort study | / | / | / | Placebo Group: 12 / 386;  rtPA Group: 48 / 407 | / |
| Nawar, 2019 [S24] | Stroke patients | 2011.01-2017.08 | Single-Center | Retros-pective cohort study | / | No ICH  74 (62-83); ICH  80 (70-87) | No ICH: 130 (52.2); ICH: 26 (50.0) | 52 / 301 | AUC, 0.80; 95% CI 0.74-0.86, (P=0.012) |
| Kufner, 2012 [S25] | Patients with acute stroke | 2008.05-2010.05 | Multicen-ter | Retros-pective cohort study | / | 71.3 (12.5) | 54 (49.5) | 17 / 109 | / |
| Qiu, 2017 [S26] | Patients with acute ischemic stroke | 2015.01-2016.06 | Single-Center | Retros-pective cohort study | / | 65.36 (10.02) | 106 (66.67) | 6 / 159 | The cut-off values of fT3 for sICH was 3.54 pg/mL (sensitivity 83%; specificity 83%; area under the curve 0.88) |
| Rocco, 2013 [S27] | Patients with acute stroke | 1998.03-2011.11 | Single-Center | Retros-pective cohort study | / | 74 (65-80) | 583 (52.5) | 222 / 1112 | The corresponding c-statistic was 0.65 for NIHSS score (CI, 0.60-0.71; P<0.001) and 0.80 for HbA1c (CI, 0.75-0.80; P<0.001) to predict sICH per NINDS definition |
| Lee, 2019 [S28] | Patients with acute stroke | 2008.02-2018.01 | Single-Center | Retros-pective cohort study | / | Non-sICH: 70 (60-77); sICH: 74 (60-76) | 233 (62.13) | 42 / 375 | SBP and platelet count showed poor predictability (AUC, 0.661; 95% CI, 0.510 to 0.811; AUC, 0.684; 95% CI, 0.503 to 0.865, respectively) for sICH. NIHSS score showed fair predictability for sICH (AUC, 0.780; 95% CI, 0.713 to 0.848) |
| Puig, 2017 [S29] | Patients with acute stroke and patients with middle cerebral artery occlusion | 2012.10-2016.06 | Single-Center | Prosp-ective cohort study | / | Without PH-2: 75 (66-81.25); With PH-2: 78.5 (69.75-81.25) | 78 (50) | 37 / 156 | The negative predictive value of HPrs-PCT at a threshold of 7mL/100g/min was 0.84 for HT and 0.93 for PH-2. The multiple regression analysis selected HPrs-PCT at 7mL/100g/min combined with platelets and baseline NIHSS score as the best model for predicting HT (AUC 0.77) |
| Dharmasar-oja, 2012 [S30] | Patients with acute ischemic stroke | 2007.06-2010.03 | Single-Center | Prosp-ective cohort study | / | 64 (13) | 115 (59.90) | 36 / 192 | These values were 12 seconds for the prothrombin time, with 70% sensitivity and 73% specificity (area under curve = 0.742), and 1.0 for the INR, with 73% sensitivity and 68% specificity (area under curve = 0.721). At an NIHSS level of 15, 84% sensitivity and 66% specificity (area under curve = 0.739) was noted in detecting asymptomatic ICH. A blood glucose level of 8.325 mmol/L, with 63% sensitivity and 78% specificity (area under curve = 0.656) |
| Lin, 2018 (J Atheroscler Thromb) [S31] | Patients with acute ischemic stroke | 2004.12-2016.12 | Multicen-ter | Prosp-ective cohort study | / | Hyperlipidemia: 67.1 (12.3); No hyperlipidemia: 69.8 (13.3) | 1179 (63.90) | Hyperlipide-mia: 93 / 752; No hyperlipidem-ia: 171 / 1093 | / |
| Nisar, 2019 [S32] | Stroke patients | 2014.07.01-2018.03.31 | Single-Center | Retros-pective cohort study | / | 63.4 (14.42) | 48 (53.93) | 5 / 89 | AUROC (HAT):  0.769 (95% CI, 0.58-0.96; P=0.044) |
| Asuzu, 2015 (Neurocrit Care 2) [S33] | Patients with acute ischemic stroke | 2009.01-2013.12 | Single-Center | Retros-pective cohort study | / | / | / | 12 / 210 | Area under ROC curve 0.66, 95% CI 0.48-0.83. Odds ratio 2.72, 95% CI 1.12-6.61, P = 0.03 |
| Khatri, 2010 [S34] | Patients with acute ischemic stroke | 1999.05-2005.11 | Multicen-ter | Retros-pective cohort study | / | 63.96 (14.2) | 52 (37.7) | 38 / 77 | / |
| Lansberg, 2007 [S35] | Patients with acute stroke | / | Multicen-ter | Prosp-ective cohort study | / | SICH:  78 (12); No SICH:  70 (15) | 41 (55.41) | 7 / 74 | / |
| Lokeskrawee, 2017 (CNR) [S36] | Patients with acute ischemic stroke | 2008.01-2016.09 | Multicen-ter | Retros-pective cohort study | / | sICH: 66.7 (11.2); asICH: 65.8 (12.4); no ICH: 64.3 (12.2) | 631 (53.84) | 249 / 1172 | / |
| Tomáš, 2018 [S37] | Patients with ischemic stroke | 2005.07-2017.01 | Single-Center | Retros-pective cohort study | / | 68.0 (12.3) | 103 (65.19) | 11 / 158 | / |
| Nagaraja, 2018 [S38] | Patients with acute ischemic stroke | 2009.01.01-2013.12.31 | Single-Center | Retros-pective cohort study | / | 67 (15) | 197 (54) | 87 / 366 | / |
| Ribo, 2004 [S39] | Stroke patients with middle cerebral artery occlusion | 2002.02-2003.10 | Single-Center | Prosp-ective cohort study | / | 70 (12) | 46 (60.5) | 6 / 77 | Sensibility 75%, specificity 97.6% (P＜0.01), positive predictive value 75%, negative predictive value 97.6% |
| Selim, 2002 [S40] | Patients with ischemic stroke | 1997.07-2001.05 | Multicen-ter | Retros-pective cohort study | / | 73 (16) | 14 (48.3) | 17 / 29 | / |
| Pjevic, 2021 [S41] | Patients with acute ischemic stroke | 2016.08-2018.08 | Single-Center | Retros-pective cohort study | / | 72.05 (10.10) | 88 (53.0) | 30 / 166 | / |
| Shinoda, 2017 [S42] | Patients with major artery occlusion | 2012.04- 2016.07 | Single-Center | Retros-pective cohort study | / | 77.6 (10.4) | 51 (63.75) | 25 / 80 | / |
| Kimura, 2008 [S43] | Patients with acute ischemic stroke | 2005.10-2007.04 | Single-Center | Prosp-ective cohort study | / | 73.2 (10.7) | 21 (51.22) | 19 / 41 | / |
| Cho, 2013 [S44] | Patients with acute ischemic stroke | 2010.06-2012.02 | Single-Center | Retros-pective cohort study | / | 69.6 (12.32) | 114 (74.03) | 51 / 154 | A UACR of 30 mg albumin/g creatinine predicted HT with a moderate sensitivity (72.5%) and a moderate specificity (55.3%); AUC 0.659; 95% CI, 0.569-0.750; P = 0.001 |
| Chen, 2018 [S45] | Patients with acute ischemic stroke | 2009.05-2017.01 | Single-Center | Retros-pective cohort study | / | 67.12 (12.76) | 326 (64.8) | 17 / 503 | / |
| Brekenfeld, 2007 [S46] | Patients with acute ischemic stroke | 1992.12-2004.03 | Single-Center | Retros-pective cohort study | / | 60 (13) | 161 (54.76) | 14 / 294 | / |
| Das, 2020 [S47] | Patients with acute ischemic stroke | 2017.02-2019.09 | Single-Center | Prosp-ective cohort study | / | sICH: 59.9 (5.5); Without sICH: 56.1 (4.5) | 34 (56.67) | 8 / 60 | / |
| Zou, 2013 [S48] | Patients with acute ischemic stroke | 2007.10-2011.12 | Single-Center | Retros-pective cohort study | / | 71 (26-91) | 95 (52.2) | 49 / 182 | / |
| Gensicke, 2016 [S49] | Stroke patients | Up to 2013.12.31 | Multicen-ter | Prosp-ective cohort study | / | Proteinuria: 73 (64-79);  No proteinuria: 68 (58–76) | 1509 (44.41) | 356 / 3398 | / |
| Yang, 2020 [S50] | Cardioembolism (CE) and large artery atherosclerosis stroke (LAA) patients | 2015.01-2019.01 | Single-Center | Retros-pective case-control study | / | 63.2 (12.4) | 177 (71.66) | 62 / 247 | / |
| Luo, 2020 [S51] | Patients with ischemic stroke | 2015.04-2018.12 | Single-Center | Retros-pective cohort study | / | 63 (54-71) | 552 (72.3) | 78 / 763 | TC/HDL-C: AUC 0.580 (0.513-0.646), P=0.021. Optimum Cuff-Point 4.05. Sensitivity 73.1%, specificity 43.6%; TG/HDL-C: AUC 0.599 (0.532-0.666), P=0.004. Optimum Cuff-Point 0.82. Sensitivity 50%, specificity 68.9%; LDL-C/HDL-C: AUC 0.581 (0.517-0.645), P=0.018. Optimum Cuff-Point 2.67. Sensitivity 79.49%, specificity 37.08% |
| Castellanos, 2018 [S52] | Patients with acute ischemic stroke | 2012.08-2015.02 | Single-Center | Prosp-ective cohort study | / | 72 (13) | 67 (50.4) | 7 / 133 | Caveolin-1 levels≤0.17 ng/mL had the highest predictive capacity of sHT (86% sensitivity, 65% specificity, 99% negative predictive value, 12% positive predictive value) |
| Liu, 2016 [S53] | Patients with acute ischemic stroke | 2013.01-2015.10 | Single-Center | Retros-pective cohort study | / | 63.6 (13.9) | 26 (56.53) | 11 / 46 | / |
| Acampa, 2017 [S54] | Patients with ischemic stroke | / | Single-Center | Prosp-ective cohort study | / | 73 (12) | 135 (52.33) | 55 / 258 | / |
| Fàbregas, 2006 [S55] | Patients with ischemic stroke | 1999-2004 | Multicen-ter | Retros-pective cohort study | / | 68 (10.9) | 194 (56) | 40 / 347 | / |
| Huynh, 2004 [S56] | Patients with acute myocardial infarction | 1998.01.01-2000.12.31 | Multicen-ter | Prosp-ective cohort study | / | 64.0 (13.0) | 9087 (71.1) | 82 / 12739 | / |
| Lin, 2018 (Journal of the Neurological Sciences) [S57] | Patients with acute ischemic stroke | 2004.12.01-2016.12.31 | Multicen-ter | Prosp-ective cohort study | / | Blood glucose ≥ 200:  71.0 (59.0-79.0);  Blood glucose < 200: 69.0 (61.0-77.0) | 1273 (63.18) | By SITS-MOST: 52 / 2015; By NINDS: 88 / 2015 | SITS-MOST: AUC 0.6151, P=0.0584; NINDS: AUC 0.5721, P=0.1165 |
| Mundiyanapurath, 2018 [S58] | Patients with ischemic stroke | 2002.12-2013.02 | Multicen-ter | Retros-pective cohort study | / | 71 (61-78) | 31825 (56.57) | 1037 / 56258 | / |
| Mendioroz, 2009 [S59] | Stroke patients | / | Single-Center | Prosp-ective cohort study | / | 71.8 (11.4) | 69 (59) | 13 / 117 | / |
| Cho, 2008 [S60] | Patients with acute ischemic stroke | 2004.03-2006.10 | Single-Center | Retros-pective cohort study | / | 62.6 (11.6) | 52 (59.09) | SICH-1: 6 / 88; SICH-2: 5 / 88 | / |
| Lin, 2020 [S61] | Patients with acute ischemic stroke | 2008.03-2017.11 | Multicen-ter | Retros-pective cohort study | / | Early ICH: 66.9 (9.7); Late ICH: 65.3 (12.1); No ICH: 67.0 (13.0) | 133 (67.51) | 38 / 197 | / |
| Kamal, 2021 [S62] | Patients with acute stroke | / | Multicen-ter | Retros-pective cohort study | / | sICH:  74.1 (14.0); No sICH: 71.2 (14.9) | 409 (51.51) | 51 / 794 | / |
| Piccardi, 2015 [S63] | Patients with ischemic stroke | 2008-2011 | Multicen-ter | Retros-pective cohort study | / | 68.9 (12.1) | 190 (58) | 40 / 327 | / |
| Cucchiara, 2009 [S64] | Patients with acute ischemic stroke | 2003.05-2006.06 | Multicen-ter | Retros-pective cohort study | / | 68 (13) | 550 (57) | 54 / 965 | / |
| Dzwigal, 2013 [S65] | Stroke patients | 2006.09-2011.03 | Single-Center | Retros-pective cohort study | / | 69.07 (10.57) | 112 (56) | 35 / 200 | / |
| Yu, 2020 [S66] | Patients with acute non cardiogenic anterior circulation ischemic stroke | 2015.07.01-2018.06.30 | Single-Center | Retros-pective case-control study | / | 61 (53-67) | 181 (74.8) | 31 / 242 | / |
| Selker, 1994 [S67] | Patients with acute myocardial infarction | / | Multicen-ter | Retros-pective cohort study | / | Patients: 64 (8.2); Control Subjects: 57 (10) | Patients: 13 (68); Control Subjects: 138 (79) | 19 / 194 | Receiver-operating characteristic curve area, 0.82 versus 0.77 with systolic pressure and age, 0.75 with mean arterial pressure, 0.71 with diastolic pressure, and 0.81 with both systolic and diastolic pressures |
| Curtze, 2015 [S68] | Patients with ischemic stroke | 2001.12-2014.02 | Single-Center | Retros-pective cohort study | / | No sICH: 69 (60-77); sICH: 73 (64-79) | 1407 (56.71) | 124 / 2481 | For a Blennow score of >4, we got a sensitivity of 19%, a specificity of 91%, and a positive predictive value of 10% |
| Qiu, 2021 [S69] | Stroke patients | 2017.06-2020.01 | Single-Center | Prosp-ective cohort study | / | 67.0 (11.6) | 114 (65.1) | 41 / 175 | / |
| Haefelin, 2006 [S70] | Patients with acute stroke | / | Multicen-ter | Retros-pective case-control study | / | 61 (14) | / | 25 / 449 | / |
| Willer, 2015 [S71] | Patients with ischemic stroke | 2009.04-2012.07 | Single-Center | Retros-pective case-control study | / | LA: 75.8; No LA: 63.6 | / | 23 / 311 | / |
| Tsetsou, 2016 [S72] | Patients with acute ischemic stroke | 2003.01-2013.12 | Single-Center | Retros-pective cohort study | / | 69.8 (17.9) | 101 (53.16) | 24 / 190 | / |
| Xu, 2017 [S73] | Patients with acute stroke | 2013.02-2015.03 | Single-Center | Retros-pective cohort study | / | 65.6 (10.6) | 103 (63.6) | 20 / 162 | / |
| Tong, 2014 [S74] | Patients with acute ischemic stroke | 2008.01-2012.09 | Multicen-ter | Retros-pective cohort study | / | 71 (18-103) | 3563 (49.5) | 323 / 7193 | / |
| Brass, 2000 [S75] | Elderly patients with acute myocardial infarction | 1994-1995 | Multicen-ter | Retros-pective cohort study | / | 73.4 (median: 73) | 18054 (56.9) | 455 / 31732 | / |
| Derex, 2005 [S76] | Patients with symptoms of acute internal carotid artery (ICA) territory stroke | 2001.03-2002.10 | Single-Center | Prosp-ective cohort study | / | 63.8 (13.6) | 25 (52.08) | 21 / 48 | ROAUC 0.82 |
| Sun, 2020 [S77] | Patients with acute ischemic stroke | 2014.01-2019.06 | Single-Center | Prosp-ective cohort study | / | Hemorrhage group: 68.92 (7.41); Non-hemorrhage group: 65.42 (6.56) | 212 (69.28) | 52 / 306 | OR 7.317, 95% CI (3.259-10.662), P=0.001; AUC 0.954 |
| Xing, 2014 [S78] | Patient with acute cerebral ischemia | 2004.03.01-2011.11.30 | Single-Center | Retros-pective cohort study | / | HT group: 60.05 (10.91); Non-HT group: 58.21 (10.62) | 154 (71.30) | 41 / 216 | / |
| JAEGERE, 1992 [S79] | Patients with acute myocardial infarction | Since October 1988 and lasted 18 months | Multicen-ter | Prosp-ective case-control study | / | ICH: 65 (8); Control: 59 (8) | 74 (79.57) | 31 / 93 | / |
| Tütüncü, 2013 [S80] | Patients with ischemic stroke | 2005.01-2012.08 | Single-Center | Retros-pective cohort study | / | 75 (66-83) | 391 (53) | 34 / 740 | / |
| Jucevičiūtė, 2019 [S81] | Patients with acute ischemic stroke | 2014.01-2018.04 | Single-Center | Retros-pective cohort study | / | 77 (70-84) | 81 (40.3) | 23 / 201 | Sensibility 69.6%; Specificity 60.7%; AUC 0.643 (95% CI 0.526-0.759, P=0.026) |
| Cougo-Pinto, 2012 [S82] | Patients with acute ischemic stroke | 2001.05-2010.04 | Single-Center | Retros-pective cohort study | / | 63 (12.8) | 59 (52.21) | 7 / 113 | / |
| Muengtaweepongsa, 2017 [S83] | Patients with acute ischemic stroke | 2010.01-2012.07 | Single-Center | Retros-pective cohort study | / | No ICH: 69; sICH: 72; AsICH: 71 | 185 (58.92) | 46 / 314 | / |
| Shin, 2017 [S84] | Patients with hyperacute stroke | / | Single-Center | Prosp-ective cohort study | / | 62.3 | 83 (64.84) | 41 / 128 | / |
| Kandzari, 2004 [S85] | Patients with acute myocardial infarction | / | Multicen-ter | Prosp-ective case-control study | / | ICH: 70.3 (63.1-76.7); Controls: 70.1 (62.1-76.1) | ICH: 161 (60); Controls: 384 (70) | 268 / 41021 | / |
| Wang, 2019 [S86] | Patients with acute stroke | 2013.02-2018.01 | Single-Center | Retros-pective cohort study | / | 67.01 (31.88) | 267 (66.3) | 46 / 403 | / |
| Szegedi, 2019 [S87] | Patients with acute ischemic stroke | 2011.03-2012.12 | Single-Center | Retros-pective cohort study | / | 5G/5G: 69.9 (13.6); 4G/5G and 4G/4G: 68.8 (11.8) | 79 (60.31) | 13 / 131 | / |
| Sun, 2015 [S88] | Stroke patients | 2006.01.01-2008.04.18 | Single-Center | Retros-pective cohort study | / | 63 (11.4) | 36 (50) | 17 / 72 | / |
| Vandelli, 2015 [S89] | Patients with ischemic stroke | 2010.01.01-2011.01.01 | Multicen-ter | Retros-pective case-control study | / | 66.6 (13.5) | 66 (63.5) | 24 / 104 | / |
| Christoforid-is, 2009 [S90] | Patients with acute ischemic stroke | 1995.05-2007.04 | Single-Center | Retros-pective cohort study | / | 68.0 (33-90) | 56 (53.85) | 26 / 104 | / |
| Liu, 2017 [S91] | Chinese patients with acute ischemic stroke | 2007.05-2012.04 | Multicen-ter | Retros-pective cohort study | / | (1) mSITS-MOST: sICH 66.22 (8.89); Non-sICH 63.43 (11.38) (2) ECASS II: sICH 66.73 (9.04); Non-sICH 63.35 (11.41) (3) NINDS: sICH 67.18 (9.28); Non-sICH 63.27 (11.42) | 688 (60.99) | mSITS-MOST: 23 / 1128; ECASSⅡ: 44 / 1128; NINDS: 61 / 1128 | / |
| Foerch, 2007 [S92] | Patients with acute stroke | 2003.03-2005.07; 2002. 07-2005.06; 2002.10-2004.12 | Multicen-ter | Retros-pective cohort study | / | 69 (13) | 149 (54) | 80 / 275 | A pretreatment S100B value above 0.23 ug/L had only a moderate sensitivity (0.46) and specificity (0.82) for predicting severe parenchymal bleeding (parenchymal hemorrhage 2) |
| Marsh, 2013 [S93] | Patients with acute ischemic stroke | 2004.01-2011.10 | Multicen-ter | Retros-pective cohort study | / | 66.8 (23-95) | 110 (49) | 14 / 224 | / |
| Castellanos, 2004 [S94] | Patients with acute ischemic stroke | 1999.10-2001.07 | Multicen-ter | Prosp-ective cohort study | / | 67 (12) | 48 (55.17) | 26 / 87 | The sensitivity, specificity, and positive and negative predictive values of plasma c-Fn ≥ 3.6 ug/mL for the prediction of HI-2 and PH were 100%, 96%, 44%, and 100%, respectively |
| Che, 2017 [S95] | Patients with ischemic stroke | 2013.01.01-2016.10.31 | Single-Center | Prosp-ective cohort study | / | 61 (27-88) | 316 (73.83) | 24 / 428 | / |
| Yao, 2016 [S96] | Patients with acute ischemic stroke | 2011.01-2015.01 | Single-Center | Retros-pective cohort study | / | 62.2 (12.3) | 118 (60.82) | 21 / 194 | ROAUC 0.685 (95% CI: 0.603-0.768). The ROC showed that Hcy level was a moderately sensitive and specific index to predict the prognosis with an optimal cut-off value at 19.95 µmol/L (sensitivity [58.2%], specificity [80.3%]) |
| Gnofam, 2013 [S97] | Non diabetic stroke patients | 2003.09.30-2012.10.29 | Single-Center | Prosp-ective cohort study | / | 71 (57-81) | 242 (47.9) | 37 / 505 | / |
| González, 2012 [S98] | Patients with ischemic stroke | / | Single-Center | Prosp-ective cohort study | / | 68.2 (11.7) | 67 (51.9) | 47 / 129 | PDGF-CC levels ≥ 175 ng/mL at 24 h predict the development of PH with a sensitivity of 90% and specificity of 88% (area under the curve 0.936; P < 0.0001) |
| Tsivgoulis, 2009 [S99] | Patients with acute ischemic stroke | 1996.01-2005.12 | Single-Center | Retros-pective cohort study | / | 65 (15) | 282 (55.29) | 31 / 510 | / |
| Palumbo, 2007 [S100] | Patients with acute stroke | 1999-2001 | Multicen-ter | Prosp-ective cohort study | / | VSS＞4: 78.3;  VSS≤4: 69.2 | 351 (42.80) | 29 / 820 | / |
| James, 2018 [S101] | Patients with ischemic stroke | 2015.03.01-2017.03.01 | Single-Center | Retros-pective cohort study | / | No sICH: 70.8 (15.4); sICH: 80.2 (15.8) | 49 (45.37) | 19 / 108 | / |
| Li, 2017 [S102] | Patients with acute stroke | 2011.01-2014.01 | Multicen-ter | Retros-pective cohort study | / | 65.3 (13.9) | 59 (55.7) | 21 / 106 | / |
| Zhu, 2021 [S103] | Patients with acute ischemic stroke | 2017.06-2018.02 | Multicen-ter | Retros-pective cohort study | / | / | / | 48 / 109 | The joint detection of Apelin and Vaspin showed a sensitivity of 77.08% and a specificity of 73.77% for forecasting HT in CIS patients after thrombolytic therapy (all P < 0.001) |
| Guo, 2016 [S104] | Stroke patients | 2012.03-2015.08 | Multicen-ter | Prosp-ective cohort study | / | PH: 70.1 (10.5); SICH: 68.7 (11.7) | 123 (65.08) | PH: 28 / 189; SICH: 17 / 189 | The areas under the curve (AUCs) for the ability of the NLR to predict PH or sICH were 0.833 with 78.6% sensitivity and 79.5% specificity and 0.814 with 76.5% sensitivity and 75.6% specificity, respectively |
| Lakhter, 2020 [S105] | Patients with deep venous thrombosis | 2005.01-2013.12 | Multicen-ter | Retros-pective cohort study | / | Intracranial  Hemorrhage: 65.2 (13.8);  No Intracranial  Hemorrhage: 53.7 (17.1) | 3646 (51.22) | 51 / 7119 | / |

**Table 1. (continued)**

| **First author, year** | **Variables in multivariate logistic regression and measures of association (OR/RR/HR, 95%CI and P value)** | | | | | | | | | | | | |
| --- | --- | --- | --- | --- | --- | --- | --- | --- | --- | --- | --- | --- | --- |
|  | **Item 1** | **Item 2** | **Item 3** | **Item 4** | **Item 5** | **Item 6** | **Item 7** | **Item 8** | **Item 9** | **Item 10** | **Item 11** | **Item 12** | **Item 13** |
| Barber, 2000 [S1] | Baseline ASPECT (OR 14; 95% CI 2-117; P=0.012) | Serum glucose (≤10 vs >10 mmol/L) (OR 4.9; 95% CI 1-21; P=0.032) |  |  |  |  |  |  |  |  |  |  |  |
| Kawano, 2012 [S2] | Lower ASPECTS+W＜8 (OR  0.75; 95% CI 0.58-0.96; P=0.027) | Administ-ration of  IV-tPA (OR 9.13; 95% CI 2.15-46.21; P=0.004) |  |  |  |  |  |  |  |  |  |  |  |
| Singer, 2009 [S3] | DWI-Aspects score (OR 0.791; 95% CI 0.659-0.949; P=0.012) |  |  |  |  |  |  |  |  |  |  |  |  |
| Espı´nola, 2012 [S4] | Baseline NIHSS (OR 1.054; 95% CI 1.019-1.091; P=0.002) | AF (OR 1.705; 95% CI 1.158-2.512; P=0.007) | DBP, 10mm (OR 1.161; 95% CI 1.033-1.305; P=0.012) | OTT, 10min (OR 1.039; 95% CI 1.008-1.072; P=0.014) | Rs1801020, C allele (OR 2.036; 95% CI 1.378-3.010; P＜0.001) | Rs669, A allele (OR 2.190; 95% CI 1.569- 3.056; P＜0.001) |  |  |  |  |  |  |  |
| Menon, 2012 [S5] | Age per 10 years (OR 1.26; 95% CI 1.17-1.35; P＜0.001) | NIHSS per 1 unit up to  maximum 20  (OR 1.09; 95% CI 1.08-1.12; P＜ 0.001) | Systolic BP per 10 mm Hg  up to maximum 180  (OR 1.12; 95% CI 1.07-1.17; P＜0.001) | Blood glucose per 10 mg/dL  up to maximum 150  (OR 1.06; 95% CI 1.02-1.11; P=0.003) | Female versus male (OR 0.71; 95% CI 0.59-0.87; P=0.001) | Asian versus non-Asian (OR 2.12; 95% CI 1.28-3.50; P=0.004) |  |  |  |  |  |  |  |
| Tanne, 2002 [S6] | NIHSS (per 1 category increase) (OR 1.31; 95% CI 1.04-1.64) | Glucose (per 50 mg/dL [2.78 mmol/L] increase) (OR 1.36; 95% CI 1.11-1.67) | Platelets (per 50 000/mm^3^ increase) (OR 0.76; 95% CI 0.61–0.92) | Early CT changes＜33% of MCA (OR 2.90; 95% CI 1.64-5.13);  Early CT changes＞33% of MCA (OR 3.24; 95% CI 1.38-7.61) |  |  |  |  |  |  |  |  |  |
| Chatterjee, 2017 [S7] | Peripheral vascular disease (OR 1.59; 95% CI 1.12-2.90; P=0.049) | Age>65 (Elderly) (OR 1.99; 95% CI 2.01-1.97; P=0.007) | Prior CVA (OR 30.90; 95% CI 27.21-36.5; P<0.001) | Prior myocardi-al infarction (OR 1.80; 95% CI 1.33-1.99; P=0.046) |  |  |  |  |  |  |  |  |  |
| Lokeskrawee, 2017 (JSCD) [S8] | Valvular heart diseases (OR 2.09; 95% CI 1.07-4.10; P=0.031) | Use of aspirin (OR 1.90; 95% CI 1.34-2.68; P＜0.001) | SBP prior to thrombol-ysis ≥140 mmHg (OR 1.50; 95% CI 1.09-2.08; P=0.014) | NIHSS 10-20 (versus <10) (OR 2.15; 95% CI 1.52-3.04; P＜0.001); NIHSS >20 (versus <10) (OR 3.77; 95% CI 2.36-6.02; P＜0.001) | Platelet count <250 (×10^3^ cell/mm^3^) (OR 1.53; 95% CI 1.11-2.12; P=0.010) | IV anti-hypertens-ive drugs during thrombol-ysis (OR 1.66; 95% CI 1.12-2.46; P=0.012) |  |  |  |  |  |  |  |
| Mazya, 2012 [S9] | NIHSS ≥13 (OR 2.2; 95% CI 1.7-3.0; P<0.001); NIHSS 7-12 (OR 1.6; 95% CI 1.1-2.1; P=0.006) | Blood sugar (OR 2.1; 95% CI 1.7-2.6; P＜0.001) | SBP (OR 1.6; 95% CI 1.3-2.0; P＜0.001) | Age (OR 1.7; 95% CI 1.4-2.0; P＜0.001) | Body weight (OR 1.6; 95% CI 1.2-2.0; P＜0.001) | Onset-to-treatment time≥180 min (OR 1.5; 95% CI 1.2-2.0; P=0.002) | Aspirin monother-apy (OR 1.8; 95% CI 1.5-2.1; P＜0.001) | Aspirin+clopidogrel (OR 3.2; 95% CI 1.9-5.2; P＜0.001) | History of hypertension (OR 1.4; 95% CI 1.1-1.7; P=0.004) |  |  |  |  |
| Wahlgren, 2008 [S10] | NIHSS 8-14 (OR 2.61; 95% CI 1.34–5.07); NIHSS 15-18 (OR 2.98; 95% CI 1.48-6.03) | Stroke (OR 1.73; 95% CI 1.02-2.93) | Aspirin (OR 1.58; 95% CI 1.04-2.39) | Age (OR 1.57; 95% CI 1.18-2.10) | SBP (OR 1.33; 95% CI 1.06-1.65) | Body weight (OR 1.32; 95% CI 1.09-1.60) | Blood sugar (OR 1.24; 95% CI 1.06-1.45) |  |  |  |  |  |  |
| Saposnik, 2013 [S11] | Age +NIHSS (OR 4.93; 95% CI 2.64-9.16) |  |  |  |  |  |  |  |  |  |  |  |  |
| Cappellari, 2018 [S12] | SBP (OR 1.009; 95% CI 1.004-1.014; P=0.001) | Age (OR 1.019; 95% CI 1.009-1.028; P<0.001) | OTT (OR 1.002; 95% CI 1.001-1.005; P=0.019) | NIHSS (OR 1.050; 95% CI 1.034-1.066; P<0.001) | Blood sugar (OR 1.006; 95% CI 1.004-1.008; P<0.001) | Aspirin alone (OR 1.537; 95% CI 1.251-1.889; P<0.001) | Aspirin plus clopidogr-el (OR 2.232; 95% CI，1.264-3.941; P=0.006) | Oral  anticoagulant with INR≤1.7 (OR 2.065; 95% CI 1.302-3.274; P=0.002) | Current infarction sign (OR 1.444; 95% CI 1.302-3.274; P=0.012) | Hyperden-se artery sign (OR 1.390; 95% CI 1.118-1.730; P=0.003) |  |  |  |
| Flint, 2013 [S13] | Age (OR 0.51; 95% CI 0.46-0.56; P<0.001) | NIHSS (OR 0.23; 95% CI 0.21-0.26; P<0.001) | Chronic  disease scale (Hypertension, Diabetes, and AF) (OR 0.77; 95% CI 0.71-0.84; P<0.001) |  |  |  |  |  |  |  |  |  |  |
| Flint, 2015 [S14] | Age (OR 0.965; 95% CI 0.960-0.970; P<0.001) | NIHSS (OR 0.825; 95% CI 0.815-0.836; P<0.001) | Hypertension (OR 0.876; 95% CI 0.751-1.021; P<0.001) | Diabetes (OR 0.651; 95% CI 0.547-0.775; P<0.001) | AF (OR 0.463; 95% CI 0.334-0.642; P<0.001) |  |  |  |  |  |  |  |  |
| Asuzu, 2015 (Neurocrit Care) [S15] | mRS before stroke (OR 1.54; 95% CI 1.09-2.18; P=0.02) | Baseline NIHSS (OR 1.13; 95% CI 1.05-1.22; P=0.002) | Platelet count (OR 0.99; 95% CI 0.98-1.00; P=0.04) |  |  |  |  |  |  |  |  |  |  |
| Zhou, 2020 [S16] | AF (OR 4.92; 95% CI 2.09-11.57; P<0.001) | NIHSS (OR 1.11; 95% CI 1.04-1.18; P=0.002) | Glucose (OR 1.27; 95% CI 1.08-1.50; P=0.004) |  |  |  |  |  |  |  |  |  |  |
| Erdur, 2018 [S17] | Age>80 years (OR 1.8; 95% CI 1.03-3.3; P=0.04) | National Institutes of Health Stroke Scale≥5  (OR 2.4; 95% CI 1.1–5.0; P=0.02) | Glucose >125 mg/dL (OR 2.5; 95% CI 1.4-4.5; P=0.003) | Systolic blood pressure >155 mmHg (OR 2.0; 95% CI 1.1-3.3; P=0.02) | Pretreatment with medium or high­dose  statins  (OR 2.6; 95% CI 1.3–5.1; P=0.005) |  |  |  |  |  |  |  |  |
| Kidwell, 2002 [S18] | NIHSS (OR 1.148; 95% CI 0.933-1.211; P=0.001) | Time to recanalize-tion (OR 1.004; 95% CI 0.996-1.006; P=0.117) | Platelet count (OR 0.994; 95% CI 0.986-1.010; P=0.089) | Glucose (OR 1.007; 95% CI 0.995-1.012; P=0.216) |  |  |  |  |  |  |  |  |  |
| Soni, 2021 [S19] | Age > 75 years (OR 4.5; 95% CI 2.4-8.7; P<0.001) | Pulse pressure > 110 mmHg (OR 4.5; 95% CI 1.4-14.2; P=0.0049) | NIHSS > 10 (OR 2.9; 95% CI 1.7-5.3; P< 0.001) | Taking Antithrombotic (OR 1.8; 95% CI 1.0-3.2; P=0.0318) | History of Hypertens-ion (OR 2.4; 95% CI 1.2-4.6; P=0.0261) | History of Hyperlipidaemia (OR 2.2; 95% CI 1.3-3.9; P=0.0046) | AF (OR 1.6; 95% CI 0.9-2.7; P=0.0022) | SBP>212mmHg (OR 10.3; 95% CI 1.7-62.7; P=0.0019); SBP 150-212 mmHg (OR 1.9; 95% CI 1.1-3.3; P=0.0210) | Blood glucose level before treatment >8.9 mmoL/L (OR 2.7; 95% CI 1.5-4.8; P<0.001) | Age > 80 years &NIHSS>7 (OR 3.5; 95% CI 2.0-6.1; P<0.001) | Age > 85 years & History of Hypertension (OR 2.1; 95% CI 1.0-4.3; P=0.0429) | Age > 85 years & Diastolic blood pressure > 110 mmHg (OR 6.1; 95% CI 2.3-16.4; P<0.001) | Age > 85 years & Weight > 73 kg (OR 2.4; 95% CI 0.9-6.8; P=0.0500) |
| Wu, 2020 [S20] | Chronic disease scale (hypertension, diabetes, atrial  fibrillation) (OR 4.46; 95% CI 1.87-13.09; P=0.002) | Cerebral small vascular diseases (OR 2.94; 95% CI 1.47-6.78; P=0.004) | NIHSS ≥13 (OR 24.53; 95% CI 4.54-204.38; P=0.001) | OTT≥ 180 (OR 7.77; 95% CI 1.50-61.93; P=0.026) |  |  |  |  |  |  |  |  |  |
| Simoons, 1993 [S21] | Age > 65 year (OR 2.2; 95% CI 1.4-3.5) | Weight < 70 kg (OR 2.1; 95% CI 1.3-3.2) | Hypertens-ion on admission (OR 2.0; 95% CI 1.2-3.2) | AIteptase (OR 1.6; 95% CI 1.0-2.5) |  |  |  |  |  |  |  |  |  |
| Guo, 2021 [S22] | AF (OR 3.25; 95% CI 1.89-5.60; P<0.001) | Blood sugar (OR 1.13; 95% CI 1.07-1.20; P<0.001) | NLR (OR 1.05; 95% CI 1.01-1.09; P=0.024) | NIHSS (OR 1.07; 95% CI 1.04-1.10; P<0.001) |  |  |  |  |  |  |  |  |  |
| Larrue, 2001 [S23] | rt-PA (OR 3.61; 95% CI 1.78-7.31; P<0.0001) | Attenuati-on of density on baseline CT (OR 2.64; 95% CI 1.59-4.39; P<0.001) | Prior congestive heart failure (OR 2.57; 95% CI 1.16-5.71; P=0.02) | Age (OR 1.04; 95% CI 1.00-1.08; P=0.04) | SBP (OR 1.02; 95% CI 1.00-1.03; P=0.02) |  |  |  |  |  |  |  |  |
| Nawar, 2019 [S24] | Brain infarction volume (OR 2.05; 95% CI 1.46-2.88; P<0.001) | NIHSS (OR 1.47; 95% CI 1.16-1.86; P<0.001) |  |  |  |  |  |  |  |  |  |  |  |
| Kufner, 2012 [S25] | FLAIR hyperintensity (OR 17.8; 95% CI 1.8-175.2; P=0.013) |  |  |  |  |  |  |  |  |  |  |  |  |
| Qiu, 2017 [S26] | Low fT3 (OR 0.204; 95% CI 0.065-0.642; P=0.007) |  |  |  |  |  |  |  |  |  |  |  |  |
| Rocco, 2013 [S27] | HbA1c (OR 9.07; 95% CI 5.01-16.42; P<0.001) | NIHSS on admission (OR 3.11; 95% CI 1.37-7.48; P=0.01) |  |  |  |  |  |  |  |  |  |  |  |
| Lee, 2019 [S28] | SBP≥170 mmHg (OR 3.247; 95% CI 0.998-10.571; P=0.050) | NIHSS≥15 (OR 7.020; 95% CI 1.816-27.134; P=0.005) | Platelet count <195000/mm^3^ (OR 5.389; 95% CI 1.491-19.481; P=0.010) |  |  |  |  |  |  |  |  |  |  |
| Puig, 2017 [S29] | HP_rs_-PCT 7ml/100g/min (OR 1.00; 95% CI 1.00-1.00; P=0.004) | NIHSS (OR 1.11; 95% CI 1.04-1.19; P=0.003) | Platelet count (OR 1.00; 95% CI 1.00-1.00; P=0.018) |  |  |  |  |  |  |  |  |  |  |
| Dharmasaroja, 2012 [S30] | INR≥1.0 (OR 4.89; 95% CI 1.11-21.49; P=0.036) | AF (OR 7.21; 95% CI 1.63-31.99; P=0.009) | Blood sugar > 8.325 mmol/L (OR 9.00; 95% CI 2.03-39.94; P=0.004) | NIHSS≥15 (OR 8.94; 95% CI 2.84-28.13; P<0.001) |  |  |  |  |  |  |  |  |  |
| Lin, 2018 (J Atheroscl-er Thromb) [S31] | TC (SICH by ECASS II) (RR 0.908; 95% CI 0.830-0.992; P= 0.0331) | LDL-C (SICH by NINDS) (RR 0.912; 95% CI 0.848-0.982; P=0.0145); LDL-C (SICH by ECASS II) (RR 0.823; 95% CI 0.742-0.913; P=0.0002); LDL-C (SICH by SITS-MOST) (RR 0.846; 95% CI 0.724-0.989; P=0.0358) | HDL-C (SICH by ECASS II) (RR 1.085; 95% CI 1.005-1.171; P=0.0379) | TG (SICH by SITS-MOST) (RR 0.837; 95% CI 0.722-0.970; P=0.0182) |  |  |  |  |  |  |  |  |  |
| Nisar, 2019 [S32] | MAP (NINDS) (95% CI 1.01-1.18; P=0.025) | Blood sugar ≥185 mg/dL (ECASS-II) (95% CI 0.12-0.45; P=0.001) | Presence of early infarct signs (ECASS-II) (95% CI 0.06-0.25; P=0.002) | Verify Aspirin less than 500 (NINDS) (95% CI 0.01-0.80; P=0.032) |  |  |  |  |  |  |  |  |  |
| Asuzu, 2015 (Neurocrit Care 2) [S33] | HDMCA sign (Log OR 1.26; SE 0.73) | early CT  hypodense-ty (Log OR 0.48; SE 0.65) | Visual field deficits (Log OR 0.07; SE 0.66) | Decreased LOC (Log OR 0.69; SE 0.65) |  |  |  |  |  |  |  |  |  |
| Khatri, 2010 [S34] | Microcat-heter injection (OR 3.60; 95% CI 1.12-11.49; P=0.031) | Glucose≥125 mg/dl (OR 3.78; 95% CI 1.34-10.65; P=0.012) | TICI (Thrombolysis in Cerebral Infarction scale) score (3, 2, 1, 0) Each one decrease (OR 1.82; 95% CI 1.04-3.20; P=0.038) |  |  |  |  |  |  |  |  |  |  |
| Lansberg, 2007 [S35] | Increase in DWI lesion volume (OR 1.42; 95% CI 1.13-1.78) |  |  |  |  |  |  |  |  |  |  |  |  |
| Lokeskr-awee, 2017 (CNR) [S36] | NIHSS > 20 (OR 3.51; 95% CI 2.18-5.65; P<0.001); NIHSS > 10 (OR 2.02; 95% CI 1.42-2.87; P<0.001) | Use of nicardipi-ne during rt-PA (OR 1.61; 95% CI 1.09-2.40; P=0.018) | Systolic blood pressure ≥140 mmHg (OR 1.47; 95% CI 1.06-2.04; p=0.021) | Platelet  count < 250000 cell/mm^3^ (OR 1.45; 95% CI 1.04-2.01; P=0.029) |  |  |  |  |  |  |  |  |  |
| Tomáš, 2018 [S37] | AF (OR 6.650, 95% CI 1.83-24.22; P=0.004) | NIHSS (OR 1.09, 95% CI 1.02-1.16; P=0.016) | Decreased level of conscious-sness (OR 2.12, 95% CI 1.28-3.51; P=0.003) | Occlusion of basilar artery (OR 6.60; 95% CI 1.67-26.15; P=0.007) | Occlusion of PCA (OR 11.06; 95% CI 2.75-44.49; P=0.001) | Additional endovasc-ular therapy (OR 8.711; 95% CI 2.539-29.88; P=0.001) | Supratent-orial territory PCA (OR 4.311, 95% CI 1.2-15.52; P=0.025) |  |  |  |  |  |  |
| Nagaraja, 2018 [S38] | NIHSS (OR 1.10; 95% CI 1.06-1.14; P<0.0001) | Nonlacun-ar infarct (OR 6.0; 95% CI 1.75-20.66; P=0.001) | Cerebral microble-eds (OR 2.72; 95% CI 1.45-5.10; P=0.003) | Race-Nonwhite (OR 2.5; 95% CI 1.1-5.4; P=0.017) |  |  |  |  |  |  |  |  |  |
| Ribo, 2004 [S39] | TAFI＞180% (OR 12.9; 95% CI 1.41-118.8; P=0.02) | PAI-1＜21.4 ng/mL (OR 12.75; 95% CI 1.17-139.2; P=0.04) |  |  |  |  |  |  |  |  |  |  |  |
| Selim, 2002 [S40] | Volume of ischemic tissue on DWI, with ADC≤ 550ⅹ10^-6^ mm^2^/s (OR 1.176; P=0.042) |  |  |  |  |  |  |  |  |  |  |  |  |
| Pjevic, 2021 [S41] | TT genotype of the MMP-9-1562C/T polymorph-hism (OR 13.08; 95% CI 1.04-165.09; P=0.047) |  |  |  |  |  |  |  |  |  |  |  |  |
| Shinoda, 2017 [S42] | Relative ADC ratio <0.650 (OR 7.79; 95% CI 2.22-27.3; P=0.001) | Use of tPA (OR 13.8; 95% CI 1.35-125.5; P=0.010) |  |  |  |  |  |  |  |  |  |  |  |
| Kimura, 2008 [S43] | The presence of recanalize-tion between 1 and 24 h after the end of t-PA infusion (OR 20.2; 95% CI 1.0~340.9; P=0.037) |  |  |  |  |  |  |  |  |  |  |  |  |
| Cho, 2013 [S44] | The presence of  micro- and macroalb-uminuria (OR 2.542; 95% CI 1.106-5.841; P=0.028) |  |  |  |  |  |  |  |  |  |  |  |  |
| Chen, 2018 [S45] | WMH (OR 1.562/10ml; 95% CI 1.215-2.009; P=0.001) |  |  |  |  |  |  |  |  |  |  |  |  |
| Brekenfe-ld, 2007 [S46] | Poor collaterals (OR 11.38; 95% CI 2.16-60.12; P=0.004) | Urokinase dose (OR 1.06; 95% CI 1.01-1.12; P=0.021) | Early signs on computed tomograp-hy (OR 5.99, 95% CI 1.24-28.82 P=0.026) |  |  |  |  |  |  |  |  |  |  |
| Das, 2020 [S47] | NIHSS (AOR 16.5; 95% CI 1.9-170.84; P=0.02) | Total anterior circulation stroke (AOR 6.1; 95% CI 1.3-32.45; P=0.04) | Fibrinogen (AOR 12.66; 95% CI 2.1-114.79; P=0.025) | CHADS2 score >2 (AOR 14.0; 95% CI 1.59-120.27; P=0.004) | Low ejection fraction (AOR 16.22; 95% CI 2.89-90.87; P=0.0056) | Higher mean arterial blood pressure (AOR 10.8; 95% CI 3.14-110.55; P=0.04) | Blood glucose level (AOR 11.45; 95% CI 2.19-96.45; P=0.03) | AF (AOR 10.2; 95% CI 1.82-60.46; P=0.028) | Anticoag-ulant therapy (AOR 6.19; 95% CI 1.84-30.8; P=0.029) | Antiplate-let  therapy (AOR 11.07; 95% CI 3.02-66.6; P=0.009) | SEDAN (AOR 9.25; 95% CI 2.37-5 1.87; P=0.03) | Low ASPECT (AOR 0.05; 95% CI 0.006-0.40; P=0.01) |  |
| Zou, 2013 [S48] | Hyperden-se middle cerebral artery sign (AOR 2.691; 95% CI 1.231-5.882; P=0.013) |  |  |  |  |  |  |  |  |  |  |  |  |
| Gensicke, 2016 [S49] | Proteinur-ia (AOR 1.54; 95% CI 1.09-2.17; P = 0.016) |  |  |  |  |  |  |  |  |  |  |  |  |
| Yang, 2020 [S50] | LDL-C (OR 0.638; 95% CI 0.412-0.989; P=0.045) | NIHSS (OR 1.101; 95% CI 1.034-1.172; P=0.003) | Lower albumin (OR 0.929; 95% CI 0.858-1.005; P=0.067) | MPV (OR 1.019; 95% CI 1.003-1.034; P=0.018) | UA (OR 0.997; 95% CI 0.994-1.000; P=0.087) |  |  |  |  |  |  |  |  |
| Luo, 2020 [S51] | TC/HDL-C<4.05 (AOR 1.727; 95% CI 1.008-2.960; P=0.047) | TG/HDL-C<0.82 (AOR 2.064; 95% CI 1.241-3.432; P=0.005) | LDL-C/HDL-C) <2.67 (AOR 1.935; 95% CI 1.070-3.501; P=0.029) |  |  |  |  |  |  |  |  |  |  |
| Castellanos, 2018 [S52] | Caveolin-1 levels ≤0.17 ng/Ml (OR 11.6; 95% CI 11.3-102.8; P=0.027) |  |  |  |  |  |  |  |  |  |  |  |  |
| Liu, 2016 [S53] | Low fT3 (OR 0.27; 95% CI 0.10-0.77; P=0.01) | Poor functional outcomes at discharge (OR 2.58; 95% CI 1.05-6.35; P=0.04) |  |  |  |  |  |  |  |  |  |  |  |
| Acampa, 2017 [S54] | ASI (OR 1.9; 95% CI 1.09-3.02) |  |  |  |  |  |  |  |  |  |  |  |  |
| Fàbregas, 2006 [S55] | Early signs of ischemia (OR 8.5; 95% CI 1.6-45.4; P=0.01) | Deviation from the protocol (OR 11.1; 95% CI 2.4-50; P=0.002) |  |  |  |  |  |  |  |  |  |  |  |
| Huynh, 2004 [S56] | Age 60-75 years (OR 3.4; 95% CI 1.6-7.2; P=0.001);  Age＞75 years (OR 6.9; 95% CI 3.2-14.7; P＜0.001) | Female sex (OR 1.6; 95% CI 1.0-2.6; P=0.043) | History of cerebrova-scular event (OR 2.4; 95% CI 1.3-4.7; P=0.007) | SBP＞160 mmHg (OR 2.2; 95% CI 1.3-3.6; P=0.003) |  |  |  |  |  |  |  |  |  |
| Lin, 2018 (Journal of the Neurological Sciences) [S57] | Blood sugar (ARR 1.891; 95% CI 0.977-3.657; P=0.0585 with the SITS-MOST criteria) and (ARR 1.884; 95% CI 1.138-3.121; P=0.0139 with the NINDS criteria) |  |  |  |  |  |  |  |  |  |  |  |  |
| Mundiyan-apurath, 2018 [S58] | Hypertens-ion (OR 1.39; 95% CI 1.08-1.80; P=0.012) | Minor stroke (OR 0.51; 95% CI 0.33-0.78; P=0.002) |  |  |  |  |  |  |  |  |  |  |  |
| Mendioroz, 2009 [S59] | APC (OR 25.19; 95% CI 4.76-133.19; P=0.0001) |  |  |  |  |  |  |  |  |  |  |  |  |
| Cho, 2008 [S60] | Focal fluid-attenuated inversion recovery  hyperintensity (SICH-1) (OR 13.64; 95% CI 1.51-123.28); (SICH-2) (OR 10.44; 95% CI 1.11-98.35) |  |  |  |  |  |  |  |  |  |  |  |  |
| Lin, 2020 [S61] | Stroke (OR 5.752; 95% CI 1.487-22.248; P=0.011) | AF (OR 5.428; 95% CI 1.427-20.640; P=0.013) |  |  |  |  |  |  |  |  |  |  |  |
| Kamal, 2021 [S62] | Lower levels of  albumin (OR 0.31; 95% CI 0.19-0.52; P<0.001) | HbA1c (OR 1.3; 95% CI 1.0-1.6; P=0.017) |  |  |  |  |  |  |  |  |  |  |  |
| Piccardi, 2015 [S63] | MMP9/TIMP1 (OR 1.74; 95% CI 1.17-2.57; P=0.006) | MMP9/TIMP2 (OR 1.71; 95% CI 1.16-2.52; P=0.007) |  |  |  |  |  |  |  |  |  |  |  |
| Cucchiara, 2009 [S64] | Baseline antiplatel-et use (Single antiplatel-et: OR 2.04; 95% CI 1.07-3.87; P=0.03; Double antiplatel-et: OR 9.29; 95% CI 3.28-26.32; P＜0.001) | NIHSS (OR 1.09; 95% CI 1.03-1.15; P=0.002) | CT changes defined by ASPECTS (ASPECTS 8 to 9: OR 2.26; 95% CI 0.63-8.10; P=0.21); (ASPECTS≤7: OR 5.63; 95% CI 1.66-19.10; P=0.006) |  |  |  |  |  |  |  |  |  |  |
| Dzwigal, 2013 [S65] | Older age (OR 0.968; 95% CI 0.932-1.007 | Female sex (OR 0.815; 95% CI 0.370-1.796) | Blood glucose level (OR 0.909; 95% CI 0.778-1.062) | high NIHSS score (>14) (OR 1.101; 95% CI 1.020-1.187) |  |  |  |  |  |  |  |  |  |
| Yu, 2020 [S66] | The calcificat-ion volume on the lesion side (OR 1.504; 95% CI 1.140-1.985; P=0.004) |  |  |  |  |  |  |  |  |  |  |  |  |
| Selker, 1994 [S67] | Pulse pressure (OR 1.76; 95% CI 1.25-2.46; P=0.001) | Age (OR 1.84; 95% CI 1.05-3.22; P=0.03) |  |  |  |  |  |  |  |  |  |  |  |
| Curtze, 2015 [S68] | High load of Cerebral white matter lesions (WMLs) (OR 4.11; 95% CI 2.38-7.10) |  |  |  |  |  |  |  |  |  |  |  |  |
| Qiu, 2021 [S69] | The length of periventri-cular  transit time to the peak (OR 4.740; 95% CI 1.624-13.837; P=0.004) | NIHSS (OR 1.175; 95% CI 1.076-1.282; P<0.001) | The infarct core volume≥5.5mL (OR 3.286; 95% CI 1.223-8.829; P=0.018) |  |  |  |  |  |  |  |  |  |  |
| Haefelin, 2006 [S70] | Leukoara-iosis (OR 2.9; 95% CI 1.29-6.59; P=0.015) |  |  |  |  |  |  |  |  |  |  |  |  |
| Willer, 2015 [S71] | Leukoara-iosis (OR 2.4; 95% CI 1.40-5.78; P=0.04) |  |  |  |  |  |  |  |  |  |  |  |  |
| Tsetsou, 2016 [S72] | The lowest cerebral blood volume (CBV) in the core (OR 0.01; P=0.009) | Lower body weight (OR 0.96 per kg; P=0.024) |  |  |  |  |  |  |  |  |  |  |  |
| Xu, 2017 [S73] | Age≥80 years (OR 7.875; 95% CI 1.199-51.749; P=0.032) | The level of mean systolic  pressure (MSP)≥140 mmHg (OR 9.417; 95% CI 1.277-69.449; P=0.028) | NIHSS (OR 1.161; 95% CI 1.035-1.302; P=0.011) | Fibrinogen  concentration (OR 3.633; 95% CI 1.278-10.328; P=0.016) |  |  |  |  |  |  |  |  |  |
| Tong, 2014 [S74] | Age 65-74 years (AOR 2.30; 95% CI 1.29-4.08;  P=0.005); 75-79 years (AOR 3.01; 95% CI 1.62-5.60;  P=0.001); 80+ years (AOR 3.09; 95% CI 1.73-5.51;  P＜0.001) | NIHSS (AOR 1.06; 95% CI 1.05-1.08; P<0.0001) | History of dyslipid-emia (AOR 1.34; 95% CI 1.05-1.70; P=0.02) |  |  |  |  |  |  |  |  |  |  |
| Brass, 2000 [S75] | Age≥75 years (OR 1.57; 95% CI 1.30-1.90; P=0.0001) | Females sex (OR 1.39; 95% CI 1.15-1.67; P=0.0007) | Black  Race (OR 1.63; 95% CI 1.13-2.37; P=0.0096) | Prior stroke (OR 1.48; 95% CI 1.10-2.00; P=0.0099) | Blood pressure ≥160 mm Hg (OR 1.82; 95% CI 1.51-2.21; P=0.0001) | Tissue plasmino-gen activator (versus other thrombol-ytic agent) (OR 1.57; 95% CI 1.23-2.01; P=0.0003) | Excessive anticoagu-lation (internati-onal normalize-ed ratio ≥4 or prothrom-bin time ≥24) (OR 2.15; 95% CI 1.10-4.22; P=0.0257) | Below median weight (≤65  kg for women; ≤80 kg for men) (OR 1.47; 95% CI 1.21-1.77; P=0.0001) |  |  |  |  |  |
| Derex, 2005 [S76] | Mean 24 hour  systolic blood pressure (OR 1.078; 95% CI 1.027-1.131; P=0.003) | A hyperden-se artery sign on pretreatm-ent CT (OR 6.630; 95% CI 1.323-33.235; P=0.02) |  |  |  |  |  |  |  |  |  |  |  |
| Sun, 2020 [S77] | Age≥68years (OR 1.835; 95% CI 1.361-2.826; P=0.024) | Smoke (OR 2.352; 95% CI 2.174-3.580; P=0.016) | AF (OR 2.140; 95% CI 1.492-2.453; P=0.028) | NIHSS≥17 (OR 1.783; 95% CI 1.156-3.247; P=0.008) | SBP≥149 (OR 4.251; 95% CI 2.663-5.710; P=0.004) | Caveolin-1 (ng/mL) ≤0.12 (OR 1.972; 95% CI 1.291-3.186; P=0.031) | Caveolin-2 (ng/mL) ≤0.43 (OR 2.759; 95% CI 1.685-4.462; P=0.005) |  |  |  |  |  |  |
| Xing, 2014 [S78] | Higher globulin level (OR 1.185; 95% CI 1.090-1.288; P<0.001) | PTA (OR 1.016; 95% CI 1.003-1.029; P=0.018) | White blood cell count (OR 1.097; 95% CI 1.012-1.190; P=0.025) | NIHSS score (OR 1.097; 95% CI 1.031-1.166; P = 0.003) |  |  |  |  |  |  |  |  |  |
| JAEGERE, 1992 [S79] | Patients taking an oral anticoagulant before admission (OR 5.7; 95% CI 1.1-29.4) | Body weight<70 kg (OR 3.7; 95% CI 1.4-9.8) | Age>65 years (OR 3.3; 95% CI 1.2-10.0) |  |  |  |  |  |  |  |  |  |  |
| Tütüncü, 2013 [S80] | RI (GFR<30mL/min) (OR 3.80; 95% CI 1.45-9.96; P＜0.01) | csGFR (OR 1.61; 95% CI 1.16-2.23; P＜0.01) |  |  |  |  |  |  |  |  |  |  |  |
| Jucevičiūtė, 2019 [S81] | AEC≥0.11×10^9^/L (AOR 0.223; 95% CI 0.069-0.723; P=0.012) | Baseline NIHSS (OR 1.116; 95% CI 1.005-1.239; P=0.041) |  |  |  |  |  |  |  |  |  |  |  |
| Cougo-Pinto, 2012 [S82] | Current statin treatment (OR 3.8; 95% CI 2.5-8.2; P＜0.001) |  |  |  |  |  |  |  |  |  |  |  |  |
| Muengta-weepongsa, 2017 [S83] | Glucose >12 mmol/l (OR 1.276; 95% CI 1.087-4.135; P=0.031) | Early infarction (OR 2.501; 95% CI 1.684-8.135; P<0.001) | Hyperden-se cerebral  artery (OR 1.093; 95% CI 0.923-3.765; P=0.048) | Age>75 years (OR 1.535; 95% CI 1.157-4.998; P=0.024) | NIHSS ≥10 (OR 1.253; 95% CI 0.954-4.653; P=0.029) | AF (OR 2.492; 95% CI 1.378-7.802; P=0.023) |  |  |  |  |  |  |  |
| Shin, 2017 [S84] | Overused alteplase (OR 7.26; 95% CI 1.24-42.45; P=0.028); Use alteplase overdose (OR 1.67; 95% CI 1.05-2.66; P=0.027) | Baseline glucose (>144mg/Dl; OR 5.03; 95% CI 1.00-25.26; P=0.050) | NIHSS (OR 1.13; 95% CI 1.00-1.27; P=0.047) |  |  |  |  |  |  |  |  |  |  |
| Kandzari, 2004 [S85] | Recent facial or head trauma (OR 13.0; 95% CI 3.4-85.5) | Weight＜70kg (OR 2.2; 95% CI 1.6-3.1) | Diastolic blood pressure  ≥95 mm Hg (OR 1.9; 95% CI 1.3-2.9) |  |  |  |  |  |  |  |  |  |  |
| Wang, 2019 [S86] | Smoke (OR 0.07; 95% CI 0.010-0.532; P=0.01) | APTT＞30.3s (OR 2.13; 95% CI 1.020-4.435; P=0.04) | FIB≥1.50 (OR 0.08; 95% CI 0.010-0.654; P=0.02) | PLT≥185 (OR 0.47; 95% CI 0.239-0.911; P=0.03) |  |  |  |  |  |  |  |  |  |
| Szegedi, 2019 [S87] | PAI-1 5G/5G genotype (OR 4.75; 95% CI 1.18-19.06; P=0.028) | Hyperlipidemia (OR 0.21; 95% CI 0.05-0.88; P=0.033) |  |  |  |  |  |  |  |  |  |  |  |
| Sun, 2015 [S88] | Log FDP (OR 7.50; 95% CI 1.26-44.61; P=0.03) | Log fibrinogen (OR 19.32; 95% CI 1.81-205.98; P=0.01) |  |  |  |  |  |  |  |  |  |  |  |
| Vandelli, 2015 [S89] | Fibrinogen＜2.0g/L and (or) decreased＞25% (OR 7.47; 95% CI 2.26-24.74; P＜0.001) | Baseline NIHSS (OR 1.15; 95% CI 1.06-1.25; P＜0.001) |  |  |  |  |  |  |  |  |  |  |  |
| Christoforidis, 2009 [S90] | Pial collateral formation (OR 3.03; 95% CI 1.09-8.71; P=0.0342) | Diabetes (OR 4.82; 95% CI 1.49-16.9; P=0.0100) | Platelets＜200000/uL (OR 2.95; 95% CI 1.06-8.57; P=0.0403) | Time to treat ＞180 min (OR 12.0; 95% CI 1.77-253; P=0.0333) |  |  |  |  |  |  |  |  |  |
| Liu, 2017 [S91] | Age≧70 years-old (NINDS: AOR 1.73; 95% CI 1.02-2.95; P=0.04) | Diabetes (mSITS-SICH: AOR 3.5; 95% CI 1.34-9.16; P=0.01) | Serum glucose on admission >9.0 mmol/L (ECASS II: AOR 2.84; 95% CI 1.48-5.46; P=0.002) | NIHSS＞20 (mSITS-SICH: AOR 5.06; 95% CI 1.68-15.20; P=0.004 or NINDS: AOR 2.81; 95% CI 1.42-5.57; P=0.003) | Cardioe-mbolism (ECASSII: AOR 4.99; 95% CI 2.53-9.84; P<0.001) |  |  |  |  |  |  |  |  |
| Foerch, 2007 [S92] | S100B (OR 2.80; 95% CI 1.4-5.62; P=0.004) |  |  |  |  |  |  |  |  |  |  |  |  |
| Marsh, 2013 [S93] | Serum creatinine＞1.0 mg/dL (OR 5.5; 95% CI 1.08-28.39) |  |  |  |  |  |  |  |  |  |  |  |  |
| Castellanos, 2004 [S94] | c-Fn plasma levels (OR 2.1; 95% CI 1.3-3.4; P=0.002) |  |  |  |  |  |  |  |  |  |  |  |  |
| Che, 2017 [S95] | Low serum albumin level (OR 4.369; 95% CI 1.626-11.742; P=0.003) |  |  |  |  |  |  |  |  |  |  |  |  |
| Yao, 2016 [S96] | Homocys-teine level>23.86 mol/L (OR 13.65; 95% CI 3.58-51.97; P<0.001) | NIHSS on admission (OR 1.61; 95% CI 1.38-1.86; P<0.001) |  |  |  |  |  |  |  |  |  |  |  |
| Gnofam, 2013 [S97] | Baseline serum glucose concentra-tion (OR 1.176; 95% CI 1.020-1.357; P=0.025) | Baseline NIHSS  score  (OR 1.108; 95% CI 1.044-1.176; P=0.001) | Baseline SBP  (OR 1.021; 95% CI 1.003-1.039; P=0.020) |  |  |  |  |  |  |  |  |  |  |
| González, 2012 [S98] | Platelet derived growth factor-CC (PDGF-CC) levels on admission (OR 1.02; 95% CI 1.00-1.04; P=0.032) | Serum levels of PDGF-CC at 24 h (OR 1.05; 95% CI 1.02-1.08; P=0.004) |  |  |  |  |  |  |  |  |  |  |  |
| Tsivgoulis, 2009 [S99] | Blood pressure protocol violations (SBP  ＞185 or DBP＞110 mm Hg) (OR 2.59; 95% CI 1.07-6.25; P=0.034) | Higher  baseline stroke severity (OR per 1-point increase in National  Institutes of Health Stroke Scale 1.11; 95% CI 1.05-1.18; P=0.001) | Lipid-lowering medicate-on use before stroke  onset (OR 2.41; 95% CI 1.09-5.33; P=0.030) | SBP (OR 1.22; 95% CI 1.06-1.41; P=0.008) |  |  |  |  |  |  |  |  |  |
| Palumbo, 2007 [S100] | Severe leukoarai-osis (RR 2.75; 95% CI 1.15-6.53; P=0.03) | Multiple  lacunes (RR 3.4; 95% CI 1.5-7.68; P=0.008) |  |  |  |  |  |  |  |  |  |  |  |
| James, 2018 [S101] | Change in level of conscious-sness (OR 6.62; 95% CI 1.64-26.70; P=0.008) |  |  |  |  |  |  |  |  |  |  |  |  |
| Li, 2017 [S102] | High K^trans^ value (OR 5.04; 95% CI 2.01-12.65; P＜0.01) |  |  |  |  |  |  |  |  |  |  |  |  |
| Zhu, 2021 [S103] | Apelin (OR 0.241; 95% CI 0.086-0.672; P≤0.01) | Vaspin (OR 0.259; 95% CI 0.115-0.586; P≤0.01) | IL-1β (OR 1.058; 95% CI 1.030-1.088; P≤0.01) | IL-6 (OR 1.534; 95% CI 1.116-2.107; P≤0.01) | Malondia-ldehyde (MDA) (OR 2.494; 95% CI 1.324-4.698; P≤0.01) | Superoxi-de (SOD) dismutase level (OR 0.197; 95% CI 0.052-0.735; P≤0.01) |  |  |  |  |  |  |  |
| Guo, 2016 [S104] | The neutrophi-l to lymphocy-te ratio (NLR)≥10.59 (for PH, OR 8.50; 95% CI 2.69-26.89; P < 0.001) and (for SICH, OR 7.93; 95% CI 2.25-27.99; P = 0.001) | NLR after treated 12-18h (for PH, AOR 1.14; 95% CI 1.05-1.23) and (for SICH, AOR 1.14; 95% CI 1.06-1.23) |  |  |  |  |  |  |  |  |  |  |  |
| Lakhter, 2020 [S105] | Stroke (OR 19.4; 95% CI 8.8-42.8; P<0.01) | CKD (OR 2.2; 95% CI 1.1-4.7; P=0.03) | Age >74 years old (OR 2.2; 95% CI 1.2-4.3; P=0.02) | Males (OR 1.8; 95% CI 1.01-3.3; P=0.048) |  |  |  |  |  |  |  |  |  |

**Table 2: Risk of bias assessments using PROBAST for risk assessment model studies**

| **Author** | **Year** | **Participants** | **Predictors** | **Outcome** | **Analysis** | **Overall** |
| --- | --- | --- | --- | --- | --- | --- |
| **Barber** | 2000 | + | + | + | - | - |
| **Kawano** | 2012 | + | + | + | - | - |
| **Singer** | 2009 | + | + | + | - | - |
| **Espı´nola** | 2012 | + | + | + | - | - |
| **Menon** | 2012 | + | + | - | + | - |
| **Tanne** | 2002 | + | + | - | - | - |
| **Chatterjee** | 2017 | + | + | - | - | - |
| **Lokeskrawee** | 2017 | + | + | + | - | - |
| **Mazya** | 2012 | + | + | - | - | - |
| **Wahlgren** | 2008 | + | + | - | - | - |
| **Saposnik** | 2013 | + | + | + | - | - |
| **Cappellari** | 2018 | + | + | + | - | - |
| **Flint** | 2013 | + | + | + | - | - |
| **Flint** | 2015 | + | + | - | - | - |
| **Asuzu** | 2015 | + | + | - | - | - |
| **Zhou** | 2020 | + | + | - | + | - |
| **Erdur** | 2018 | + | + | + | - | - |
| **Kidwell** | 2002 | + | + | + | - | - |
| **Soni** | 2021 | + | + | + | - | - |
| **Wu** | 2020 | + | + | + | - | - |
| **Simoons** | 1993 | + | + | - | - | - |
| **Guo** | 2021 | + | + | + | - | - |

**Table 3: Risk of bias assessments using QUIPS for prognostic factor studies**

| **Author** | **Year** | **Study**  **partici-pation** | **Study**  **attrition** | **Prognostic**  **factor**  **measurem-**  **ent** | **Outcome**  **measure-ment** | **Study**  **confound-ing** | **Statistical**  **analysis and**  **reporting** |
| --- | --- | --- | --- | --- | --- | --- | --- |
| **Larrue** | 2001 | Yes | Not  reported | Yes | Yes | No | No |
| **Nawar** | 2019 | Yes | 0.63 | Yes | Yes | No | No |
| **Kufner** | 2012 | Yes | Not  reported | Yes | Yes | No | No |
| **Qiu** | 2017 | Yes | 4.22 | Yes | Yes | Yes | No |
| **Rocco** | 2013 | Yes | 0.18 | Yes | Yes | No | No |
| **Lee** | 2019 | Yes | 2.13 | Yes | Yes | No | Yes |
| **Puig** | 2017 | Yes | 6.44 | Yes | Yes | Yes | Yes |
| **Dharmas-aroja** | 2012 | Yes | 1.03 | Yes | Yes | Yes | Yes |
| **Lin** | 2018 | Yes | 28.62 | Yes | Yes | Yes | No |
| **Nisar** | 2019 | Yes | 16.85 | Yes | Yes | No | No |
| **Asuzu** | 2015 | Yes | 0.47 | Yes | Yes | No | Yes |
| **Khatri** | 2010 | Yes | Not  reported | Yes | No | No | No |
| **Lansberg** | 2007 | No | Not  reported | Yes | Yes | No | Yes |
| **Lokeskra-wee** | 2017 | Yes | 1.18 | Yes | Yes | No | No |
| **Tomáš** | 2018 | Yes | 0 | Yes | Yes | No | No |
| **Nagaraja** | 2018 | Yes | 17.45 | Yes | Yes | Yes | No |
| **Ribo** | 2004 | Yes | 0 | Yes | Yes | Yes | Yes |
| **Selim** | 2002 | Yes | Not  reported | Yes | Yes | Yes | No |
| **Pjevic** | 2021 | Yes | 2.41 | Yes | No | No | No |
| **Shinoda** | 2017 | Yes | 23.81 | Yes | Yes | No | Yes |
| **Kimura** | 2008 | Yes | 0 | Yes | Yes | No | No |
| **Cho** | 2013 | Yes | 1.23 | Yes | Yes | No | No |
| **Chen** | 2018 | Yes | 2.41 | Yes | Yes | Yes | No |
| **Brekenfe-ld** | 2007 | Yes | Not  reported | Yes | Yes | No | No |
| **Das** | 2020 | Yes | Not  reported | Yes | Yes | No | No |
| **Zou** | 2013 | Yes | 0 | Yes | Yes | No | Yes |
| **Gensicke** | 2016 | Yes | 2.24 | Yes | Yes | Yes | Yes |
| **Yang** | 2020 | Yes | Not  reported | Yes | Yes | No | No |
| **Luo** | 2020 | Yes | 5.22 | Yes | Yes | No | No |
| **Castellan-os** | 2018 | Yes | 0 | Yes | Yes | Yes | Yes |
| **Liu** | 2016 | Yes | 0 | Yes | Yes | No | No |
| **Acampa** | 2017 | Yes | 0 | Yes | Yes | No | No |
| **Fàbregas** | 2006 | Yes | Not  reported | Yes | Yes | No | No |
| **Huynh** | 2004 | Yes | 0 | Yes | Yes | No | No |
| **Lin** | 2018 | Yes | 11.01 | Yes | Yes | No | No |
| **Mundiya-napurath** | 2018 | Yes | 3.46 | Yes | Yes | No | Yes |
| **Mendior-oz** | 2009 | Yes | 1.71 | Yes | Yes | Yes | Yes |
| **Cho** | 2008 | Yes | Not  reported | Yes | Yes | Yes | No |
| **Lin** | 2020 | Yes | Not  reported | Yes | Yes | Yes | No |
| **Kamal** | 2021 | Yes | Not  reported | Yes | Yes | Yes | No |
| **Piccardi** | 2015 | Yes | 0 | Yes | Yes | Yes | No |
| **Cucchiara** | 2009 | Yes | 0 | Yes | Yes | No | No |
| **Dzwigal** | 2013 | Yes | 0 | Yes | Yes | No | Yes |
| **Yu** | 2020 | No | Not  reported | Yes | Yes | No | No |
| **Selker** | 1994 | Yes | 1.69 | Yes | Yes | No | No |
| **Curtze** | 2015 | Yes | 0.81 | Yes | Yes | Yes | No |
| **Qiu** | 2021 | Yes | 5.12 | Yes | Yes | Yes | Yes |
| **Haefelin** | 2006 | Yes | Not  reported | Yes | Yes | No | No |
| **Willer** | 2015 | Yes | Not  reported | Yes | Yes | Yes | No |
| **Tsetsou** | 2016 | Yes | Not  reported | Yes | Yes | No | No |
| **Xu** | 2017 | Yes | 4.14 | Yes | Yes | No | No |
| **Tong** | 2014 | Yes | Not  reported | Yes | Yes | No | No |
| **Brass** | 2000 | Yes | Not  reported | Yes | No | No | No |
| **Derex** | 2005 | Yes | 4.26 | Yes | Yes | No | No |
| **Sun** | 2020 | Yes | 0 | Yes | Yes | Yes | Yes |
| **Xing** | 2014 | Yes | Not  reported | Yes | Yes | No | Yes |
| **JAEGERE** | 1992 | Yes | 0.12 | Yes | Yes | No | No |
| **Tütüncü** | 2013 | Yes | 17.00 | Yes | Yes | Yes | No |
| **Jucevičiū-tė** | 2019 | Yes | 0 | Yes | Yes | No | No |
| **Cougo-Pinto** | 2012 | Yes | 3.42 | Yes | Yes | No | No |
| **Muengta-weepongsa** | 2017 | Yes | Not  reported | Yes | Yes | No | No |
| **Shin** | 2017 | Yes | 0 | Yes | Yes | Yes | Yes |
| **Kandzari** | 2004 | Yes | Not  reported | Yes | Yes | No | No |
| **Wang** | 2019 | Yes | 0 | Yes | Yes | No | No |
| **Szegedi** | 2019 | Yes | 0 | Yes | Yes | No | No |
| **Sun** | 2015 | Yes | Not  reported | Yes | Yes | Yes | No |
| **Vandelli** | 2015 | Yes | 0 | Yes | Yes | No | No |
| **Christofo-ridis** | 2009 | Yes | 7.14 | Yes | Yes | No | No |
| **Liu** | 2017 | Yes | Not  reported | Yes | Yes | No | No |
| **Foerch** | 2007 | Yes | Not  reported | Yes | Yes | No | No |
| **Marsh** | 2013 | Yes | 1.32 | Yes | Yes | No | No |
| **Castellan-os** | 2004 | Yes | Not  reported | Yes | Yes | No | No |
| **Che** | 2017 | Yes | 0 | Yes | Yes | Yes | Yes |
| **Yao** | 2016 | Yes | Not  reported | Yes | Yes | No | No |
| **Gnofam** | 2013 | Yes | 0.20 | Yes | Yes | Yes | Yes |
| **González** | 2012 | Yes | 0 | Yes | Yes | Yes | Yes |
| **Tsivgoulis** | 2009 | Yes | 4.49 | Yes | Yes | Yes | No |
| **Palumbo** | 2007 | Yes | 10.22 | Yes | Yes | Yes | Yes |
| **James** | 2018 | Yes | Not  reported | Yes | Yes | No | Yes |
| **Li** | 2017 | Yes | 1.30 | Yes | Yes | No | No |
| **Zhu** | 2021 | Yes | Not  reported | Yes | Yes | No | No |
| **Guo** | 2016 | Yes | 0 | Yes | Yes | Yes | Yes |
| **Lakhter** | 2020 | Yes | Not  reported | Yes | No | No | No |

**Table 4. Evidence profile for bleeding-related prognostic factors**

| **No. of**  **studies** | **Certainty assessment domains** | | | | | | **Overall certainty in the evidence about this prognostic factor** | **Relative effect**  **(95% CI)** | **P values** |
| --- | --- | --- | --- | --- | --- | --- | --- | --- | --- |
|  | **Study design** | **Risk of bias** | **Indirect** | **Inconsistent** | **Imprecise** | **Publicati-on bias** |  |  |  |
| **TAFI [S39]** | | | | | | | | | |
| 1 | Observational | Not serious | Not serious | Not serious | Not serious | Undetected | ㊉㊉㊉㊉  HIGH | OR，12.90；  95%CI，1.41-118.01 | 0.02 |
| **PAI-1** **[S39]** | | | | | | | | | |
| 1 | Observational | Not serious | Not serious | Not serious | Not serious | Undetected | ㊉㊉㊉㊉  HIGH | OR，12.75；  95%CI，1.17-138.95 | 0.04 |
| **APC [S59]** | | | | | | | | | |
| 1 | Observational | Not serious | Not serious | Not serious | Not serious | Undetected | ㊉㊉㊉㊉  HIGH | OR，25.19；  95%CI，4.76-133.30 | ＜0.001 |
| **Age** **[S5, S7, S9-10, S12-14, S17, S19, S21, S23, S56, S65, S67, S73-75, S77, S79, S83, S91, S105]** | | | | | | | | | |
| 25 | Observational | Serious ^a^ | Not serious | Not serious | Not serious | Undetected | ㊉㊉㊉◯  MODERATE | OR，1.77；  95%CI，1.52-2.07 | ＜0.001 |
| **Race** **[S5, S38, S75]** | | | | | | | | | |
| 3 | Observational | Serious ^a^ | Not serious | Not serious | Not serious | Undetected | ㊉㊉㊉◯  MODERATE | OR，1.86；  95%CI，1.40-2.45 | ＜0.001 |
| **ASPECT** **[S1-3, S47, S64]** | | | | | | | | | |
| 6 | Observational | Serious ^a^ | Not serious | Not serious | Not serious | Undetected | ㊉㊉㊉◯  MODERATE | OR，1.97；  95%CI，1.25-3.12 | 0.004 |
| **NIHSS** **[S4-6, S8-10, S12-20, S22, S24, S27-30, S36-38, S47, S50, S64-65, S69, S73-74, S77-78, S81, S83-84, S89, S91, S96-97, S99]** | | | | | | | | | |
| 46 | Observational | Serious ^a^ | Not serious | Not serious | Not serious | Undetected | ㊉㊉㊉◯  MODERATE | OR，1.27；  95%CI，1.22-1.33 | ＜0.001 |
| **mRS** **[S15, S53]** | | | | | | | | | |
| 2 | Observational | Serious ^a^ | Not serious | Not serious | Not serious | Undetected | ㊉㊉㊉◯  MODERATE | OR，1.65；  95%CI，1.19-2.27 | 0.002 |
| **TICI** **[S34]** | | | | | | | | | |
| 1 | Observational | Serious ^a^ | Not serious | Not serious | Not serious | Undetected | ㊉㊉㊉◯  MODERATE | OR，1.82；  95%CI，1.04-3.18 | 0.04 |
| **CHADS2 score** **[S47]** | | | | | | | | | |
| 1 | Observational | Serious ^a^ | Not serious | Not serious | Not serious | Undetected | ㊉㊉㊉◯  MODERATE | OR，14.00；  95%CI，1.59-123.28 | 0.02 |
| **Low EF [S47]** | | | | | | | | | |
| 1 | Observational | Serious ^a^ | Not serious | Not serious | Not serious | Undetected | ㊉㊉㊉◯  MODERATE | OR，16.22；  95%CI，2.89-91.03 | 0.002 |
| **SEDAN [S47]** | | | | | | | | | |
| 1 | Observational | Serious ^a^ | Not serious | Not serious | Not serious | Undetected | ㊉㊉㊉◯  MODERATE | OR，9.25；  95%CI，2.37-36.10 | 0.001 |
| **Arterial stiffness index (ASI) [S54]** | | | | | | | | | |
| 1 | Observational | Serious ^a^ | Not serious | Not serious | Not serious | Undetected | ㊉㊉㊉◯  MODERATE | OR，1.90；  95%CI，1.09-3.31 | 0.02 |
| **K ^trans^ [S102]** | | | | | | | | | |
| 1 | Observational | Serious ^a^ | Not serious | Not serious | Not serious | Undetected | ㊉㊉㊉◯  MODERATE | OR，5.04；  95%CI，2.01-12.64 | ＜0.001 |
| **Peripheral vascular disease (PVD)** **[S7]** | | | | | | | | | |
| 1 | Observational | Serious ^a^ | Not serious | Not serious | Not serious | Undetected | ㊉㊉㊉◯  MODERATE | OR，1.59；  95%CI，1.12-2.26 | 0.01 |
| **Cerebral small vascular diseases (CSVD)** **[S20, S38, S70-71, S100]** | | | | | | | | | |
| 5 | Observational | Serious ^a^ | Not serious | Not serious | Not serious | Undetected | ㊉㊉㊉◯  MODERATE | OR，2.69；  95%CI，1.98-3.66 | ＜0.001 |
| **Cerebral microbleeds** **[S38]** | | | | | | | | | |
| 1 | Observational | Serious ^a^ | Not serious | Not serious | Not serious | Undetected | ㊉㊉㊉◯  MODERATE | OR，2.72；  95%CI，1.45-5.10 | 0.002 |
| **Leukoaraiosis** **[S70-71, S100]** | | | | | | | | | |
| 3 | Observational | Serious ^a^ | Not serious | Not serious | Not serious | Undetected | ㊉㊉㊉◯  MODERATE | OR，2.61；  95%CI，1.74-3.91 | ＜0.001 |
| **Poor collaterals** **[S46, S90]** | | | | | | | | | |
| 2 | Observational | Serious ^a^ | Not serious | Not serious | Not serious | Undetected | ㊉㊉㊉◯  MODERATE | OR，4.36；  95%CI，1.82-10.41 | ＜0.001 |
| **Recent facial or head trauma** **[S85]** | | | | | | | | | |
| 1 | Observational | Serious ^a^ | Not serious | Not serious | Not serious | Undetected | ㊉㊉㊉◯  MODERATE | OR，13.00；  95%CI，3.40-49.70 | ＜0.001 |
| **Cerebral artery occlusion** **[S37]** | | | | | | | | | |
| 2 | Observational | Serious ^a^ | Not serious | Not serious | Not serious | Undetected | ㊉㊉㊉◯  MODERATE | OR，8.52；  95%CI，3.20-22.64 | ＜0.001 |
| **Decreased levels of consciousness** **[S33, S37, S101]** | | | | | | | | | |
| 3 | Observational | Serious ^a^ | Not serious | Not serious | Not serious | Undetected | ㊉㊉㊉◯  MODERATE | OR，2.36；  95%CI，1.51-3.68 | ＜0.001 |
| **Cardiovascular disease** **[S4, S7-9, S13-14, S16, S19-23, S30, S37, S47, S58, S61, S77, S83, S91]** | | | | | | | | | |
| 22 | Observational | Serious ^a^ | Not serious | Not serious | Not serious | Undetected | ㊉㊉㊉◯  MODERATE | OR，2.09；  95%CI，1.75-2.49 | ＜0.001 |
| **Prior myocardial infarction** **[S7]** | | | | | | | | | |
| 1 | Observational | Serious ^a^ | Not serious | Not serious | Not serious | Undetected | ㊉㊉㊉◯  MODERATE | OR，1.80；  95%CI，1.33-2.44 | ＜0.001 |
| **Valvular heart diseases** **[S8]** | | | | | | | | | |
| 1 | Observational | Serious ^a^ | Not serious | Not serious | Not serious | Undetected | ㊉㊉㊉◯  MODERATE | OR，2.09；  95%CI，1.07-4.08 | 0.03 |
| **Hypertension** **[S9, S13-14, S19-21, S58]** | | | | | | | | | |
| 7 | Observational | Serious ^a^ | Not serious | Not serious | Not serious | Undetected | ㊉㊉㊉◯  MODERATE | OR，1.42；  95%CI，1.21-1.67 | ＜0.001 |
| **AF** **[S4, S13-14, S16, S19-20, S22, S30, S37, S47, S61, S77, S83]** | | | | | | | | | |
| 13 | Observational | Serious ^a^ | Not serious | Not serious | Not serious | Undetected | ㊉㊉㊉◯  MODERATE | OR，2.62；  95%CI，1.92-3.59 | ＜0.001 |
| **Congestive heart failure** **[S23]** | | | | | | | | | |
| 1 | Observational | Serious ^a^ | Not serious | Not serious | Not serious | Undetected | ㊉㊉㊉◯  MODERATE | OR，2.57；  95%CI，1.16-5.69 | 0.02 |
| **Diabetes** **[S13-14, S20, S90-91]** | | | | | | | | | |
| 5 | Observational | Serious ^a^ | Not serious | Not serious | Not serious | Undetected | ㊉㊉㊉◯  MODERATE | OR，1.84；  95%CI，1.34-2.51 | ＜0.001 |
| **Blood sugar** **[S1, S5-6, S9-10, S12, S16-19, S22, S30, S32, S34, S47, S57, S65, S83-84, S91, S97]** | | | | | | | | | |
| 22 | Observational | Serious ^a^ | Not serious | Not serious | Not serious | Undetected | ㊉㊉㊉◯  MODERATE | OR，1.14；  95%CI，1.10-1.20 | ＜0.001 |
| **PDGF-CC** **[S98]** | | | | | | | | | |
| 2 | Observational | Not serious | Not serious | Not serious | Serious ^c^ | Undetected | ㊉㊉㊉◯  MODERATE | OR，1.03；  95%CI，1.00-1.06 | 0.02 |
| **BP protocol violations** **[S99]** | | | | | | | | | |
| 1 | Observational | Serious ^a^ | Not serious | Not serious | Not serious | Undetected | ㊉㊉㊉◯  MODERATE | OR，2.59；  95%CI，1.07-6.27 | 0.03 |
| **SBP** **[S5, S8-10, S12, S17, S19, S23, S28, S36, S56, S75-77, S97, S99]** | | | | | | | | | |
| 17 | Observational | Serious ^a^ | Not serious | Not serious | Not serious | Undetected | ㊉㊉㊉◯  MODERATE | OR，1.15；  95%CI，1.10-1.20 | ＜0.001 |
| **Pulse pressure** **[S19, S67]** | | | | | | | | | |
| 2 | Observational | Serious ^a^ | Not serious | Not serious | Not serious | Undetected | ㊉㊉㊉◯  MODERATE | OR，2.37；  95%CI，1.01-5.57 | 0.05 |
| **INR** **[S30, S75]** | | | | | | | | | |
| 2 | Observational | Serious ^a^ | Not serious | Not serious | Not serious | Undetected | ㊉㊉㊉◯  MODERATE | OR，2.47；  95%CI，1.34-4.55 | 0.004 |
| **APTT [S86]** | | | | | | | | | |
| 1 | Observational | Serious ^a^ | Not serious | Not serious | Not serious | Undetected | ㊉㊉㊉◯  MODERATE | OR，2.13；  95%CI，1.02-4.45 | 0.04 |
| **TC [S31]** | | | | | | | | | |
| 1 | Observational | Serious ^a^ | Not serious | Not serious | Not serious | Undetected | ㊉㊉㊉◯  MODERATE | OR，0.91；  95%CI，0.83-0.99 | 0.04 |
| **LDL-C** **[S31, S50]** | | | | | | | | | |
| 4 | Observational | Serious ^a^ | Not serious | Not serious | Not serious | Undetected | ㊉㊉㊉◯  MODERATE | OR，0.87；  95%CI，0.82-0.92 | ＜0.001 |
| **HDL-C** **[S31]** | | | | | | | | | |
| 1 | Observational | Serious ^a^ | Not serious | Not serious | Not serious | Undetected | ㊉㊉㊉◯  MODERATE | OR，1.09；  95%CI，1.01-1.18 | 0.04 |
| **TG** **[S31]** | | | | | | | | | |
| 1 | Observational | Serious ^a^ | Not serious | Not serious | Not serious | Undetected | ㊉㊉㊉◯  MODERATE | OR，0.84；  95%CI，0.72-0.97 | 0.02 |
| **TC/HDL-C** **[S51]** | | | | | | | | | |
| 1 | Observational | Serious ^a^ | Not serious | Not serious | Not serious | Undetected | ㊉㊉㊉◯  MODERATE | OR，1.73；  95%CI，1.01-2.96 | 0.05 |
| **TG/HDL-C** **[S51]** | | | | | | | | | |
| 1 | Observational | Serious ^a^ | Not serious | Not serious | Not serious | Undetected | ㊉㊉㊉◯  MODERATE | OR，2.06；  95%CI，1.24-3.43 | 0.005 |
| **LDL-C/HDL-C** **[S51]** | | | | | | | | | |
| 1 | Observational | Serious ^a^ | Not serious | Not serious | Not serious | Undetected | ㊉㊉㊉◯  MODERATE | OR，1.93；  95%CI，1.07-3.50 | 0.03 |
| **White blood cell count** **[S78]** | | | | | | | | | |
| 1 | Observational | Serious ^a^ | Not serious | Not serious | Not serious | Undetected | ㊉㊉㊉◯  MODERATE | OR，1.10；  95%CI，1.01-1.19 | 0.02 |
| **AEC** **[S81]** | | | | | | | | | |
| 1 | Observational | Serious ^a^ | Not serious | Not serious | Not serious | Undetected | ㊉㊉㊉◯  MODERATE | OR，0.22；  95%CI，0.07-0.72 | 0.01 |
| **fT3** **[S26, S53]** | | | | | | | | | |
| 2 | Observational | Serious ^a^ | Not serious | Not serious | Not serious | Undetected | ㊉㊉㊉◯  MODERATE | OR，0.24；  95%CI，0.11-0.51 | ＜0.001 |
| **Albuminuria** **[S44, S49]** | | | | | | | | | |
| 2 | Observational | Serious ^a^ | Not serious | Not serious | Not serious | Undetected | ㊉㊉㊉◯  MODERATE | OR，1.66；  95%CI，1.20-2.28 | 0.002 |
| **FIB** **[S47, S73, S86, S88-89]** | | | | | | | | | |
| 5 | Observational | Serious ^a^ | Not serious | Not serious | Not serious | Undetected | ㊉㊉㊉◯  MODERATE | OR，6.64；  95%CI，3.40-12.97 | ＜0.001 |
| **FDP [S88]** | | | | | | | | | |
| 1 | Observational | Serious ^a^ | Not serious | Not serious | Not serious | Undetected | ㊉㊉㊉◯  MODERATE | OR，7.50；  95%CI，1.26-44.64 | 0.03 |
| **Globulin** **[S78]** | | | | | | | | | |
| 1 | Observational | Serious ^a^ | Not serious | Not serious | Not serious | Undetected | ㊉㊉㊉◯  MODERATE | OR，1.18；  95%CI，1.09-1.29 | ＜0.001 |
| **Caveolin [S52, S77]** | | | | | | | | | |
| 3 | Observational | Serious ^a^ | Not serious | Not serious | Not serious | Undetected | ㊉㊉㊉◯  MODERATE | OR，2.35；  95%CI，1.71-3.24 | ＜0.001 |
| **MMP9/TIMP** **[S63]** | | | | | | | | | |
| 2 | Observational | Serious ^a^ | Not serious | Not serious | Not serious | Undetected | ㊉㊉㊉◯  MODERATE | OR，1.72；  95%CI，1.31-2.28 | ＜0.001 |
| **S100B** **[S92]** | | | | | | | | | |
| 1 | Observational | Serious ^a^ | Not serious | Not serious | Not serious | Undetected | ㊉㊉㊉◯  MODERATE | OR，2.80；  95%CI，1.40-5.60 | 0.004 |
| **c-Fn** **[S94]** | | | | | | | | | |
| 1 | Observational | Serious ^a^ | Not serious | Not serious | Not serious | Undetected | ㊉㊉㊉◯  MODERATE | OR，2.10；  95%CI，1.30-3.39 | 0.002 |
| **GFR** **[S80, S105]** | | | | | | | | | |
| 3 | Observational | Serious ^a^ | Not serious | Not serious | Not serious | Undetected | ㊉㊉㊉◯  MODERATE | OR，1.83；  95%CI，1.38-2.43 | ＜0.001 |
| **Creatinine [S93]** | | | | | | | | | |
| 1 | Observational | Serious ^a^ | Not serious | Not serious | Not serious | Undetected | ㊉㊉㊉◯  MODERATE | OR，5.50；  95%CI，1.08-28.01 | 0.04 |
| **Homocysteine [S96]** | | | | | | | | | |
| 1 | Observational | Serious ^a^ | Not serious | Not serious | Not serious | Undetected | ㊉㊉㊉◯  MODERATE | OR，13.65；  95%CI，3.58-52.05 | ＜0.001 |
| **Apelin [S103]** | | | | | | | | | |
| 1 | Observational | Serious ^a^ | Not serious | Not serious | Not serious | Undetected | ㊉㊉㊉◯  MODERATE | OR，0.24；  95%CI，0.09-0.68 | 0.007 |
| **IL-1β [S103]** | | | | | | | | | |
| 1 | Observational | Serious ^a^ | Not serious | Not serious | Not serious | Undetected | ㊉㊉㊉◯  MODERATE | OR，1.06；  95%CI，1.03-1.09 | ＜0.001 |
| **IL-6 [S103]** | | | | | | | | | |
| 1 | Observational | Serious ^a^ | Not serious | Not serious | Not serious | Undetected | ㊉㊉㊉◯  MODERATE | OR，1.53；  95%CI，1.12-2.11 | 0.008 |
| **MDA [S103]** | | | | | | | | | |
| 1 | Observational | Serious ^a^ | Not serious | Not serious | Not serious | Undetected | ㊉㊉㊉◯  MODERATE | OR，2.49；  95%CI，1.32-4.70 | 0.005 |
| **SOD [S103]** | | | | | | | | | |
| 1 | Observational | Serious ^a^ | Not serious | Not serious | Not serious | Undetected | ㊉㊉㊉◯  MODERATE | OR，0.20；  95%CI，0.05-0.75 | 0.02 |
| **Early CT hypodensities [S23, S33]** | | | | | | | | | |
| 2 | Observational | Serious ^a^ | Not serious | Not serious | Not serious | Undetected | ㊉㊉㊉◯  MODERATE | OR，2.47；  95%CI，1.54-3.95 | ＜0.001 |
| **HDMCA sign** **[S12, S33, S48, S76, S83]** | | | | | | | | | |
| 5 | Observational | Serious ^a^ | Not serious | Not serious | Not serious | Undetected | ㊉㊉㊉◯  MODERATE | OR，1.57；  95%CI，1.09-2.25 | 0.01 |
| **Early infarct signs [S12, S32, S83]** | | | | | | | | | |
| 3 | Observational | Serious ^a^ | Not serious | Not serious | Not serious | Undetected | ㊉㊉㊉◯  MODERATE | OR，2.86；  95%CI，1.30-6.33 | 0.009 |
| **FLAIR hyperintensity [S25, S60]** | | | | | | | | | |
| 3 | Observational | Serious ^a^ | Not serious | Not serious | Not serious | Undetected | ㊉㊉㊉◯  MODERATE | OR，13.58；  95%CI，3.72-49.60 | ＜0.001 |
| **Early CT signs of cerebral ischaemia** **[S6, S46, S55]** | | | | | | | | | |
| 4 | Observational | Serious ^a^ | Not serious | Not serious | Not serious | Undetected | ㊉㊉㊉◯  MODERATE | OR，3.40；  95%CI，2.19-5.27 | ＜0.001 |
| **Brain infarction volume** **[S24, S35, S69]** | | | | | | | | | |
| 3 | Observational | Serious ^a^ | Not serious | Not serious | Not serious | Undetected | ㊉㊉㊉◯  MODERATE | OR，1.81；  95%CI，1.25-2.62 | 0.002 |
| **HPrs-PCT [S29]** | | | | | | | | | |
| 1 | Observational | Not serious | Not serious | Not serious | Serious ^c^ | Undetected | ㊉㊉㊉◯  MODERATE | OR，1.00；  95%CI，1.00-1.00 | 0.003 |
| **Supratentorial territory PCA** **[S37]** | | | | | | | | | |
| 1 | Observational | Serious ^a^ | Not serious | Not serious | Not serious | Undetected | ㊉㊉㊉◯  MODERATE | OR，4.31；  95%CI，1.20-15.49 | 0.03 |
| **CBV** **[S72]** | | | | | | | | | |
| 1 | Observational | Serious ^a^ | Not serious | Not serious | Not serious | Undetected | ㊉㊉㊉◯  MODERATE | OR，100.00；  95%CI，3.20-3125.31 | 0.009 |
| **CV-L [S66]** | | | | | | | | | |
| 1 | Observational | Serious ^a^ | Not serious | Not serious | Not serious | Undetected | ㊉㊉㊉◯  MODERATE | OR，1.50；  95%CI，1.14-1.98 | 0.004 |
| **MMP-9-1562C/T polymorphism genotypes** **[S41]** | | | | | | | | | |
| 1 | Observational | Serious ^a^ | Not serious | Not serious | Not serious | Undetected | ㊉㊉㊉◯  MODERATE | OR，13.08；  95%CI，1.04-164.51 | 0.05 |
| **PAI-1 5G/5G genotype** **[S87]** | | | | | | | | | |
| 1 | Observational | Serious ^a^ | Not serious | Not serious | Not serious | Undetected | ㊉㊉㊉◯  MODERATE | OR，4.75；  95%CI，1.18-19.12 | 0.03 |
| **Rs1801020, C allele** **[S4]** | | | | | | | | | |
| 1 | Observational | Serious ^a^ | Not serious | Not serious | Not serious | Undetected | ㊉㊉㊉◯  MODERATE | OR，2.04；  95%CI，1.38-3.01 | ＜0.001 |
| **Rs669, A allele** **[S4]** | | | | | | | | | |
| 1 | Observational | Serious ^a^ | Not serious | Not serious | Not serious | Undetected | ㊉㊉㊉◯  MODERATE | OR，2.19；  95%CI，1.57-3.06 | ＜0.001 |
| **Antithrombotic therapy** **[S2, S8-10, S12, S19, S21, S23, S32, S42, S46-47, S64, S75, S79, S84]** | | | | | | | | | |
| 22 | Observational | Serious ^a^ | Not serious | Not serious | Not serious | Undetected | ㊉㊉㊉◯  MODERATE | OR，2.28；  95%CI，1.81-2.87 | ＜0.001 |
| **Thrombolytic therapy** **[S2, S21, S23, S42, S46, S75, S84]** | | | | | | | | | |
| 8 | Observational | Serious ^a^ | Not serious | Not serious | Not serious | Undetected | ㊉㊉㊉◯  MODERATE | OR，2.00；  95%CI，1.36-2.94 | ＜0.001 |
| **Antiplatelet therapy [S8-10, S12, S32, S47, S64]** | | | | | | | | | |
| 10 | Observational | Serious ^a^ | Not serious | Not serious | Not serious | Undetected | ㊉㊉㊉◯  MODERATE | OR，2.15；  95%CI，1.70-2.72 | ＜0.001 |
| **Anticoagulant therapy** **[S12, S47, S79]** | | | | | | | | | |
| 3 | Observational | Serious ^a^ | Not serious | Not serious | Not serious | Undetected | ㊉㊉㊉◯  MODERATE | OR，4.40；  95%CI，1.38-14.01 | 0.01 |
| **Antihypertensive drugs** **[S8, S36]** | | | | | | | | | |
| 2 | Observational | Serious ^a^ | Not serious | Not serious | Not serious | Undetected | ㊉㊉㊉◯  MODERATE | OR，1.63；  95%CI，1.24-2.16 | ＜0.001 |
| **Lipid-lowering drugs** **[S17, S82, S99]** | | | | | | | | | |
| 3 | Observational | Serious ^a^ | Not serious | Not serious | Not serious | Undetected | ㊉㊉㊉◯  MODERATE | OR，3.23；  95%CI，2.33-4.48 | ＜0.001 |
| **Microcatheter injection (MCI)** **[S34]** | | | | | | | | | |
| 1 | Observational | Serious ^a^ | Not serious | Not serious | Not serious | Undetected | ㊉㊉㊉◯  MODERATE | OR，3.60；  95%CI，1.12-11.57 | 0.03 |
| **Additional endovascular therapy** **[S37]** | | | | | | | | | |
| 1 | Observational | Serious ^a^ | Not serious | Not serious | Not serious | Undetected | ㊉㊉㊉◯  MODERATE | OR，8.71；  95%CI，2.54-29.89 | ＜0.001 |
| **Deviation from the protocol** **[S55]** | | | | | | | | | |
| 1 | Observational | Serious ^a^ | Not serious | Not serious | Not serious | Undetected | ㊉㊉㊉◯  MODERATE | OR，11.10；  95%CI，2.40-51.34 | 0.002 |
| **Periventricular transit time to the peak (TTP) [S69]** | | | | | | | | | |
| 1 | Observational | Serious ^a^ | Not serious | Not serious | Not serious | Undetected | ㊉㊉㊉◯  MODERATE | OR，4.74；  95%CI，1.62-13.83 | 0.004 |
| **Vaspin** **[S103]** | | | | | | | | | |
| 1 | Observational | Serious ^a^ | Not serious | Not serious | Not serious | Undetected | ㊉㊉㊉◯  MODERATE | OR，0.26；  95%CI，0.12-0.58 | 0.001 |
| **Age & NIHSS** **[S11, S19]** | | | | | | | | | |
| 2 | Observational | Serious ^a^ | Not serious | Not serious | Not serious | Undetected | ㊉㊉㊉◯  MODERATE | OR，4.08；  95%CI，2.69-6.18 | ＜0.001 |
| **Age & hypertension [S19]** | | | | | | | | | |
| 1 | Observational | Serious ^a^ | Not serious | Not serious | Not serious | Undetected | ㊉㊉㊉◯  MODERATE | OR，2.10；  95%CI，1.02-4.31 | 0.04 |
| **Age & DBP [S19]** | | | | | | | | | |
| 1 | Observational | Serious ^a^ | Not serious | Not serious | Not serious | Undetected | ㊉㊉㊉◯  MODERATE | OR，6.10；  95%CI，2.30-16.18 | ＜0.001 |
| **Body weight** **[S9-10, S21, S72, S75, S79, S85]** | | | | | | | | | |
| 7 | Observational | Serious ^a^ | Not serious | Not serious | Serious ^c^ | Undetected | ㊉㊉◯◯  LOW | OR，1.26；  95%CI，0.96-1.67 | 0.1 |
| **Sex** **[S5, S56, S65, S75, S105]** | | | | | | | | | |
| 5 | Observational | Serious ^a^ | Not serious | Not serious | Serious ^c^ | Undetected | ㊉㊉◯◯  LOW | OR，0.96；  95%CI，0.63-1.47 | 0.85 |
| **Stroke** **[S7, S10, S38, S47, S56, S58, S61, S75, S100, S105]** | | | | | | | | | |
| 10 | Observational | Serious ^a^ | Not serious | Serious ^b^ | Not serious | Undetected | ㊉㊉◯◯  LOW | OR，4.68；  95%CI，1.49-14.70 | 0.008 |
| **Dyslipidemia** **[S19, S74, S87]** | | | | | | | | | |
| **3** | Observational | Serious ^a^ | Not serious | Not serious | Serious ^c^ | Undetected | ㊉㊉◯◯  LOW | OR，1.18；  95%CI，0.57-2.47 | 0.65 |
| **Visual field deficits** **[S33]** | | | | | | | | | |
| 1 | Observational | Serious ^a^ | Not serious | Not serious | Serious ^c^ | Undetected | ㊉㊉◯◯  LOW | OR，1.07；  95%CI，0.29-3.91 | 0.92 |
| **MPV** **[S50]** | | | | | | | | | |
| 1 | Observational | Serious ^a^ | Not serious | Not serious | Serious ^c^ | Undetected | ㊉㊉◯◯  LOW | OR，1.02；  95%CI，1.00-1.04 | 0.02 |
| **PTA [S78]** | | | | | | | | | |
| 1 | Observational | Serious ^a^ | Not serious | Not serious | Serious ^c^ | Undetected | ㊉㊉◯◯  LOW | OR，1.02；  95%CI，1.00-1.03 | 0.02 |
| **NLR** **[S22, S104]** | | | | | | | | | |
| 2 | Observational | Serious ^a^ | Not serious | Not serious | Serious ^c^ | Undetected | ㊉㊉◯◯  LOW | OR，1.09；  95%CI，1.00-1.18 | 0.04 |
| **Platelet count** **[S6, S8, S15, S18, S28-29, S36, S86, S90]** | | | | | | | | | |
| 9 | Observational | Serious ^a^ | Not serious | Not serious | Serious ^c^ | Undetected | ㊉㊉◯◯  LOW | OR，1.00；  95%CI，0.98-1.01 | 0.86 |
| **UA** **[S50]** | | | | | | | | | |
| 1 | Observational | Serious ^a^ | Not serious | Not serious | Serious ^c^ | Undetected | ㊉㊉◯◯  LOW | OR，1.00；  95%CI，0.99-1.00 | 0.05 |
| **DBP** **[S4, S85]** | | | | | | | | | |
| 2 | Observational | Serious ^a^ | Not serious | Not serious | Serious ^c^ | Undetected | ㊉㊉◯◯  LOW | OR，1.43；  95%CI，0.89-2.31 | 0.14 |
| **Albumin** **[S50, S62, S95]** | | | | | | | | | |
| 3 | Observational | Serious ^a^ | Not serious | Not serious | Serious ^c^ | Undetected | ㊉㊉◯◯  LOW | OR，2.30；  95%CI，0.89-6.00 | 0.09 |
| **WMH** **[S45, S68]** | | | | | | | | | |
| 2 | Observational | Serious ^a^ | Not serious | Not serious | Serious ^c^ | Undetected | ㊉㊉◯◯  LOW | OR，2.45；  95%CI，0.95-6.32 | 0.06 |
| **Time from onset to treatment (OTT) [S4, S9, S12, S20, S90]** | | | | | | | | | |
| 5 | Observational | Serious ^a^ | Not serious | Not serious | Serious ^c^ | Undetected | ㊉㊉◯◯  LOW | OR，1.06；  95%CI，0.99-1.15 | 0.1 |
| **Age & body weight [S19]** | | | | | | | | | |
| 1 | Observational | Serious ^a^ | Not serious | Not serious | Serious ^c^ | Undetected | ㊉㊉◯◯  LOW | OR，2.40；  95%CI，0.90-6.40 | 0.08 |
| **ADC** **[S40, S42]** | | | | | | | | | |
| 2 | Observational | Serious ^a^ | Not serious | Serious ^b^ | Serious ^c^ | Undetected | ㊉◯◯◯  VERY LOW | OR，2.72；  95%CI，0.43-17.14 | 0.29 |
| **Smoke** **[S77, S86]** | | | | | | | | | |
| 2 | Observational | Serious ^a^ | Not serious | Serious ^b^ | Serious ^c^ | Undetected | ㊉◯◯◯  VERY LOW | OR，0.47；  95%CI，0.02-14.61 | 0.67 |
| **HbA1c** **[S27, S62]** | | | | | | | | | |
| 2 | Observational | Serious ^a^ | Not serious | Serious ^b^ | Serious ^c^ | Undetected | ㊉◯◯◯  VERY LOW | OR，3.36；  95%CI，0.50-22.56 | 0.21 |
| **MAP** **[S32, S47, S73]** | | | | | | | | | |
| 3 | Observational | Serious ^a^ | Not serious | Serious ^b^ | Serious ^c^ | Undetected | ㊉◯◯◯  VERY LOW | OR，3.68；  95%CI，0.61-22.13 | 0.15 |
| **Time to recanalization [S18, S43]** | | | | | | | | | |
| 2 | Observational | Serious ^a^ | Not serious | Serious ^b^ | Serious ^c^ | Undetected | ㊉◯◯◯  VERY LOW | OR，3.19；  95%CI，0.18-55.75 | 0.43 |

**GRADE Working Group grades of evidence**

High certainty: we are very confident that the true effect lies close to that of the estimate of the effect.

Moderate certainty: we are moderately confident in the effect estimate: the true effect is likely to be close to the estimate of the effect, but there is a possibility that it is substantially different.

Low certainty: our confidence in the effect estimate is limited: the true effect may be substantially different from the estimate of the effect.

Very low certainty: we have very little confidence in the effect estimate: the true effect is likely to be substantially different from the estimate of effect.

**Explanations**

a. Risk of bias: The retrospective enrollment of patients may have introduced classification bias, certainty in evidence was downgraded for risk of bias.

b. Certainty in evidence was downgraded for high heterogeneity.

c. Imprecise: Certainty in evidence was downgraded for imprecision, given that the 95% CI suggests that there may be no association.

**Table 5. Classification for bleeding-related risk factors**

| **Factor categories** | **Certainty of evidence** | **Treatable factors** | **Non-treatable factors** |
| --- | --- | --- | --- |
| Demographic factors | Moderate | / | Age; Race |
| Functional factors | Moderate | CHADS2 score >2; Higher SEDAN score; Arterial stiffness index (ASI) | Alberta Stroke Programme Early CT Score (ASPECTS) ≤7; National Institutes of Health stroke scale (NIHSS); modified Rankin scale (mRS); Thrombolysis in Cerebral Infarction (TICI) score (3, 2, 1, 0; Each one decrease); Low ejection fraction (EF); K ^trans^ (The contrast volume transfer coefficient) |
| Medical illness and patient history factors | Moderate | Peripheral vascular disease (PVD); Cerebral small vascular diseases (CSVD); Cerebral microbleeds; Leukoaraiosis  Cardiovascular disease; Prior myocardial infarction; Valvular heart diseases; Hypertension; AF; Congestive heart failure; Diabetes | Poor collaterals; Recent facial or head trauma (2 weeks); Cerebral artery occlusion; Decreased levels of consciousness |
|  | Low | Stroke | / |
| Laboratory and physical examination factors | High | / | Thrombin-activated fibrinolysis inhibitor (TAFI); Plasminogen activator inhibitor (PAI)-1; Activated protein C (APC) |
|  | Moderate | Blood sugar; Blood pressure; Systolic blood pressure (SBP); Pulse pressure; International normalized ratio (INR); Activated partial thromboplastin time (APTT)  Total cholesterol (TC); Low density lipoprotein cholesterol (LDL-C); High density lipoprotein cholesterol (HDL-C); Triglyceride (TG); TC/HDL-C; TG/HDL-C; LDL-C/HDL-C; White blood cell count; Absolute eosinophil count (AEC); Low serum-free triiodothyronine (fT3)  Albuminuria; Fibrinogen (FIB); Fibrinogen degradation products (FDP); Globulin; Glomerular filtration rate (GFR); Creatinine; Homocysteine | Platelet derived growth factor-CC (PDGF-CC)  MMP-9-1562C/T polymorphism genotypes; PAI-1 5G/5G genotype; rs1801020, C allele; rs669, A allele  Caveolin; Matrix metalloproteinase-9 (MMP9) / tissue inhibitor of metalloproteinases (TIMP); S100B; Cellular fibronectin (c-Fn)  Apelin; Interleukin-1β (IL-1β); Interleukin-6 (IL-6); Malondialdehyde (MDA); Superoxide dismutase (SOD)  Early computed tomography (CT) hypodensities; Hyperdense middle cerebral artery (HDMCA) sign; Early infarct signs; Fluid-attenuated inversion recovery (FLAIR) hyperintensity; Early CT signs of cerebral ischaemia; Brain infarction volume; High-permeability region size on PCT (HPrs-PCT); Supratentorial territory of the posterior cerebral artery (PCA); Cerebral blood volume (CBV); Calcification volume on the lesion side (CV-L) |
|  | Low | Mean platelet volume (MPV); Prothrombin time activity percentage (PTA); Neutrophil to lymphocyte ratio (NLR) | / |
| Medication factors | Moderate | Antithrombotic therapy; Thrombolytic therapy; Antiplatelet therapy; Anticoagulant therapy  Antihypertensive drugs; Lipid-lowering drugs; Microcatheter injection (MCI); Additional endovascular therapy; Deviation from the protocol | Periventricular transit time to the peak (TTP); Vaspin |
| Other factors | Moderate | Age & hypertension; Age & DBP | Age & NIHSS |

**Supplemental Figures (sFigures): Forest plots showing the association between risk factors and ICH (From sFigure 1-110)**

**sFigure 1: Forest plot showing the association between age and ICH**

**
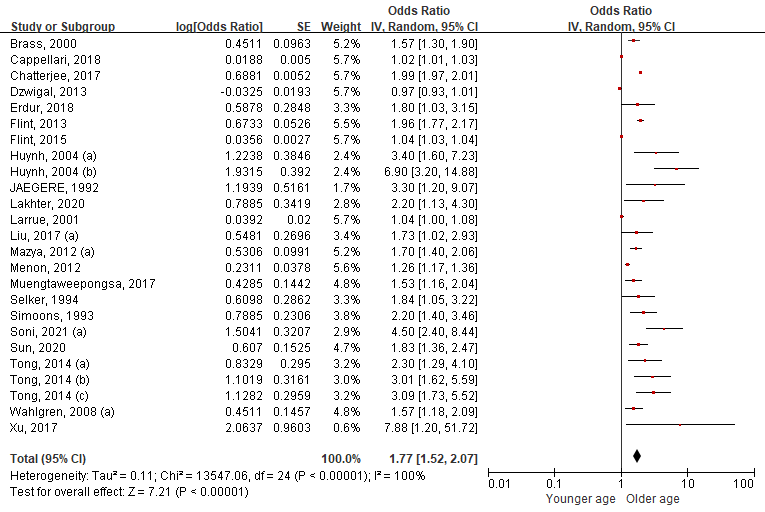
**

**sFigure 2: Forest plot showing the association between race and ICH
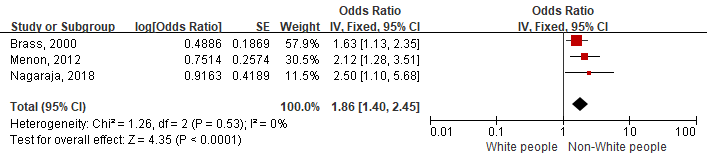
**

**sFigure 3: Forest plot showing the association between body weight and ICH
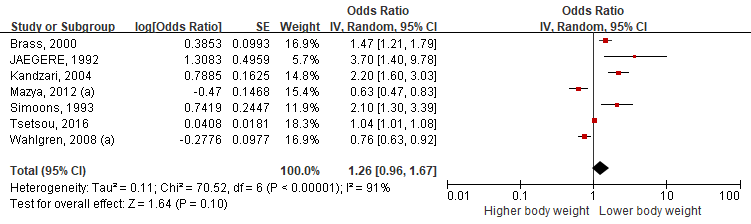
**

**sFigure 4: Forest plot showing the association between sex and ICH
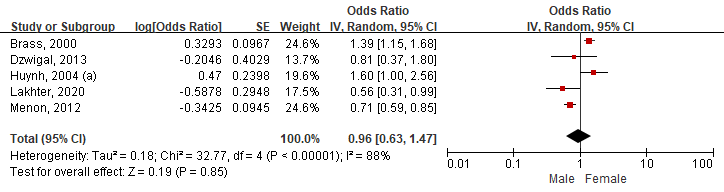
**

**sFigure 5: Forest plot showing the association between ASPECT and ICH**

**
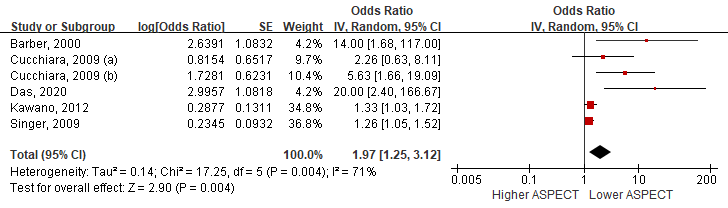
**

**sFigure 6: Forest plot showing the association between NIHSS and ICH**

**
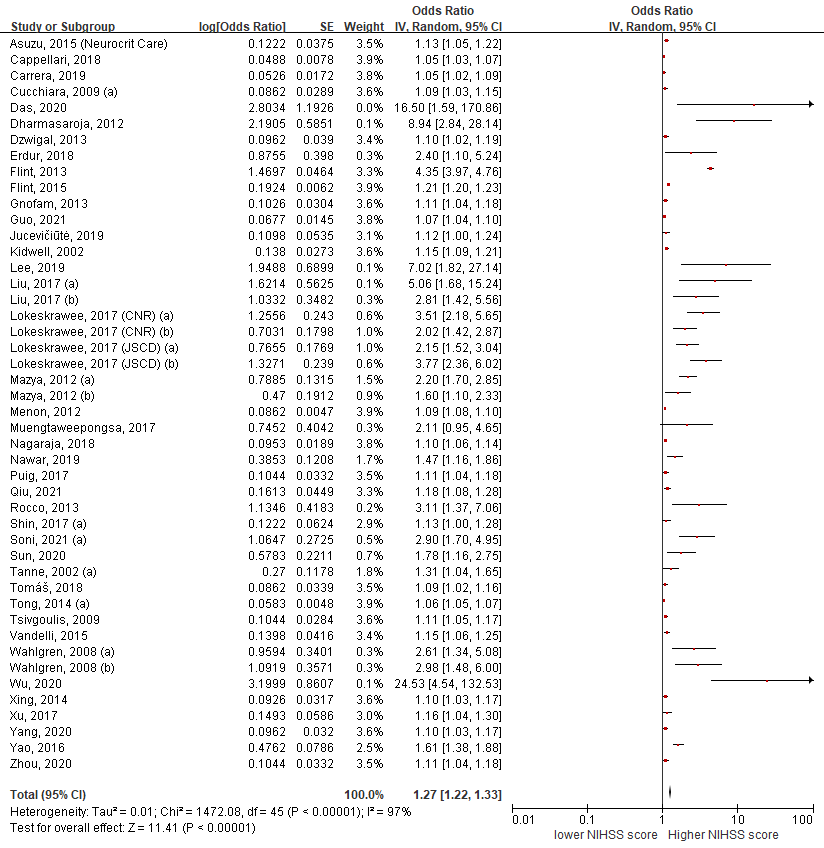
**

**sFigure 7: Forest plot showing the association between mRS and ICH**

**
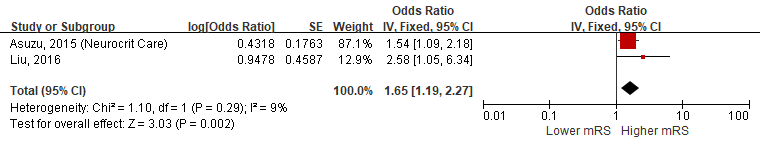
**

**sFigure 8: Forest plot showing the association between TICI score and ICH**

**
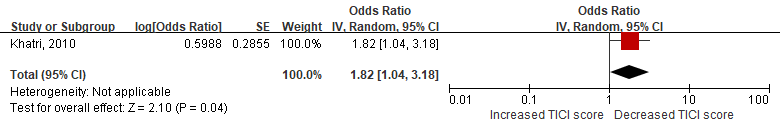
**

**sFigure 9: Forest plot showing the association between CHADS2 score and ICH**

**
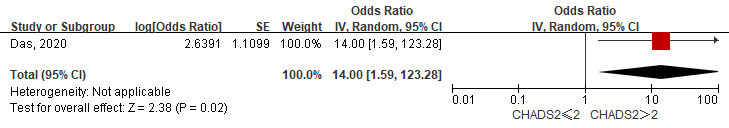
**

**sFigure 10: Forest plot showing the association between low EF and ICH**

**
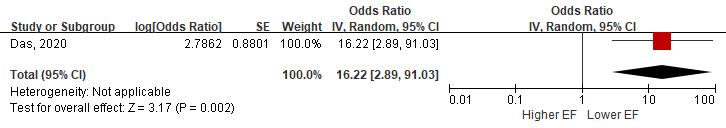
**

**sFigure 11: Forest plot showing the association between SEDAN score and ICH**

**
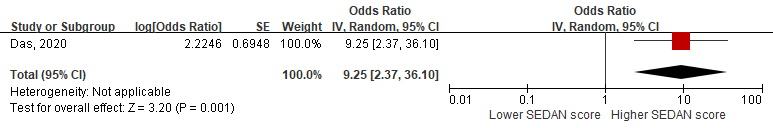
**

**sFigure 12: Forest plot showing the association between arterial stiffness index (ASI) and ICH**

**
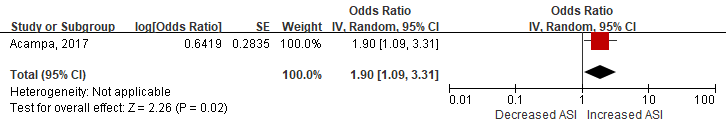
**

**sFigure 13: Forest plot showing the association between K ^trans^ and ICH**

**
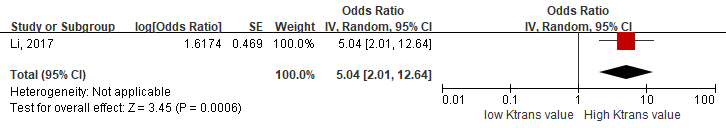
**

**sFigure 14: Forest plot showing the association between ADC and ICH**

**
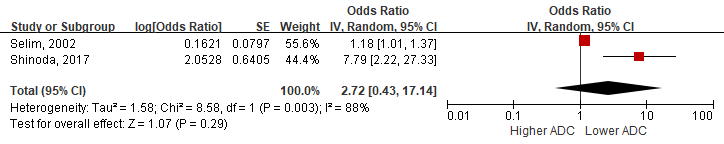
**

**sFigure 15: Forest plot showing the association between peripheral vascular disease (PVD) and ICH
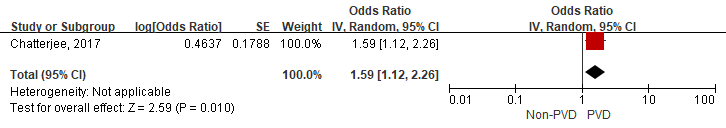
**

**sFigure 16: Forest plot showing the association between cerebral small vascular diseases (CSVD) and ICH**

**
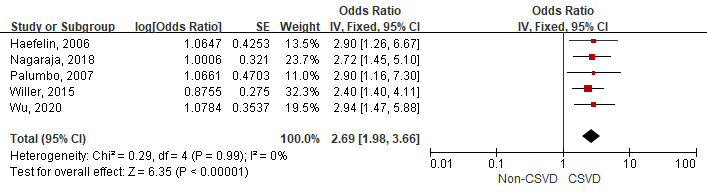
**

**sFigure 17: Forest plot showing the association between cerebral microbleeds and ICH**

**
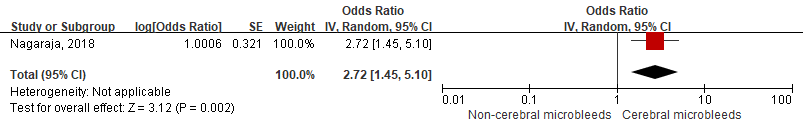
**

**sFigure 18: Forest plot showing the association between leukoaraiosis and ICH**

**
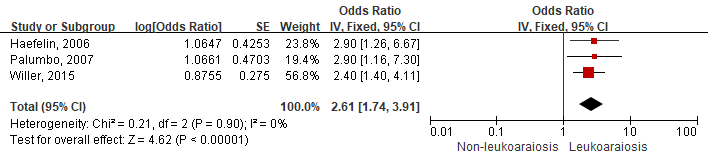
**

**sFigure 19: Forest plot showing the association between poor collaterals and ICH**

**
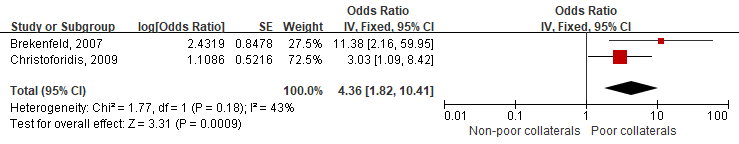
**

**sFigure 20: Forest plot showing the association between recent facial or head trauma and ICH**

**
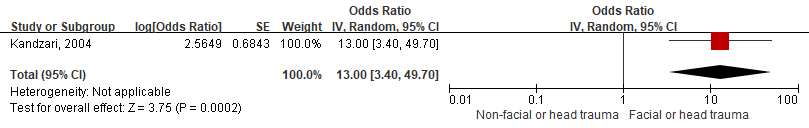
**

**sFigure 21: Forest plot showing the association between cerebral artery occlusion and ICH
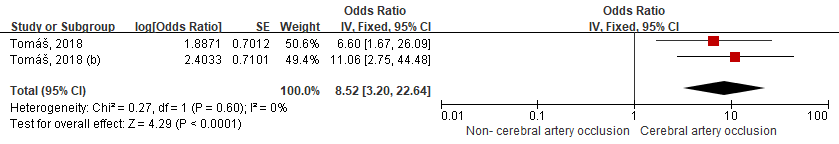
**

**sFigure 22: Forest plot showing the association between decreased levels of consciousness and ICH**

**
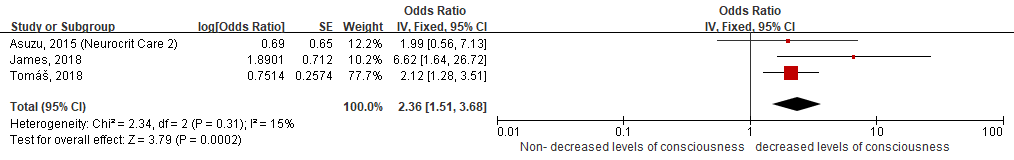
**

**sFigure 23: Forest plot showing the association between stroke and ICH**

**
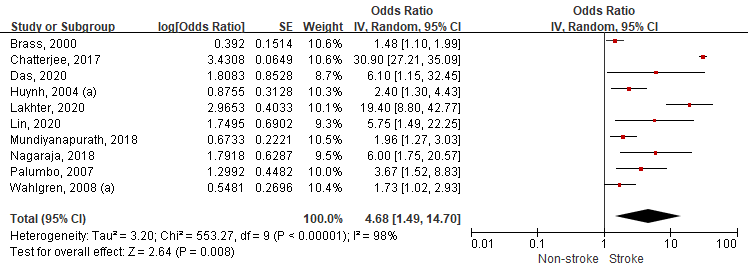
**

**sFigure 24: Forest plot showing the association between cardiovascular disease and ICH**

**
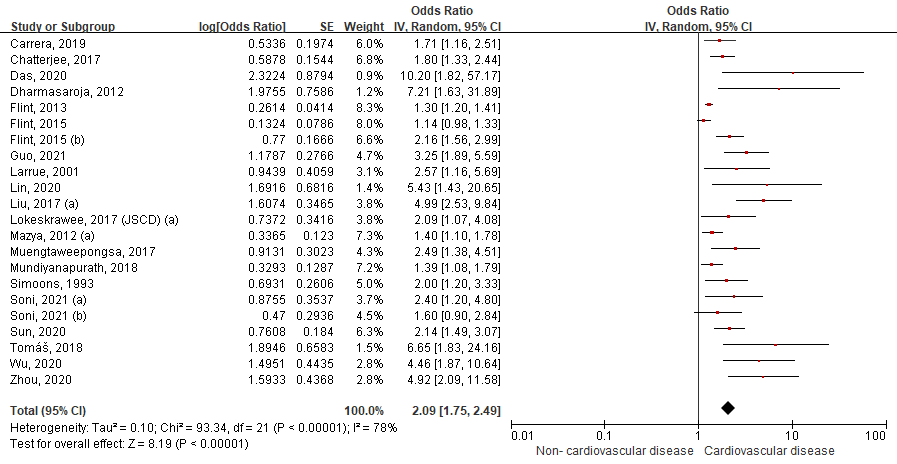
**

**sFigure 25: Forest plot showing the association between prior myocardial infarction and ICH**

**
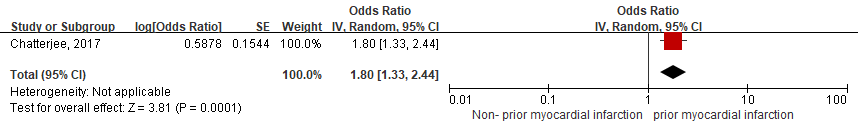
**

**sFigure 26: Forest plot showing the association between valvular heart diseases and ICH**

**
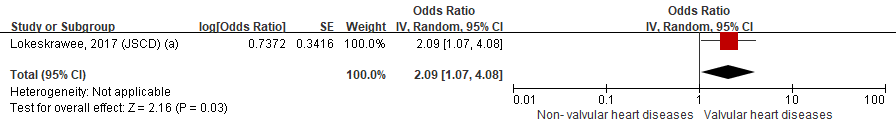
**

**sFigure 27: Forest plot showing the association between hypertension and ICH**

**
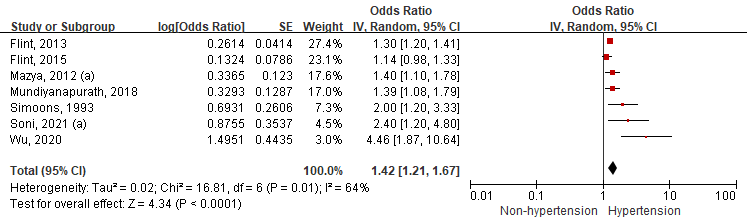
**

**sFigure 28: Forest plot showing the association between AF and ICH**

**
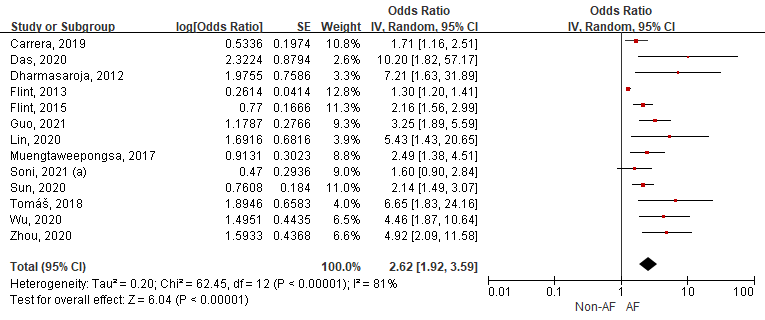
**

**sFigure 29: Forest plot showing the association between congestive heart failure and ICH**

**
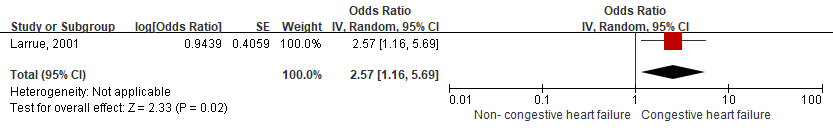
**

**sFigure 30: Forest plot showing the association between diabetes and ICH**

**
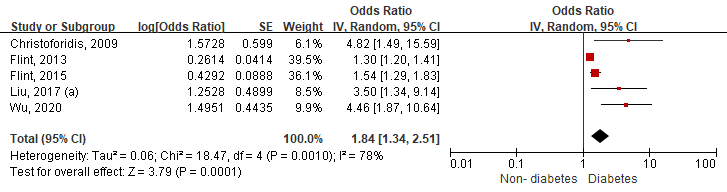
**

**sFigure 31: Forest plot showing the association between dyslipidemia and ICH**

**
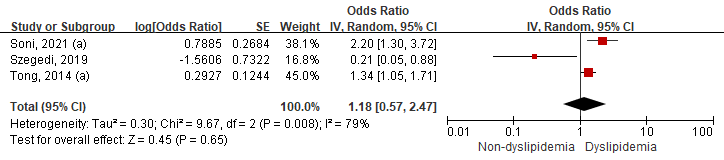
**

**sFigure 32: Forest plot showing the association between visual field deficits and ICH
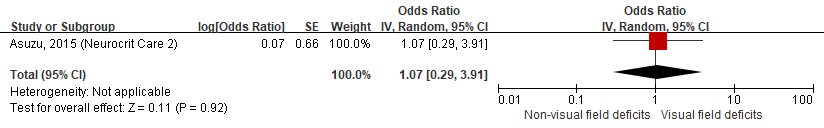
**

**sFigure 33: Forest plot showing the association between smoke and ICH**

**
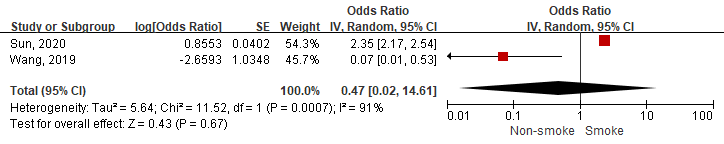
**

**sFigure 34: Forest plot showing the association between TAFI and ICH**

**
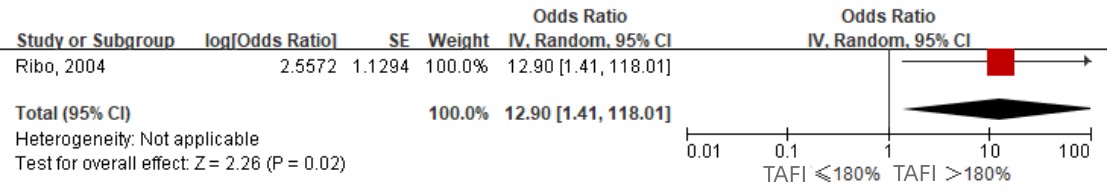
**

**sFigure 35: Forest plot showing the association between PAI-1 and ICH**

**
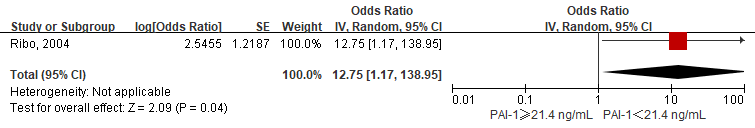
**

**sFigure 36: Forest plot showing the association between blood sugar and ICH**

**
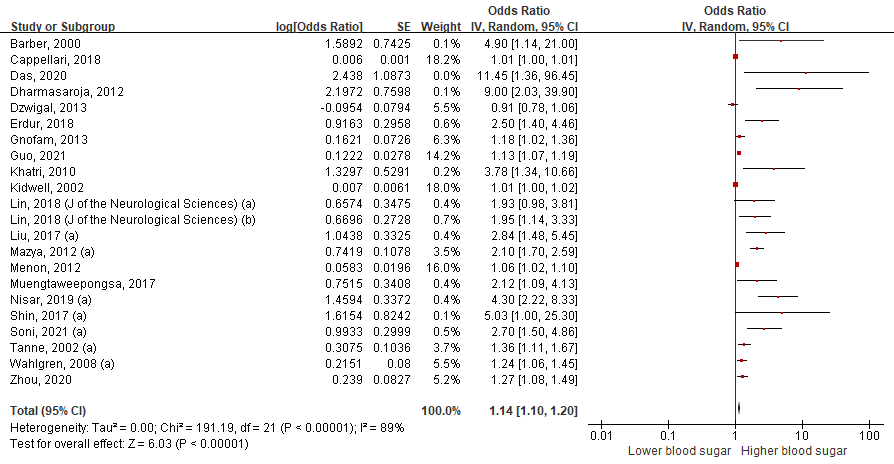
**

**sFigure 37: Forest plot showing the association between PDGF-CC and ICH**

**
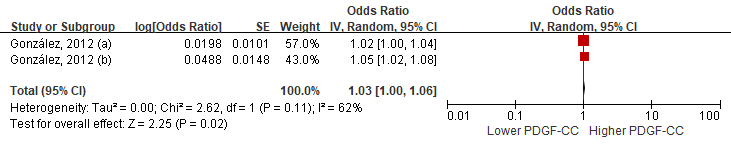
**

**sFigure 38: Forest plot showing the association between BP protocol violations and ICH**

**
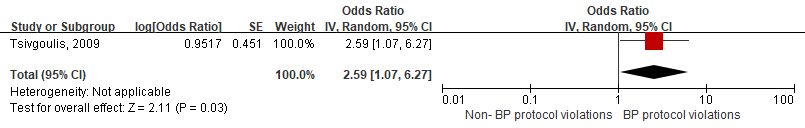
**

**sFigure 39: Forest plot showing the association between SBP and ICH**

**
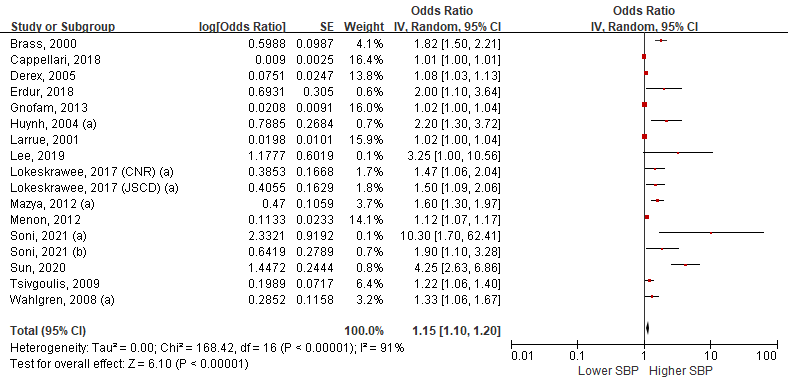
**

**sFigure 40: Forest plot showing the association between pulse pressure and ICH**

**
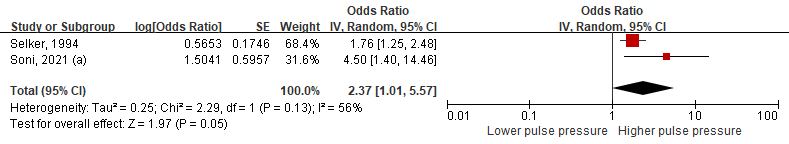
**

**sFigure 41: Forest plot showing the association between INR and ICH**

**
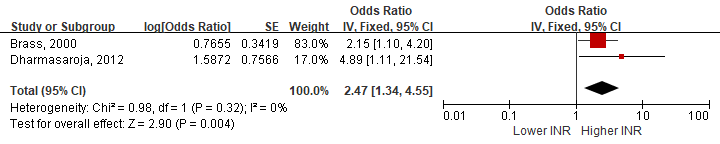
**

**sFigure 42: Forest plot showing the association between APTT and ICH**

**
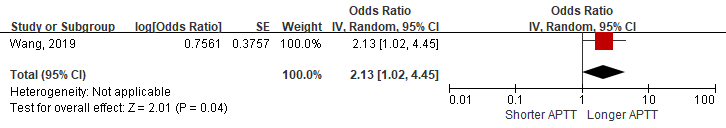
**

**sFigure 43: Forest plot showing the association between MPV and ICH**

**
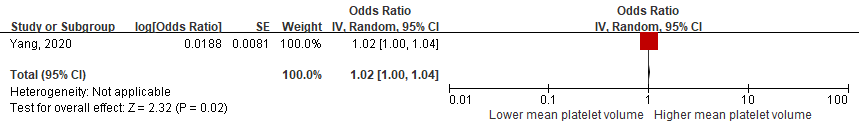
**

**sFigure 44: Forest plot showing the association between PTA and ICH**

**
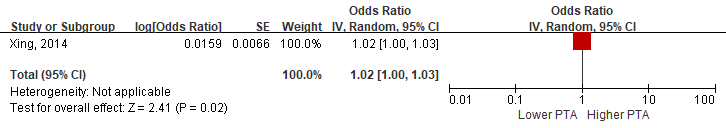
**

**sFigure 45: Forest plot showing the association between TC and ICH**

**
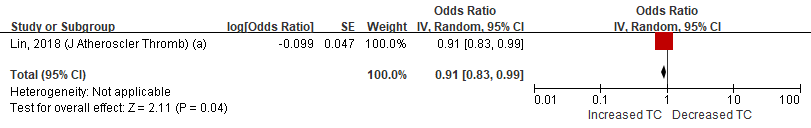
**

**sFigure 46: Forest plot showing the association between LDL-C and ICH**

**
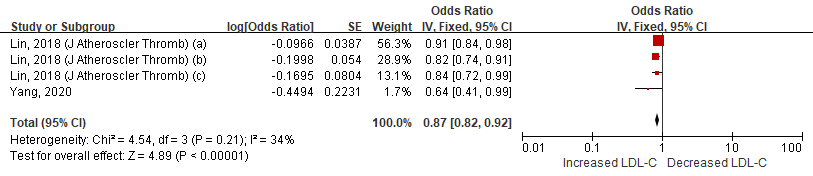
**

**sFigure 47: Forest plot showing the association between HDL-C and ICH**

**
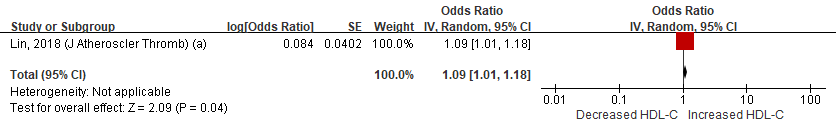
**

**sFigure 48: Forest plot showing the association between TG and ICH**

**
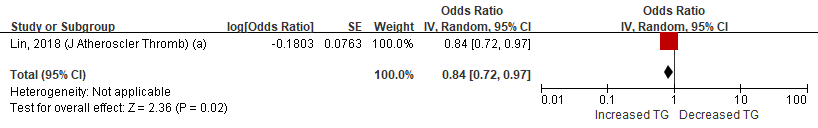
**

**sFigure 49: Forest plot showing the association between TC/HDL-C and ICH**

**
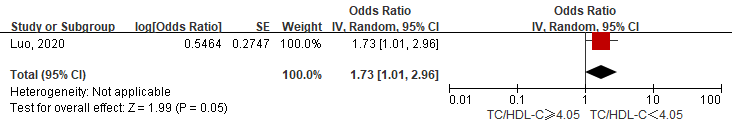
**

**sFigure 50: Forest plot showing the association between TG/HDL-C and ICH**

**
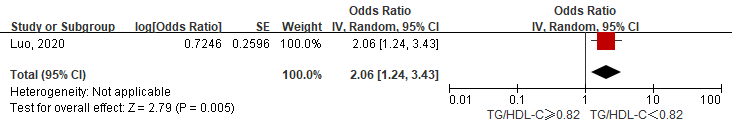
**

**sFigure 51: Forest plot showing the association between LDL-C/HDL-C and ICH**

**
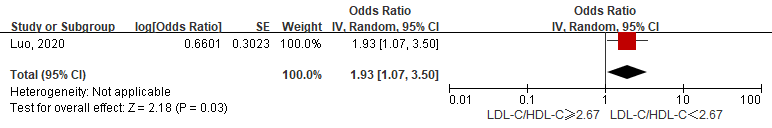
**

**sFigure 52: Forest plot showing the association between white blood cell count and ICH**

**
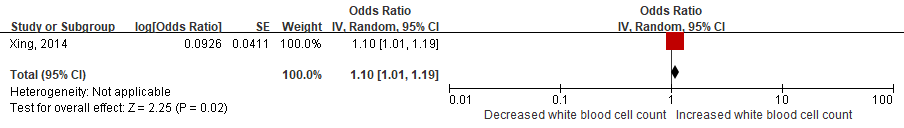
**

**sFigure 53: Forest plot showing the association between AEC and ICH**

**
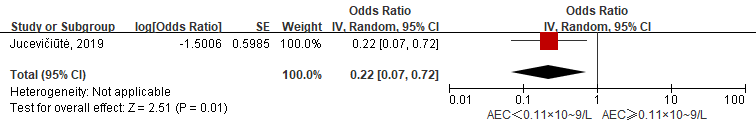
**

**sFigure 54: Forest plot showing the association between fT3 and ICH**

**
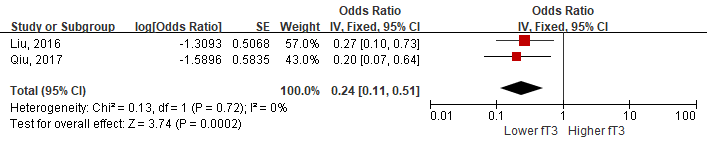
**

**sFigure 55: Forest plot showing the association between NLR and ICH**

**
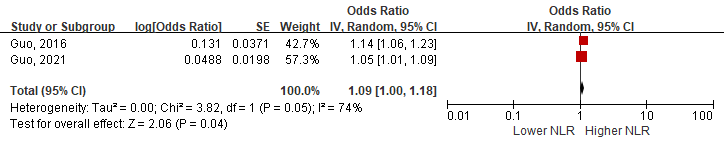
**

**sFigure 56: Forest plot showing the association between APC and ICH**

**
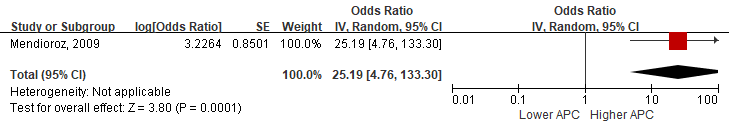
**

**sFigure 57: Forest plot showing the association between albuminuria and ICH**

**
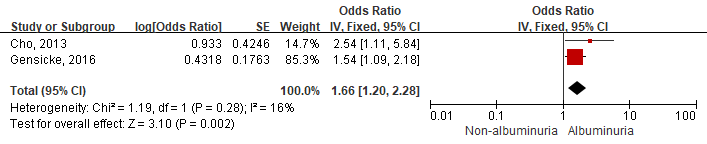
**

**sFigure 58: Forest plot showing the association between FIB and ICH**

**
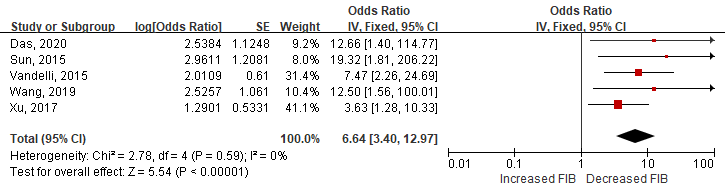
**

**sFigure 59: Forest plot showing the association between FDP and ICH**

**
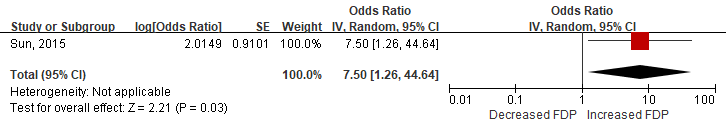
**

**sFigure 60: Forest plot showing the association between globulin and ICH**

**
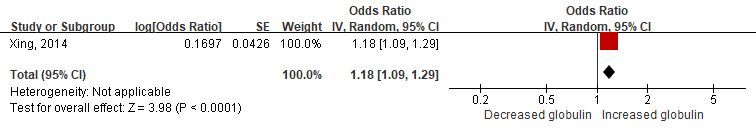
**

**sFigure 61: Forest plot showing the association between caveolin and ICH**

**
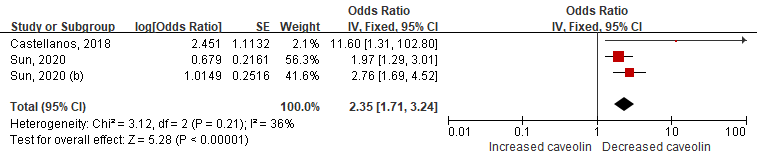
**

**sFigure 62: Forest plot showing the association between MMP9/TIMP and ICH**

**
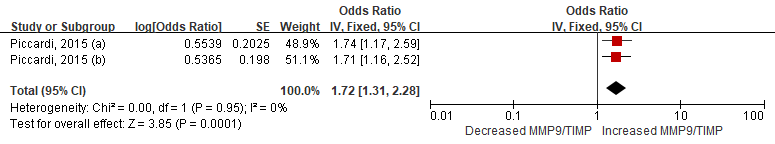
**

**sFigure 63: Forest plot showing the association between S100B and ICH**

**
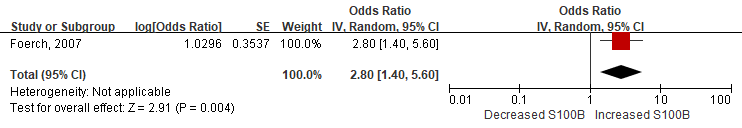
**

**sFigure 64: Forest plot showing the association between c-Fn and ICH**

**
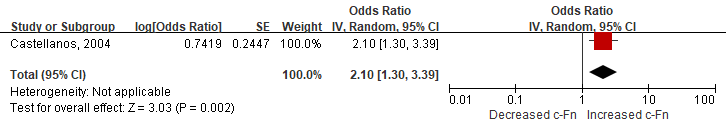
**

**sFigure 65: Forest plot showing the association between GFR and ICH**

**
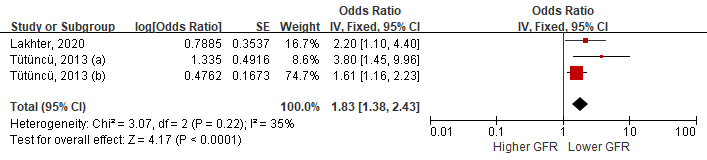
**

**sFigure 66: Forest plot showing the association between creatinine and ICH**

**
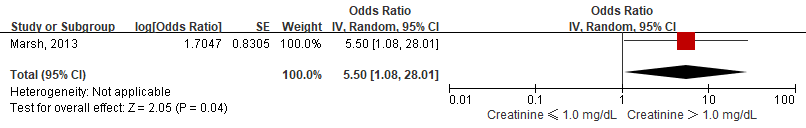
**

**sFigure 67: Forest plot showing the association between homocysteine and ICH**

**
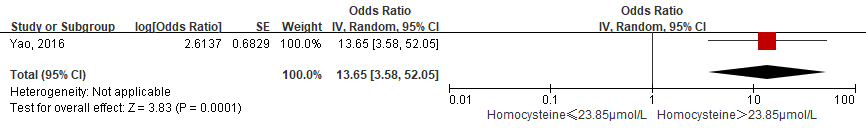
**

**sFigure 68: Forest plot showing the association between apelin and ICH**

**
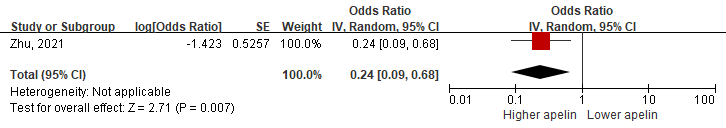
**

**sFigure 69: Forest plot showing the association between IL-1β and ICH**

**
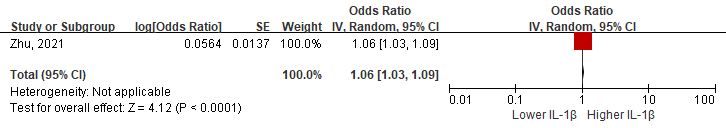
**

**sFigure 70: Forest plot showing the association between IL-6 and ICH**

**
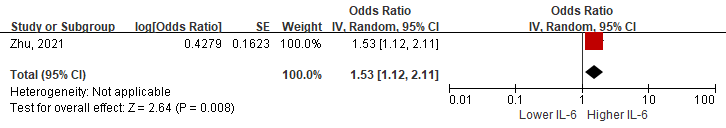
**

**sFigure 71: Forest plot showing the association between MDA and ICH**

**
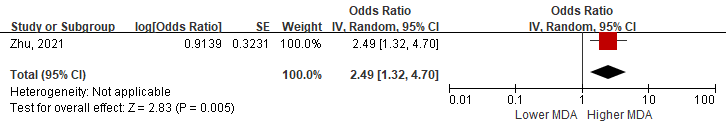
**

**sFigure 72: Forest plot showing the association between SOD and ICH**

**
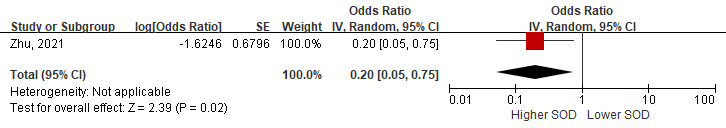
**

**sFigure 73: Forest plot showing the association between platelet count and ICH**

**
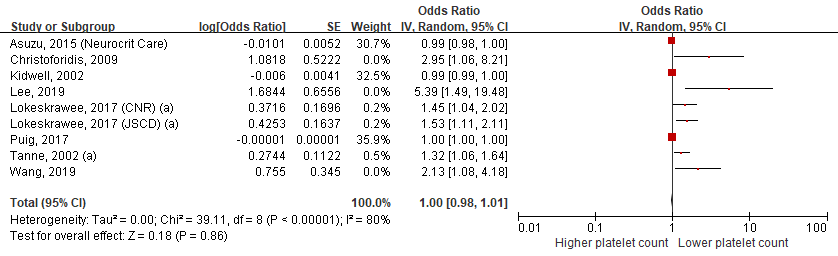
**

**sFigure 74: Forest plot showing the association between UA and ICH**

**
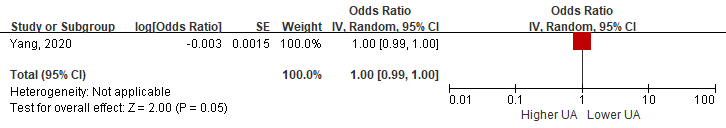
**

**sFigure 75: Forest plot showing the association between DBP and ICH**

**
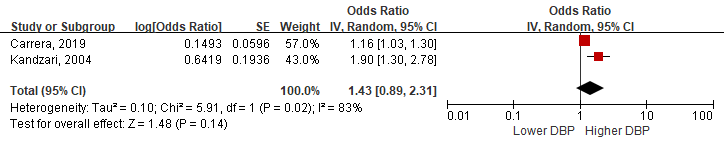
**

**sFigure 76: Forest plot showing the association between albumin and ICH**

**
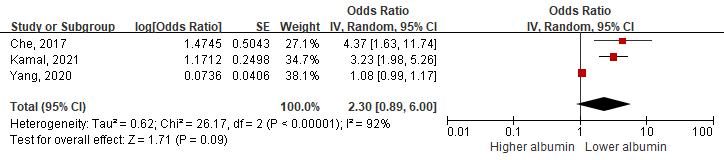
**

**sFigure 77: Forest plot showing the association between HbA1c and ICH**

**
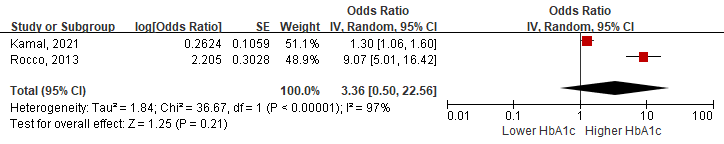
**

**sFigure 78: Forest plot showing the association between MAP and ICH**

**
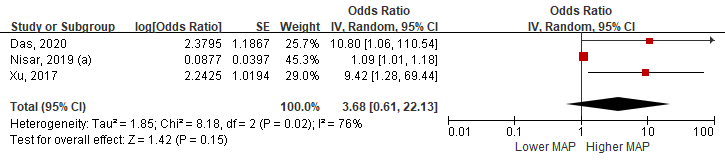
**

**sFigure 79: Forest plot showing the association between early CT hypodensities and ICH**

**
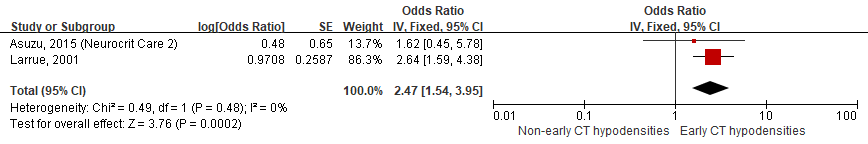
**

**sFigure 80: Forest plot showing the association between HDMCA sign and ICH**

**
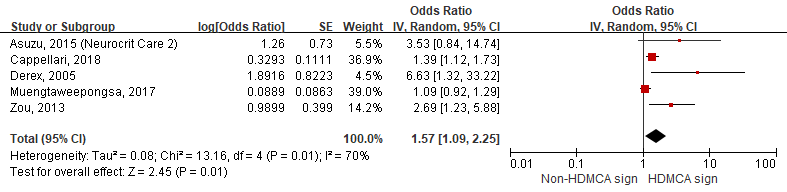
**

**sFigure 81: Forest plot showing the association between early infarct signs and ICH**

**
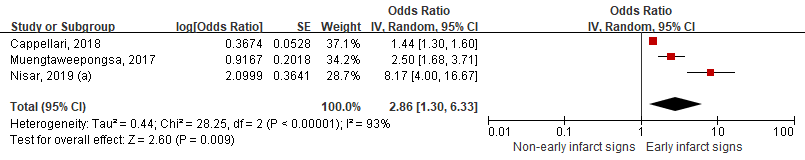
**

**sFigure 82: Forest plot showing the association between FLAIR hyperintensity and ICH**

**
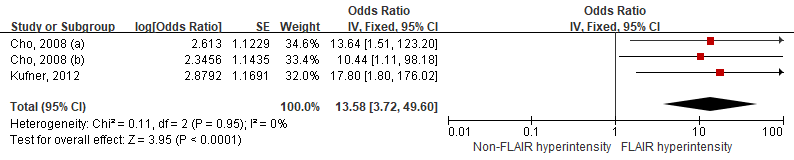
**

**sFigure 83: Forest plot showing the association between early CT signs of cerebral ischaemia and ICH**

**
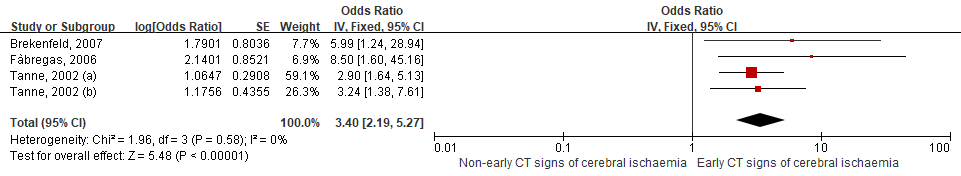
**

**sFigure 84: Forest plot showing the association between brain infarction volume and ICH**

**
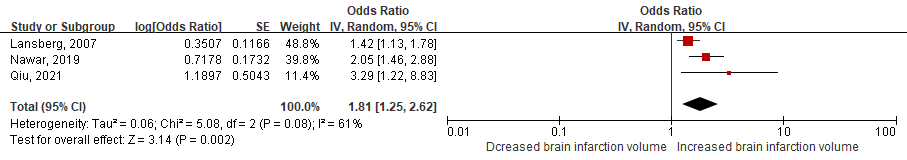
**

**sFigure 85: Forest plot showing the association between HPrs-PCT and ICH**

**
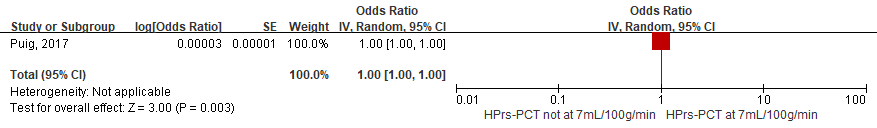
**

**sFigure 86: Forest plot showing the association between supratentorial territory PCA and ICH**

**
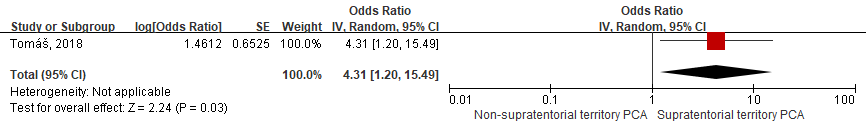
**

**sFigure 87: Forest plot showing the association between CBV and ICH**

**
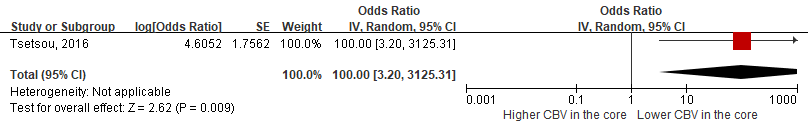
**

**sFigure 88: Forest plot showing the association between CV-L and ICH**

**
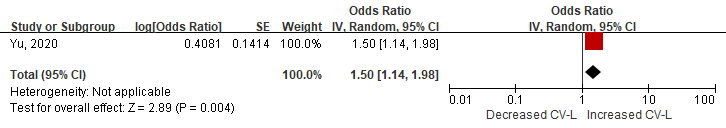
**

**sFigure 89: Forest plot showing the association between WMH and ICH**

**
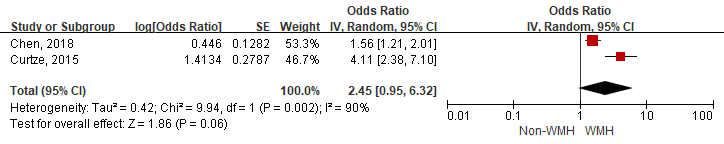
**

**sFigure 90: Forest plot showing the association between MMP-9-1562C/T polymorphism genotypes and ICH**

**
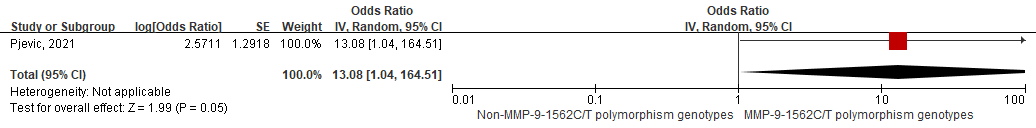
**

**sFigure 91: Forest plot showing the association between PAI-1 5G/5G genotype and ICH**

**
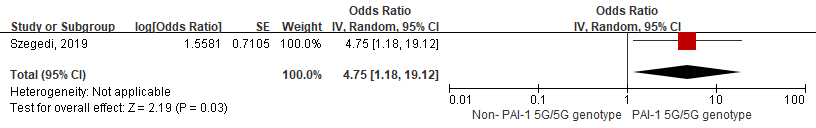
**

**sFigure 92: Forest plot showing the association between Rs1801020, C allele and ICH**

**
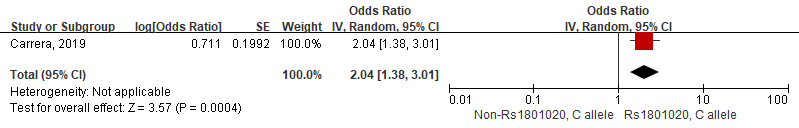
**

**sFigure 93: Forest plot showing the association between Rs669, A allele and ICH**

**
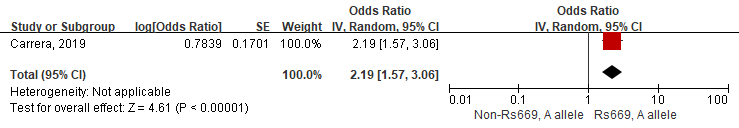
**

**sFigure 94: Forest plot showing the association between antithrombotic therapy and ICH**

**
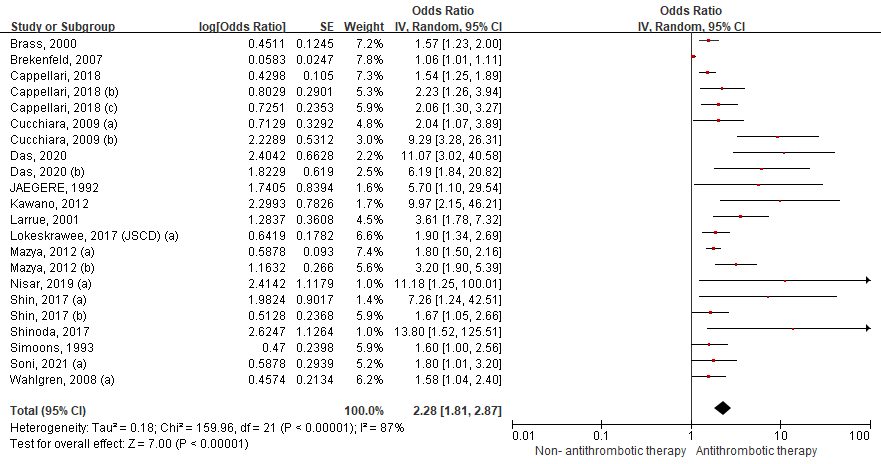
**

**sFigure 95: Forest plot showing the association between thrombolytic therapy and ICH**

**
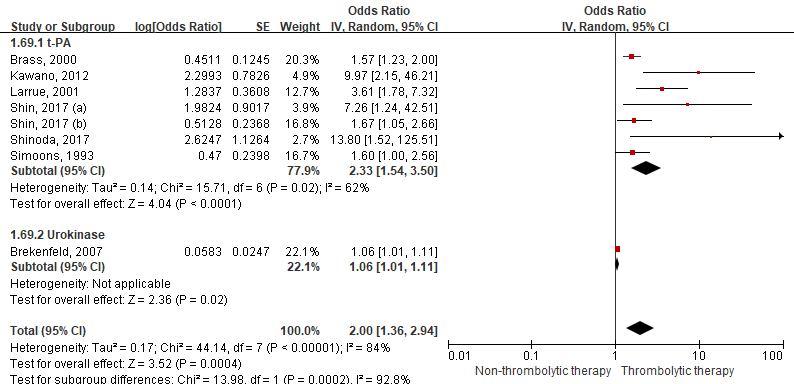
**

**sFigure 96: Forest plot showing the association between antiplatelet therapy and ICH**

**
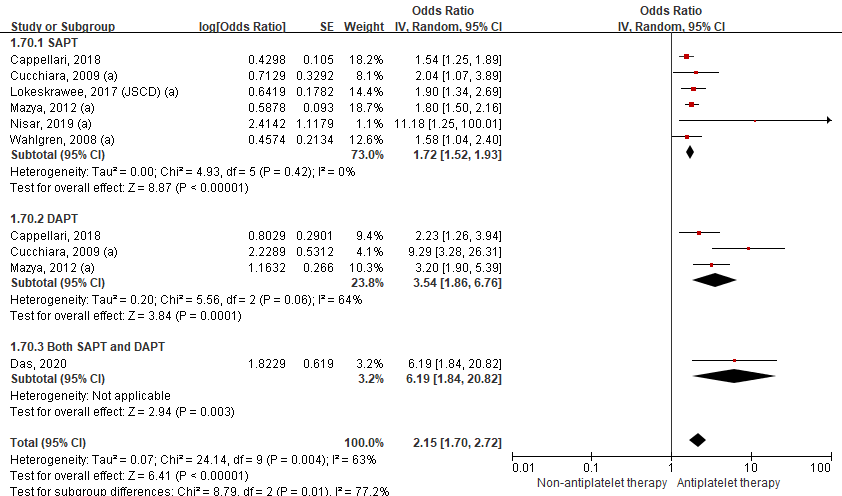
**

**sFigure 97: Forest plot showing the association between anticoagulant therapy and ICH**

**
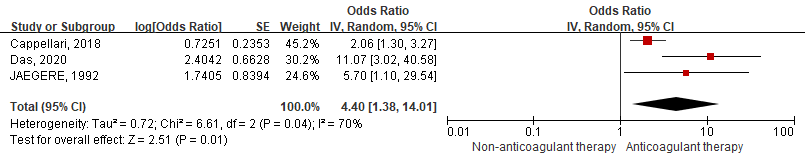
**

**sFigure 98: Forest plot showing the association between antihypertensive drugs and ICH**

**
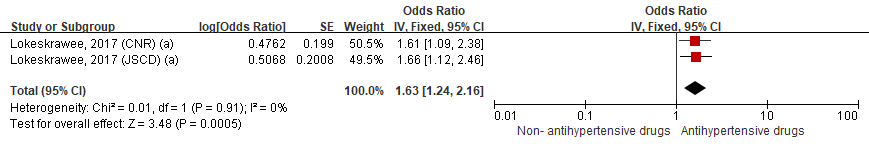
**

**sFigure 99: Forest plot showing the association between lipid-lowering drugs and ICH**

**
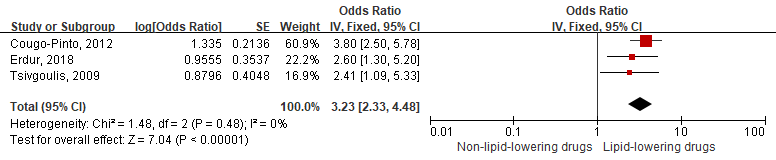
**

**sFigure 100: Forest plot showing the association between microcatheter injection (MCI) and ICH**

**
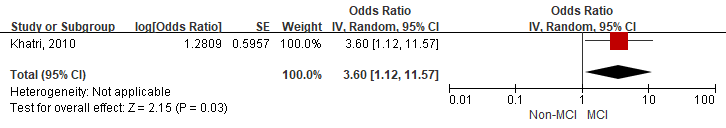
**

**sFigure 101: Forest plot showing the association between** **additional endovascular therapy and ICH**

**sFigure 102: Forest plot showing the association between deviation from the protocol and ICH**

**sFigure 103: Forest plot showing the association between periventricular transit time to the peak (TTP) and ICH**

**sFigure 104: Forest plot showing the association between vaspin and ICH**

**sFigure 105: Forest plot showing the association between time from onset to treatment (****OTT) and ICH**

**sFigure 106: Forest plot showing the association between time to recanalization and ICH**

**sFigure 107: Forest plot showing the association between age & NIHSS and ICH**

**sFigure 108: Forest plot showing the association between age & hypertension and ICH**

**sFigure 109: Forest plot showing the association between age & DBP and ICH**

**sFigure 110: Forest plot showing the association between** **age & body weight and ICH**

**Supplemental reference**

S1. Barber PA, Demchuk AM, Zhang J, Buchan AM. Validity and reliability of a quantitative computed tomography score in predicting outcome of hyperacute stroke before thrombolytic therapy. ASPECTS Study Group. Alberta Stroke Programme Early CT Score [published correction appears in Lancet 2000 Jun 17;355(9221):2170]. Lancet. 2000;355(9216):1670-1674.

S2. Kawano H, Hirano T, Nakajima M, Inatomi Y, Yonehara T, Uchino M. Modified ASPECTS for DWI including deep white matter lesions predicts subsequent intracranial hemorrhage. J Neurol. 2012;259(10):2045-2052.

S3. Singer OC, Kurre W, Humpich MC, et al. Risk assessment of symptomatic intracerebral hemorrhage after thrombolysis using DWI-ASPECTS. Stroke. 2009;40(8):2743-2748.

S4. del Río-Espínola A, Fernández-Cadenas I, Giralt D, et al. A predictive clinical-genetic model of tissue plasminogen activator response in acute ischemic stroke. Ann Neurol. 2012;72(5):716-729.

S5. Menon BK, Saver JL, Prabhakaran S, et al. Risk score for intracranial hemorrhage in patients with acute ischemic stroke treated with intravenous tissue-type plasminogen activator. Stroke. 2012;43(9):2293-2299.

S6. Tanne D, Kasner SE, Demchuk AM, et al. Markers of increased risk of intracerebral hemorrhage after intravenous recombinant tissue plasminogen activator therapy for acute ischemic stroke in clinical practice: the Multicenter rt-PA Stroke Survey. Circulation. 2002;105(14):1679-1685.

S7. Chatterjee S, Weinberg I, Yeh RW, et al. Risk factors for intracranial haemorrhage in patients with pulmonary embolism treated with thrombolytic therapy Development of the PE-CH Score. Thromb Haemost. 2017;117(2):246-251.

S8. Lokeskrawee T, Muengtaweepongsa S, Patumanond J, et al. Prediction of Symptomatic Intracranial Hemorrhage after Intravenous Thrombolysis in Acute Ischemic Stroke: The Symptomatic Intracranial Hemorrhage Score. J Stroke Cerebrovasc Dis. 2017;26(11):2622-2629.

S9. Mazya M, Egido JA, Ford GA, et al. Predicting the risk of symptomatic intracerebral hemorrhage in ischemic stroke treated with intravenous alteplase: safe Implementation of Treatments in Stroke (SITS) symptomatic intracerebral hemorrhage risk score [published correction appears in Stroke. 2012 Sep;43(9):e102]. Stroke. 2012;43(6):1524-1531.

S10. Wahlgren N, Ahmed N, Eriksson N, et al. Multivariable analysis of outcome predictors and adjustment of main outcome results to baseline data profile in randomized controlled trials: Safe Implementation of Thrombolysis in Stroke-MOnitoring STudy (SITS-MOST). Stroke. 2008;39(12):3316-3322.

S11. Saposnik G, Guzik AK, Reeves M, Ovbiagele B, Johnston SC. Stroke Prognostication using Age and NIH Stroke Scale: SPAN-100. Neurology. 2013;80(1):21-28.

S12. Cappellari M, Turcato G, Forlivesi S, et al. STARTING-SICH Nomogram to Predict Symptomatic Intracerebral Hemorrhage After Intravenous Thrombolysis for Stroke. Stroke. 2018;49(2):397-404.

S13. Flint AC, Faigeles BS, Cullen SP, et al. THRIVE score predicts ischemic stroke outcomes and thrombolytic hemorrhage risk in VISTA. Stroke. 2013;44(12):3365-3369.

S14. Flint AC, Rao VA, Chan SL, et al. Improved ischemic stroke outcome prediction using model estimation of outcome probability: the THRIVE-c calculation. Int J Stroke. 2015;10(6):815-821.

S15. Asuzu D, Nyström K, Amin H, et al. TURN: A Simple Predictor of Symptomatic Intracerebral Hemorrhage After IV Thrombolysis. Neurocrit Care. 2015;23(2):166-171.

S16. Zhou Z, Yin X, Niu Q, Liang S, Mu C, Zhang Y. Risk Factors and a Nomogram for Predicting Intracranial Hemorrhage in Stroke Patients Undergoing Thrombolysis. Neuropsychiatr Dis Treat. 2020;16:1189-1197.

S17. Erdur H, Polymeris A, Grittner U, et al. A Score for Risk of Thrombolysis-Associated Hemorrhage Including Pretreatment with Statins. Front Neurol. 2018;9:74.

S18. Kidwell CS, Saver JL, Carneado J, et al. Predictors of hemorrhagic transformation in patients receiving intra-arterial thrombolysis. Stroke. 2002;33(3):717-724.

S19. Soni M, Wijeratne T, Ackland DC. A risk score for prediction of symptomatic intracerebral haemorrhage following thrombolysis. Int J Med Inform. 2021;156:104586.

S20. Wu Y, Chen H, Liu X, et al. A new nomogram for individualized prediction of the probability of hemorrhagic transformation after intravenous thrombolysis for ischemic stroke patients. BMC Neurol. 2020;20(1):426.

S21. Simoons ML, Maggioni AP, Knatterud G, et al. Individual risk assessment for intracranial haemorrhage during thrombolytic therapy. Lancet. 1993;342(8886-8887):1523-1528.

S22. Guo H, Xu W, Zhang X, et al. A Nomogram to Predict Symptomatic Intracranial Hemorrhage After Intravenous Thrombolysis in Chinese Patients. Neuropsychiatr Dis Treat. 2021;17:2183-2190.

S23. Larrue V, von Kummer R R, Müller A, Bluhmki E. Risk factors for severe hemorrhagic transformation in ischemic stroke patients treated with recombinant tissue plasminogen activator: a secondary analysis of the European-Australasian Acute Stroke Study (ECASS II). Stroke. 2001;32(2):438-441.

S24. El Nawar R, Yeung J, Labreuche J, et al. MRI-Based Predictors of Hemorrhagic Transformation in Patients With Stroke Treated by Intravenous Thrombolysis. Front Neurol. 2019;10:897.

S25. Kufner A, Galinovic I, Brunecker P, et al. Early infarct FLAIR hyperintensity is associated with increased hemorrhagic transformation after thrombolysis. Eur J Neurol. 2013;20(2):281-285.

S26. Qiu M, Fang M, Liu X. Low free triiodothyronine levels predict symptomatic intracranial hemorrhage and worse short-term outcome of thrombolysis in patients with acute ischemia stroke. Medicine (Baltimore). 2017;96(45):e8539.

S27. Rocco A, Heuschmann PU, Schellinger PD, et al. Glycosylated hemoglobin A1 predicts risk for symptomatic hemorrhage after thrombolysis for acute stroke. Stroke. 2013;44(8):2134-2138.

S28. Lee C, Na JU, Lee JH, et al. Characteristics of blood tests in patients with acute cerebral infarction who developed symptomatic intracranial hemorrhage after intravenous administration of recombinant tissue plasminogen activator. Clin Exp Emerg Med. 2019;6(2):160-168.

S29. Puig J, Blasco G, Daunis-I-Estadella P, et al. High-permeability region size on perfusion CT predicts hemorrhagic transformation after intravenous thrombolysis in stroke. PLoS One. 2017;12(11):e0188238. Published 2017 Nov 28.

S30. Dharmasaroja PA, Muengtaweepongsa S, Pattaraarchachai J, Dharmasaroja P. Intracerebral hemorrhage following intravenous thrombolysis in Thai patients with acute ischemic stroke. J Clin Neurosci. 2012;19(6):799-803.

S31. Lin SF, Chao AC, Hu HH, et al. Low Cholesterol Levels Increase Symptomatic Intracranial Hemorrhage Rates After Intravenous Thrombolysis: A Multicenter Cohort Validation Study. J Atheroscler Thromb. 2019;26(6):513-527.

S32. Nisar T, Hanumanthu R, Khandelwal P. Symptomatic Intracerebral Hemorrhage after Intravenous Thrombolysis: Predictive Factors and Validation of Prediction Models. J Stroke Cerebrovasc Dis. 2019;28(11):104360.

S33. Asuzu D, Nyström K, Amin H, et al. Cohort-Based Identification of Predictors of Symptomatic Intracerebral Hemorrhage After IV Thrombolysis. Neurocrit Care. 2015;23(3):394-400.

S34. Khatri R, Khatri P, Khoury J, Broderick J, Carrozzella J, Tomsick T. Microcatheter contrast injections during intra-arterial thrombolysis increase intracranial hemorrhage risk. J Neurointerv Surg. 2010;2(2):115-119.

S35. Lansberg MG, Thijs VN, Bammer R, et al. Risk factors of symptomatic intracerebral hemorrhage after tPA therapy for acute stroke. Stroke. 2007;38(8):2275-2278.

S36. Lokeskrawee T, Muengtaweepongsa S, Patumanond J, et al. Prognostic Parameters for Symptomatic Intracranial Hemorrhage after Intravenous Thrombolysis in Acute Ischemic Stroke in an Asian Population. Curr Neurovasc Res. 2017;14(2):169-176.

S37. Dorňák T, Král M, Sedláčková Z, et al. Predictors for Intracranial Hemorrhage Following Intravenous Thrombolysis in Posterior Circulation Stroke [published correction appears in Transl Stroke Res. 2018 Mar 5;:]. Transl Stroke Res. 2018;9(6):582-588.

S38. Nagaraja N, Tasneem N, Shaban A, et al. Cerebral Microbleeds are an Independent Predictor of Hemorrhagic Transformation Following Intravenous Alteplase Administration in Acute Ischemic Stroke. J Stroke Cerebrovasc Dis. 2018;27(5):1403-1411.

S39. Ribo M, Montaner J, Molina CA, et al. Admission fibrinolytic profile is associated with symptomatic hemorrhagic transformation in stroke patients treated with tissue plasminogen activator. Stroke. 2004;35(9):2123-2127.

S40. Selim M, Fink JN, Kumar S, et al. Predictors of hemorrhagic transformation after intravenous recombinant tissue plasminogen activator: prognostic value of the initial apparent diffusion coefficient and diffusion-weighted lesion volume. Stroke. 2002;33(8):2047-2052.

S41. Dusanovic Pjevic M, Jekic B, Beslac Bumbasirevic L, et al. TT genotype of the MMP-9-1562C/T polymorphism may be a risk factor for thrombolytic therapy-induced hemorrhagic complications after acute ischemic stroke. Pharmacotherapy. 2021;41(7):562-571.

S42. Shinoda N, Hori S, Mikami K, et al. Prediction of hemorrhagic transformation after acute thrombolysis following major artery occlusion using relative ADC ratio: A retrospective study. J Neuroradiol. 2017;44(6):361-366.

S43. Kimura K, Iguchi Y, Shibazaki K, et al. Recanalization between 1 and 24 hours after t-PA therapy is a strong predictor of cerebral hemorrhage in acute ischemic stroke patients. J Neurol Sci. 2008;270(1-2):48-52.

S44. Cho BH, Kim JT, Chang J, Choi KH, Park MS, Cho KH. Prediction of hemorrhagic transformation in acute ischaemic stroke by micro- and macroalbuminuria after intravenous thrombolysis. Eur J Neurol. 2013;20(8):1145-1152.

S45. Chen Y, Yan S, Xu M, Zhong G, Liebeskind DS, Lou M. More extensive white matter hyperintensity is linked with higher risk of remote intracerebral hemorrhage after intravenous thrombolysis. Eur J Neurol. 2018;25(2):380-e15.

S46. Brekenfeld C, Remonda L, Nedeltchev K, et al. Symptomatic intracranial haemorrhage after intra-arterial thrombolysis in acute ischaemic stroke: assessment of 294 patients treated with urokinase. J Neurol Neurosurg Psychiatry. 2007;78(3):280-285.

S47. Das S, Mondal GP, Bhattacharya R, et al. Predictors of Postthrombolysis Outcome and Symptomatic Postthrombolysis Hemorrhage following Intravenous Thrombolysis with Alteplase for Acute Ischemic Stroke. J Neurosci Rural Pract. 2020;11(2):315-324.

S48. Zou M, Churilov L, He A, Campbell B, Davis SM, Yan B. Hyperdense middle cerebral artery sign is associated with increased risk of hemorrhagic transformation after intravenous thrombolysis for patients with acute ischaemic stroke. J Clin Neurosci. 2013;20(7):984-987.

S49. Gensicke H, Frih AA, Strbian D, et al. Prognostic significance of proteinuria in stroke patients treated with intravenous thrombolysis. Eur J Neurol. 2017;24(2):262-269.

S50. Yang C, Zhang J, Liu C, Xing Y. Comparison of the risk factors of hemorrhagic transformation between large artery atherosclerosis stroke and cardioembolism after intravenous thrombolysis. Clin Neurol Neurosurg. 2020;196:106032.

S51. Luo Y, Chen J, Yan XL, et al. Association of Non-Traditional Lipid Parameters with Hemorrhagic Transformation and Clinical Outcome After Thrombolysis in Ischemic Stroke Patients. Curr Neurovasc Res. 2020;17(5):736-744.

S52. Castellanos M, van Eendenburg C, Gubern C, et al. Low Levels of Caveolin-1 Predict Symptomatic Bleeding After Thrombolytic Therapy in Patients With Acute Ischemic Stroke. Stroke. 2018;49(6):1525-1527.

S53. Liu J, Wang D, Xiong Y, Yuan R, Tao W, Liu M. Low free triiodothyronine levels are related to symptomatic intracranial hemorrhage and poor functional outcomes after intravenous thrombolysis in acute ischemic stroke patients. Neurol Res. 2016;38(5):429-433.

S54. Acampa M, Camarri S, Lazzerini PE, et al. Increased arterial stiffness is an independent risk factor for hemorrhagic transformation in ischemic stroke undergoing thrombolysis. Int J Cardiol. 2017;243:466-470.

S55. Martí-Fàbregas J, Bravo Y, Cocho D, et al. Frequency and predictors of symptomatic intracerebral hemorrhage in patients with ischemic stroke treated with recombinant tissue plasminogen activator outside clinical trials. Cerebrovasc Dis. 2007;23(2-3):85-90.

S56. Huynh T, Cox JL, Massel D, et al. Predictors of intracranial hemorrhage with fibrinolytic therapy in unselected community patients: a report from the FASTRAK II project. Am Heart J. 2004;148(1):86-91.

S57. Lin SF, Chao AC, Hu HH, et al. Hyperglycemia predicts unfavorable outcomes in acute ischemic stroke patients treated with intravenous thrombolysis among a Chinese population: A prospective cohort study. J Neurol Sci. 2018;388:195-202.

S58. Mundiyanapurath S, Hees K, Ahmed N, et al. Predictors of symptomatic intracranial haemorrhage in off-label thrombolysis: an analysis of the Safe Implementation of Treatments in Stroke registry. Eur J Neurol. 2018;25(2):340-e11.

S59. Mendioroz M, Fernández-Cadenas I, Alvarez-Sabín J, et al. Endogenous activated protein C predicts hemorrhagic transformation and mortality after tissue plasminogen activator treatment in stroke patients. Cerebrovasc Dis. 2009;28(2):143-150.

S60. Cho AH, Kim JS, Kim SJ, et al. Focal fluid-attenuated inversion recovery hyperintensity within acute diffusion-weighted imaging lesions is associated with symptomatic intracerebral hemorrhage after thrombolysis. Stroke. 2008;39(12):3424-3426.

S61. Lin X, Cao Y, Yan J, et al. Risk Factors for Early Intracerebral Hemorrhage after Intravenous Thrombolysis with Alteplase. J Atheroscler Thromb. 2020;27(11):1176-1182.

S62. Kamal H, Mehta BK, Ahmed MK, et al. Laboratory factors associated with symptomatic hemorrhagic conversion of acute stroke after systemic thrombolysis. J Neurol Sci. 2021;420:117265.

S63. Piccardi B, Palumbo V, Nesi M, et al. Unbalanced Metalloproteinase-9 and Tissue Inhibitors of Metalloproteinases Ratios Predict Hemorrhagic Transformation of Lesion in Ischemic Stroke Patients Treated with Thrombolysis: Results from the MAGIC Study. Front Neurol. 2015;6:121. Published 2015 May 27.

S64. Cucchiara B, Kasner SE, Tanne D, et al. Factors associated with intracerebral hemorrhage after thrombolytic therapy for ischemic stroke: pooled analysis of placebo data from the Stroke-Acute Ischemic NXY Treatment (SAINT) I and SAINT II Trials. Stroke. 2009;40(9):3067-3072.

S65. Sledzińska-Dźwigał M, Sobolewski P, Szczuchniak W. Factors affecting the occurrence of symptomatic intracerebral haemorrhage after intravenous thrombolysis depending on the haemorrhage definition. Neurol Neurochir Pol. 2013;47(5):405-413.

S66. Yu Y, Zhang FL, Qu YM, et al. Intracranial Calcification is Predictive for Hemorrhagic Transformation and Prognosis After Intravenous Thrombolysis in Non-Cardioembolic Stroke Patients. J Atheroscler Thromb. 2021;28(4):356-364.

S67. Selker HP, Beshansky JR, Schmid CH, et al. Presenting pulse pressure predicts thrombolytic therapy-related intracranial hemorrhage. Thrombolytic Predictive Instrument (TPI) Project results. Circulation. 1994;90(4):1657-1661.

S68. Curtze S, Haapaniemi E, Melkas S, et al. White Matter Lesions Double the Risk of Post-Thrombolytic Intracerebral Hemorrhage. Stroke. 2015;46(8):2149-2155.

S69. Qiu F, Chen C, Fan Z, Qiu J, Chen Q, Shao B. White Matter Hypoperfusion Associated with Leukoaraiosis Predicts Intracranial Hemorrhage after Intravenous Thrombolysis. J Stroke Cerebrovasc Dis. 2021;30(2):105528.

S70. Neumann-Haefelin T, Hoelig S, Berkefeld J, et al. Leukoaraiosis is a risk factor for symptomatic intracerebral hemorrhage after thrombolysis for acute stroke. Stroke. 2006;37(10):2463-2466.

S71. Willer L, Havsteen I, Ovesen C, Christensen AF, Christensen H. Computed Tomography--Verified Leukoaraiosis Is a Risk Factor for Post-thrombolytic Hemorrhage. J Stroke Cerebrovasc Dis. 2015;24(6):1126-1130.

S72. Tsetsou S, Amiguet M, Eskandari A, et al. Severe cerebral hypovolemia on perfusion CT and lower body weight are associated with parenchymal haemorrhage after thrombolysis. Neuroradiology. 2017;59(1):23-29.

S73. Xu X, Li C, Wan T, et al. Risk Factors for Hemorrhagic Transformation After Intravenous Thrombolysis in Acute Cerebral Infarction: A Retrospective Single-Center Study. World Neurosurg. 2017;101:155-160.

S74. Tong X, George MG, Yang Q, Gillespie C. Predictors of in-hospital death and symptomatic intracranial hemorrhage in patients with acute ischemic stroke treated with thrombolytic therapy: Paul Coverdell Acute Stroke Registry 2008-2012. Int J Stroke. 2014;9(6):728-734.

S75. Brass LM, Lichtman JH, Wang Y, Gurwitz JH, Radford MJ, Krumholz HM. Intracranial hemorrhage associated with thrombolytic therapy for elderly patients with acute myocardial infarction: results from the Cooperative Cardiovascular Project. Stroke. 2000;31(8):1802-1811.

S76. Derex L, Hermier M, Adeleine P, et al. Clinical and imaging predictors of intracerebral haemorrhage in stroke patients treated with intravenous tissue plasminogen activator. J Neurol Neurosurg Psychiatry. 2005;76(1):70-75.

S77. Sun F, Liu H, Fu HX, et al. Predictive Factors of Hemorrhage After Thrombolysis in Patients With Acute Ischemic Stroke. Front Neurol. 2020;11:551157. Published 2020 Nov 3.

S78. Xing Y, Guo ZN, Yan S, Jin H, Wang S, Yang Y. Increased globulin and its association with hemorrhagic transformation in patients receiving intra-arterial thrombolysis therapy. Neurosci Bull. 2014;30(3):469-476.

S79. De Jaegere PP, Arnold AA, Balk AH, Simoons ML. Intracranial hemorrhage in association with thrombolytic therapy: incidence and clinical predictive factors. J Am Coll Cardiol. 1992;19(2):289-294.

S80. Tütüncü S, Ziegler AM, Scheitz JF, et al. Severe renal impairment is associated with symptomatic intracerebral hemorrhage after thrombolysis for ischemic stroke. Stroke. 2013;44(11):3217-3219.

S81. Jucevičiūtė N, Mikužis P, Balnytė R. Absolute blood eosinophil count could be a potential biomarker for predicting haemorrhagic transformation after intravenous thrombolysis for acute ischaemic stroke. BMC Neurol. 2019;19(1):127. Published 2019 Jun 13.

S82. Cougo-Pinto PT, Santos BL, Dias FA, et al. Frequency and predictors of symptomatic intracranial hemorrhage after intravenous thrombolysis for acute ischemic stroke in a Brazilian public hospital. Clinics (Sao Paulo). 2012;67(7):739-743.

S83. Muengtaweepongsa S, Prapa-Anantachai P, Dharmasaroja PA. Not only the Sugar, Early infarct sign, hyperDense middle cerebral artery, Age, Neurologic deficit score but also atrial fibrillation is predictive for symptomatic intracranial hemorrhage after intravenous recombinant tissue plasminogen activator. J Neurosci Rural Pract. 2017;8(1):49-54.

S84. Shin DH, Kang MJ, Kim JW, et al. The Impact of Discrepancy between Measured versus Stated Weight on Hemorrhagic Transformation and Clinical Outcomes after Intravenous Alteplase Thrombolysis. Cerebrovasc Dis. 2017;44(5-6):241-247.

S85. Kandzari DE, Granger CB, Simoons ML, et al. Risk factors for intracranial hemorrhage and nonhemorrhagic stroke after fibrinolytic therapy (from the GUSTO-i trial). Am J Cardiol. 2004;93(4):458-461.

S86. Wang R, Zeng J, Wang F, Zhuang X, Chen X, Miao J. Risk factors of hemorrhagic transformation after intravenous thrombolysis with rt-PA in acute cerebral infarction. QJM. 2019;112(5):323-326.

S87. Szegedi I, Nagy A, Székely EG, et al. PAI-1 5G/5G genotype is an independent risk of intracranial hemorrhage in post-lysis stroke patients. Ann Clin Transl Neurol. 2019;6(11):2240-2250.

S88. Sun X, Berthiller J, Trouillas P, Derex L, Diallo L, Hanss M. Early fibrinogen degradation coagulopathy: a predictive factor of parenchymal hematomas in cerebral rt-PA thrombolysis. J Neurol Sci. 2015;351(1-2):109-114.

S89. Vandelli L, Marietta M, Gambini M, et al. Fibrinogen decrease after intravenous thrombolysis in ischemic stroke patients is a risk factor for intracerebral hemorrhage. J Stroke Cerebrovasc Dis. 2015;24(2):394-400.

S90. Christoforidis GA, Karakasis C, Mohammad Y, Caragine LP, Yang M, Slivka AP. Predictors of hemorrhage following intra-arterial thrombolysis for acute ischemic stroke: the role of pial collateral formation. AJNR Am J Neuroradiol. 2009;30(1):165-170.

S91. Liu M, Pan Y, Zhou L, Wang Y. Predictors of post-thrombolysis symptomatic intracranial hemorrhage in Chinese patients with acute ischemic stroke. PLoS One. 2017;12(9):e0184646. Published 2017 Sep 18.

S92. Foerch C, Wunderlich MT, Dvorak F, et al. Elevated serum S100B levels indicate a higher risk of hemorrhagic transformation after thrombolytic therapy in acute stroke. Stroke. 2007;38(9):2491-2495.

S93. Marsh EB, Gottesman RF, Hillis AE, Urrutia VC, Llinas RH. Serum creatinine may indicate risk of symptomatic intracranial hemorrhage after intravenous tissue plasminogen activator (IV tPA). Medicine (Baltimore). 2013;92(6):317-323.

S94. Castellanos M, Leira R, Serena J, et al. Plasma cellular-fibronectin concentration predicts hemorrhagic transformation after thrombolytic therapy in acute ischemic stroke. Stroke. 2004;35(7):1671-1676.

S95. Che R, Huang X, Zhao W, et al. Low Serum Albumin level as a Predictor of Hemorrhage Transformation after Intravenous Thrombolysis in Ischemic Stroke Patients. Sci Rep. 2017;7(1):7776.

S96. Yao ES, Tang Y, Xie MJ, Wang MH, Wang H, Luo X. Elevated Homocysteine Level Related to Poor Outcome After Thrombolysis in Acute Ischemic Stroke. Med Sci Monit. 2016;22:3268-3273. Published 2016 Sep 15.

S97. Gnofam M, Leys D, Ponchelle-Dequatre N, et al. Baseline serum glucose concentration and symptomatic haemorrhagic transformation in non-diabetic stroke patients treated by intravenous thrombolysis. J Neurol. 2013;260(11):2786-2792.

S98. Rodríguez-González R, Blanco M, Rodríguez-Yáñez M, Moldes O, Castillo J, Sobrino T. Platelet derived growth factor-CC isoform is associated with hemorrhagic transformation in ischemic stroke patients treated with tissue plasminogen activator. Atherosclerosis. 2013;226(1):165-171.

S99. Tsivgoulis G, Frey JL, Flaster M, et al. Pre-tissue plasminogen activator blood pressure levels and risk of symptomatic intracerebral hemorrhage. Stroke. 2009;40(11):3631-3634.

S100. Palumbo V, Boulanger JM, Hill MD, Inzitari D, Buchan AM; CASES Investigators. Leukoaraiosis and intracerebral hemorrhage after thrombolysis in acute stroke. Neurology. 2007;68(13):1020-1024.

S101. James B, Chang AD, McTaggart RA, et al. Predictors of symptomatic intracranial haemorrhage in patients with an ischaemic stroke with neurological deterioration after intravenous thrombolysis. J Neurol Neurosurg Psychiatry. 2018;89(8):866-869.

S102. Li Y, Xia Y, Chen H, et al. Focal Low and Global High Permeability Predict the Possibility, Risk, and Location of Hemorrhagic Transformation following Intra-Arterial Thrombolysis Therapy in Acute Stroke. AJNR Am J Neuroradiol. 2017;38(9):1730-1736.

S103. Zhu B, Yan L, Ren H, Li Q, Chen X. Predictive Value of Apelin and Vaspin on Hemorrhagic Transformation in Patients with Acute Ischemic Stroke after Intravenous Thrombolysis and Analysis of Related Factors. Evid Based Complement Alternat Med. 2021;2021:5020622.

S104. Guo Z, Yu S, Xiao L, et al. Dynamic change of neutrophil to lymphocyte ratio and hemorrhagic transformation after thrombolysis in stroke. J Neuroinflammation. 2016;13(1):199.

S105. Lakhter V, Zack CJ, Brailovsky Y, et al. Predictors of intracranial hemorrhage in patients treated with catheter-directed thrombolysis for deep vein thrombosis. J Vasc Surg Venous Lymphat Disord. 2021;9(3):627-634.e2.
